# Single-target high-throughput transcription analyses reveal high levels of alternative splicing present in the FPPS/GGPPS from *Plasmodium falciparum*

**DOI:** 10.1038/srep18429

**Published:** 2015-12-21

**Authors:** Heloisa B. Gabriel, Mauro F. de Azevedo, Giuseppe Palmisano, Gerhard Wunderlich, Emília A. Kimura, Alejandro M. Katzin, João M. P. Alves

**Affiliations:** 1 Department of Parasitology, Institute of Biomedical Sciences, University of São Paulo, São Paulo, Brazil

## Abstract

Malaria is a tropical disease with significant morbidity and mortality. A better understanding of the metabolism of its most important etiological agent, *Plasmodium falciparum*, is paramount to the development of better treatment and other mitigation measures. Farnesyldiphosphate synthase/geranylgeranyldiphosphate synthase (FPPS/GGPPS) is a key enzyme in the synthesis of isoprenic chains present in many essential structures. In *P. falciparum*, as well as a handful of other organisms, FPPS/GGPPS has been shown to be a bifunctional enzyme. By genetic tagging and microscopy, we observed a changing localization of FPPS/GGPPS in blood stage parasites. Given the great importance of alternative splicing and other transcriptional phenomena in gene regulation and the generation of protein diversity, we have investigated the processing of the FPPS/GGPPS transcript in *P. falciparum* by high-throughput sequencing methods in four time-points along the intraerythrocytic cycle of *P. falciparum*. We have identified levels of transcript diversity an order of magnitude higher than previously observed in this organism, as well as a few stage-specific splicing events. Our data suggest that alternative splicing in *P. falciparum* is an important feature for gene regulation and the generation of protein diversity.

Malaria is one of the most important infectious diseases in the world, causing about 200 million clinical cases and nearly 600 thousand deaths every year. The causative agent of malaria is a protozoan of the genus *Plasmodium*, transmitted by the female *Anopheles* mosquito host, in which the sexual phase of the parasite’s life cycle takes place^1^. Of the five *Plasmodium* species that infect humans, *P. falciparum* is responsible for the vast majority of severe forms of the disease, including deaths. The increasing resistance of this parasite to virtually all current drugs, such as artemisinin and its derivates in five South-East Asian countries and probably in South America^2^, calls for combined therapy using drugs to which the parasites have not yet developed resistance, as well as for identifying new drug targets^3^. Therefore, the exact knowledge about parasite metabolic pathways is crucial for a better understanding of the parasite’s physiology and, consequently, the development of new chemotherapeutics.

An important target for the development of new antimalarial drugs is isoprenoid biosynthesis (Fig. 1), which occurs via the 2-C-methyl-D-erythritol-4-phosphate (MEP) pathway^4^,^5^,^6^,^7^,^8^ in *P. falciparum*, some plants, and most bacteria^9^,^10^. In contrast, most animal cells, certain eubacteria, archaea, and fungi synthesize isoprenoid precursors through the mevalonate pathway^11^. Many types of isoprenoids biosynthesized by *P. falciparum*, such as carotenoids, menaquinone, and α-tocopherol^8^,^12^,^13^,^14^, are essential components of the cellular machinery of many organisms, participating in a variety of biological processes. All isoprenoids are derived from a common precursor, isopentenyldiphosphate (IPP), and its isomer dimethylallyldiphosphate (DMAPP). Farnesyldiphosphate synthase (FPPS), which belongs to a family of enzymes classified as prenyltransferases, catalyzes the consecutive head-to-tail condensation of IPP with DMAPP to form geranyldiphosphate (GPP). Then, a second condensation between GPP and IPP forms farnesyldiphosphate (FPP). In *P. falciparum*, this enzyme (PF3D7_1128400) is bifunctional, having geranylgeranyldiphosphate synthase (GPPS) activity able to condense FPP with a further molecule of IPP to form the 20-carbon isoprenoid geranylgeranyldiphosphate (GGPP) (Fig. 1)^15^. FPP and GGPP function as substrates for the first reaction of several branched pathways leading to the synthesis of compounds such as ubiquinone, dolichol, menaquinone, carotenoids, and prenylated proteins. FPPS and GGPPS are the most studied prenyltransferases and have been described in various organisms from all three domains, Eukarya, Bacteria, and Archaea^16^. In protist parasites, the FPPS and/or GGPPS genes from *Trypanosoma cruzi*^17^, *Trypanosoma brucei*^18^, *Plasmodium vivax*^19^, and *Toxoplasma gondii* were identified^20^. In this last case, the enzyme has been described as a bifunctional enzyme, and this is also true for *P. falciparum.* In the case of malaria parasites, especially the most virulent species, *P. falciparum*, a number of new “plant-like” enzymes participating in the isoprenoid pathway were recently discovered. Some of these enzymes are associated with the apicoplast^21^, an organelle acquired by a secondary symbiosis^22^ and absent in mammalian cells. This obviously turns all structures contained therein prime targets for chemotherapeutic intervention.

Transcriptome analysis has shown that alternative splicing plays an important role generating a large number of mRNA and protein isoforms^23^,^24^,^25^. Recently, it was observed that the occurrence of alternative splicing events in *P. falciparum* is not as rare as previous studies had reported^26^,^27^, and is actually widespread also in other Apicomplexa such as *Toxoplasma gondii*, where it has been recently shown to be carefully regulated by the parasite^25^. Although 7,406 introns have been predicted in the *P. falciparum* genome, alternative splicing that might affect protein function has been observed only for a few genes^26^. Alternative splicing can result in modulation of transcript expression levels by subjecting mRNAs to nonsense-mediated decay (NMD) by stop codon addition, or in alteration of the structure of the gene product by changing, deleting or inserting amino acids in the protein, therefore influencing their intracellular localization and modifying their enzymatic activity and/or protein stability^23^.

Studies have reported the occurrence of several isoforms of the FPPS and/or GGPPS, through alternative splicing events, in some organisms^28^. Martin *et al.*^29^ showed that the FPPS gene undergoes alternative splicing in mammals leading to two different transcripts: a transcript encoding an isoform containing an extension of 66 amino acids with an N-terminal peptide that targets the mitochondria, while the smaller transcript is directed to the cytosol. In *P. falciparum*, antisense transcripts corresponding to FPPS/GGPPS have been recently demonstrated in sexual forms of the parasite^30^.

In next generation sequencing efforts of the transcriptome and epigenome of *P. falciparum* sexual and asexual stages, including samples from clinical isolates^30^,^31^,^32^,^33^, a large number of additional intron-exon splicing junctions missed by the initial genome annotation have been reported. Also, antisense transcripts and alternative splicing events were encountered and provided improved EST coverage and genome annotation.

In this study, during analysis of the localization of the protein FPPS/GGPPS, we observed different patterns of localization along the intra-erythrocytic cycle of the parasite, which led to the hypothesis that alternative splicing might be contributing to these differences. Thus, we have used the 454 sequencing platform for deep mRNA sequencing of the FPPS/GGPPS gene exclusively, using material from four time points of intraerythrocytic stages representing rings (R), early trophozoites (ET), late trophozoites (LT), and schizonts (S). We have detected high levels of alternative splicing of the FPPS/GPPS transcript, including possibly stage-specific, alternatively spliced isoforms.

## Results and Discussion

Farnesyl pyrophosphate synthase/geranylgeranyl pyrophosphate synthase (FPPS/GGPPS) is a major bifunctional enzyme of the isoprenoid pathway which belongs to the prenyltransferase family. It is a branch-point enzyme responsible for elongation of the isoprene chain. Changes in FPPS/GGPPS activity could alter the flux of isoprenoids towards various branches of this pathway and, hence, play a crucial role in the regulation of isoprenoid metabolism^28^. IPP and DMAPP are required to synthesize isoprenoid products, and Yet and DeRisi 2011^34^ demonstrated that the biosynthesis of the isoprenoid precursor is not only essential for the parasite but in fact the sole essential function of the apicoplast during blood-stage growth.

### Protein localization

**Figure 2:**
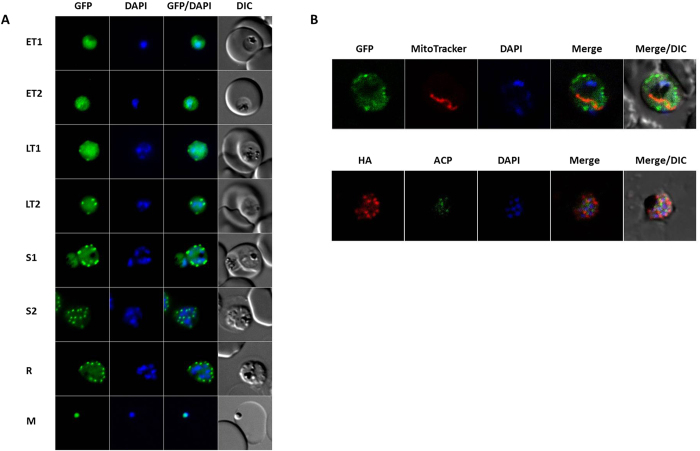
FPPS/GGPPS localization. Images of live parasites (FPPS/GGPPS-HA-GFP strain) expressing GFP and immunofluorescence of the FPPS/GGPPS-HA strain visualized by fluorescence or confocal microscopy. (**A**) Images of live parasites FPPS/GGPPS-HA-GFP by fluorescence microscopy during the intra-erythrocytic cycle of the parasite. (**B**) Bottom -immunofluorescence of the FPPS/GGPPS-HA strain with antibody as indicated, visualized by confocal microscopy. Top—Images of live FPPS/GGPPS-HA-GFP parasites with markers as indicated, visualized by confocal microscopy. ET1 (early trophozoites); ET2 (early trophozoites); LT (latetrophozoites); LT (latetrophozoites) S1 (schizont); S2 (schizont); R (rosettes); M (Merozoites); DAPI (nucleus marker). Original magnification for all images: 1.000×.

Using a transgenic parasite line where FPPS/GGPPS is expressed in fusion with an HA tag, we have previously demonstrated that the enzyme is present throughout the asexual stages in the intra-erythrocytic cycle^15^. In order to determine its localization in live parasites, another transgenic line was generated where FPPS/GGPPS is expressed in fusion with GFP-HA (data not shown). Analysis by fluorescence microscopy of live parasites confirms expression along the intra-erythrocytic cycle and shows FPPS/GGPPS localization throughout the cytoplasm and also forming spots, which increase in number as parasites mature from trophozoite to schizont stages (Fig. 2A). To investigate to which subcellular compartment the detected spots correspond, parasites labeled with the mitochondrial marker MitoTracker were similarly analyzed (Fig. 2B, upper panel). A distinct pattern is detected, suggesting FPPS/GGPPS does not colocalize with the mitochondria. Fixed parasites were analyzed by immunofluorescence using antibodies against the HA tag and the apicoplast marker acyl carrier protein (ACP)^35^, showing that the enzyme does not colocalize with the apicoplast either (Fig. 2B, bottom panel). These organelles are the location of the precursors^36^, products^34^ or enzymes^12^ already described in the isoprenoid pathway in *Plasmodium falciparum.* Cytosolic localization of FPPS was demonstrated in *Leishmania major*^37^, *Trypanosoma cruzi* and *Trypanosoma brucei*^38^. However, in *Toxoplasma gondii*, whose enzyme is also characterized as bifunctional, FPPS is located in the mitochondria^20^. Since isoforms generated through alternative splicing events have been shown to be important in cellular targeting of this protein, it could be speculated that a similar phenomenon might be taking place in *P. falciparum*. In many organisms, such as plants^39^, mammals^29^ and insects^40^ more than one isoform for FPPS and/or GGPPS is present, with different localization patterns. In *Toxoplasma gondii* the presence of two isoforms has been demonstrated. Interestingly, the transcription level of one is much higher than that of the other isoform, but these relative expression levels do not vary between tachyzoite and bradyzoite stages of the parasite^20^.

### Transcript sequencing and mapping

Gene regulation through alternative splicing is more versatile than regulation through promoter activity^23^. Some full-length cDNA and EST data have been used for analysis of transcript structure and variants, improving genome annotation and gene models in *P. falciparum*^30^,^31^,^32^,^33^. RNA-Seq provides the advantage of capturing an entire transcriptome at potentially great depth, enabling detection of low copy number transcripts and variants^32^. We demonstrate for the first time, to the best of our knowledge, a specific RNA-Seq analysis for just one gene (FPPS/GGPPS), from four time-points in the intraerythrocytic cycle of the parasite. Otto *et al.* 2010^31^ suggest that roughly 90% of this parasite’s genome is transcriptionally active during this stage. Sequencing of the amplified transcripts from each of the four libraries yielded good coverage, with about 26.8 to 44.3 million bases sequenced, distributed throughout about 49,000 (ring), 81,000 (early trophozoite), 54,000 (late trophozoite), or 76,000 (schizont) reads, for an average of between 519 and 621 bases per read, as expected for the sequencing technology employed. After quality trimming, only between 300 to 900 reads per library (or about 1% of the total, at the most) were discarded due to having regions of average quality lower than 20 or trimmed read shorter than 30 bases. Since the average read length was between 519 and 621 bases, the window used by the trimmer (sickle) was typically about 52 to 62 bases in length. Overall sequence coverage is shown in Additional file 1 and, as expected due to the average read sequence length compared to the full transcript size, it is clear that sequence coverage is lowest in the mid-point of the gene, between exons 6 and 7. Nonetheless, minimum coverage is still quite high (around 5,000-fold). Coverage at intronic regions (gray bands) is obviously mostly absent and is represented by flat lines in parts of the graph where there are no sequenced bases (Add. file 2). Even in this coarse representation, it is already possible to identify some of the alternative splicing events described below, like the partial retention of the end of intron 2, most evident in the schizont stage, or the partial retention of the end of intron 7, well distinguishable in all stages.

The aligner (STAR) was able to map between 88% and 94% of the trimmed reads to the reference sequence, which consisted of the full length FPPS/GGPPS gene from start to stop codon, and including all annotated introns, as currently annotated. Using STAR’s output files, isoform_threader was able to identify 329 putative splice junction regions, which after manual inspection dropped to 98 high confidence predictions (Additional file 2). All subsequent analyses were performed using these selected junctions. Intron donor and acceptor splicing sites were classified as canonical (GT…AG) or alternative (anything else). Canonical junctions accounted for 78, while alternative ones accounted for 252, of all junctions originally seen; after filtering, these numbers dropped to 58 and 40, respectively. This strongly suggests that most low confidence alternative splicing junctions originally seen were produced by sequencing and/or alignment errors eliminated by manual curation.

### Stage-specific junctions

**Figure 3:**
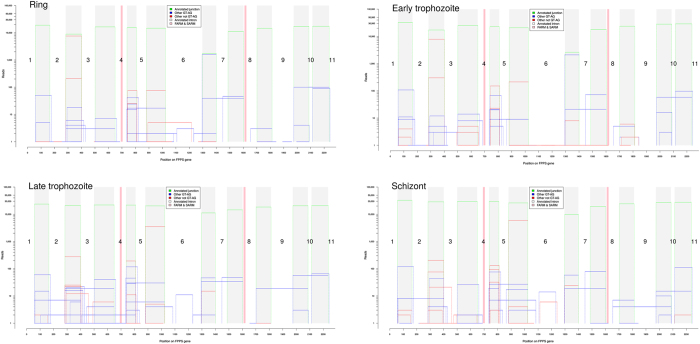
Splice junctions from FPPS/GGPPS and their relative quantities during the intra-erythrocytic cycle. (**A**) Ring; (**B**) Early trophozoite – 24 hours; (**C**) Late trophozoite – 35 hours; (**D**) Schizont. Green line – annotated junction; blue line – other GT-AG junctions; red line – other junctions (not GT-AG); white bars – annotated exons, numbered; pink bars – FARM and SARM domains.

*P. falciparum* has a complex life cycle with different functional characteristics associated with specific gene expression patterns^41^. Thus, the observation of stage-specific transcriptional differences suggests that these new splice junctions are likely associated with some unique features of the parasite’s developmental stages, and probably with isoprenoid pathway regulation. López-Barragán *et al.* 2011^30^ demonstrated 201 alternative splicing events affecting 178 genes in *P. falciparum*, and, of these, 124 isoforms occurred in a stage-specific manner. Figure 3 shows the different splice junctions, and their relative quantities (see also Additional file 2), found for each of the life-cycle stages. Our results observed a total of 98 high confidence predictions splice junction regions for FPPS/GGPPS and, among these, 63 are only present in one or two stages of the parasite cycle analyzed (Additional file 2). The most abundant novel junctions appear in all four life cycle stages analyzed, such as junctions 179 or 199, which delete exon 7 or parts thereof, respectively, or 238 and 248, which delete exon 10 or part of exon 11, respectively. Many other junctions, on the other hand, are distributed in a stage-specific way, such as junction 2 (deletes part of exon 1), which is absent in the ring stage, or junction 17, which deletes exon 2 (absent in early trophozoite and schizont), or junctions 76 and 77, exclusively seen in late trophozoite, and which delete about half of exon 3 or that plus the whole of exon 4, respectively. We can also observe isoforms present only in the schizont stage, such as 216, which deletes a part of exon 8 and the whole of exon 9. Apart from a few splice junction combinations (see below), most of the new junctions introduced stop codons a few residues thereafter, suggesting either a much shorter final protein product or, more likely, the production of an mRNA that will be subjected to nonsense-mediated decay and not generate any protein product – in which case the function of these alternative splicing events might be purely regulatory. As described below, we have validated a number of these variants by other methods.

### Viable isoforms

**Figure 4:**
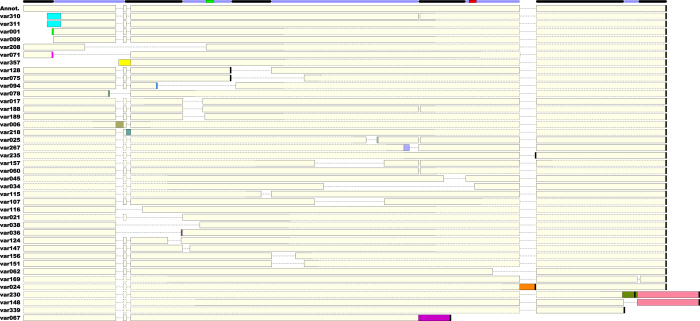
Potentially viable isoforms of the FPPS/GGPPS. Isoforms possibly allowing the translation to a functional protein. The top isoform is the annotated version. Exon positions are marked by alternating black and blue bars on the top, and the FARM and SARM are represented by rectangles in exons 4 and 8, respectively. The five in-frame stop codons, as well as the final stop in the predicted proteins, are represented in black. Different colors along sequence segments in the variants denote difference compared to the annotated isoform. Dashed lines around the boxes represent regions that have been added to “pad” incomplete variants, as described in the main text.

Most of the novel splice junctions identified in this work seem to, by themselves and absent any other variant or compensating transcriptional or translational phenomenon, lead to frame-shifts and therefore numerous stop codons that would terminate translation soon after the splice junction – a possibility exacerbated by *P. falciparum*’s very high AT-content. However, we identified 40 cases (Fig. 4 and Additional files 3, 4, and 5) where is it potentially possible to get a reasonable protein product (i.e., the length of the final protein produced is at least half of that of the annotated protein). Given the huge number of combinatorial possibilities (about 3.63 billion, in our estimate) for generating isoforms using the splice junctions identified in this work, we have only analyzed variant combinations that have been experimentally seen in our sequencing data. Since no single 454 read can cover a complete FPPS/GGPPS transcript, and most cover at best half of the predicted complete transcript, we have “padded” variant isoforms with sequence processed according to the splice junctions of the annotated variant (Additional files 3 and 5). This was done by complementing each novel splice junction combination (after manual curation) with the expected remaining sequence, according to the annotated variant, in the relevant direction; in the very few cases where sequence was missing on both ends, we added the missing parts to both ends of the sequenced variant. In that way, it was possible to generate predicted variants that present sequences starting from exon 1 and ending as close as possible to exon 11, regardless of the direction in which they were originally sequenced, which allows us to compare variants while taking into account their context in the full length of the gene. While it is definitely possible that other combinations of the putative novel splice junctions could also occur in the cell, we preferred to be more conservative and analyzed solely those combinations actually seen in reads that have been sequenced, even if that could lower the number of potentially viable protein products seen in our results. The exact splice junction composition of each variant analyzed, as well as the count of each variant in each of the four life-cycle stages analyzed here, can be found in Additional file 4.

As can be seen in Fig. 4, most variants display deletion in parts of one or more exons, with only a few presenting the insertion of short new sequences – with the exception of variants var230, var148, and var067. The last one, incidentally, is the only variant that terminates significantly before the previously annotated stop codon, leading to the complete removal of exons 8 through 11. One variant (var357) lacks a significant portion of the N-terminal region, and is also the only one without an obvious candidate for starting codon. Four other variants present slightly different N-terminal regions compared to the annotated isoform, which could potentially have significance for protein targeting in the cell (although our searches using *in silico* cellular targeting predictors did not show a significant difference between the scores of these variants).

### Stop codon readthrough

One in-frame stop codon was observed in each of five of the potentially viable isoforms – although in the case of var148, it occurs just 38 amino acids before the end of the protein. This observation raises the possibility of in-frame stop codon readthrough^42^ in the FPPS/GGPPS transcripts in *P. falciparum*. This phenomenon, recently shown to be common in organisms like *Drosophila* and others^43^,^44^ and shown to be strictly regulated^45^, consists of the reading of a stop codon as a signal for the incorporation of an amino acid instead.

Variants var75, var128, and var148 present UGA as the in-frame stop codon. As previously seen, at first mainly in viruses but later also in cellular organisms from all domains of life, one of the mechanisms of in-frame stop codon readthrough is recoding, from a UGA stop codon to selenocysteine^42^. The analysis of the *P. falciparum* genome^46^ has shown features that support the possibility of stop codon readthrough. This would lead to the incorporation of selenocysteine to proteins in this parasite, which is supported by the presence of a selenocysteinyl-tRNA, proteins necessary for the selenocysteine insertion machinery, and also conserved putative selenocysteine-insertion sequences (SECIS) found in the 3′-UTR of four *Plasmodium*-specific genes^47^,^48^. Considering that *P. falciparum* presents very limited redundancy in its tRNA gene repertoire^46^, it seems significant that a selenocysteinyl-tRNA has been retained in the genome. It has also been observed experimentally that supplementation of the culture medium with selenium increases the parasite growth rate^49^. Together, these observations strongly suggest the importance of selenoproteins for the parasite, and thus the need for in-frame UGA stop codon recoding. Whether this phenomenon is actually happening in the novel FPPS/GGPPS transcripts characterized here is uncertain until confirmation based on further, more suitable experimental data analyzing the actual proteins produced.

The other two variants (var024 and var235) presenting an in-frame stop codon have a UAG, located exactly between exons 8 and 9. In the case of var024 there is an insertion, exclusive to this variant, of nine amino acids (IIIIIFFFL) immediately before the in-frame stop codon. Besides the above-mentioned selenocysteine incorporation, other amino acids have been observed replacing an in-frame stop codon, employing other, less well-characterized mechanisms. Particularly, UAA and UAG codons have been detected as coding for glutamine, tyrosine, or lysine, whereas tryptophan, cysteine, and arginine were coded for by a UGA codon^44^. Thus, if readthrough is indeed happening in the newly described isoforms, it might be occurring a different number of ways. Again, more experimental data focusing on the actual proteins will help clarify these questions.

### Functional domains

Amino acid sequence alignment of FPPS from different organisms revealed conserved regions I to VII with two characteristic aspartate rich motifs, one in region II called FARM (first Asp-rich motif) and in region VI called SARM (second Asp-rich motif)^15^. A hydrophilic side chain at the fifth amino acid upstream of the FARM region plays a crucial role in the production of both GGPP and FPP^50^. This region determines the final products of all *E*-isoprenyldiphosphate synthases and can thus be designated as the chain-length determination (CLD) region^50^. It has been shown that the presence of a cysteine at the fifth position is essential for the FPPS/GGPPS bifunctionality in *T. gondii*^20^ for example, and a bulky phenylalanine at this same position in the methanobacterial version of this enzyme also leads to the production of GGPP and FPP^51^. Narita *et al.*^52^ showed that, in *B. stearothermophilus*, FPP synthase could be converted to GPP synthase by the substitution of phenylalanine by serine at the fourth position before the FARM. In *P. falciparum*, the FARM occurs in the middle of exon 4, while the SARM occurs very close to the beginning of exon 8.

In this work we can observe one isoform (var094) that deletes the FARM and two (var034 and var067) that delete the SARM (Fig. 4), indicating that the corresponding proteins are transcribed in their entirety without these regions, and with probable loss of FPPS and/or GGPPS function. Thus, one of the major functions of these variants generated by alternative splicing events may be the regulation in the formation of the main precursors of the isoprenoid pathway: GPP, FPP or GGPP.

### Exon-skipping, partial deletion, or new intron creation

A splicing variant type of significant importance is exon-skipping, where an exonic sequence that is usually present in the mature transcript is removed together with its flanking introns, resulting in a shorter transcript. In this work, we have observed a number of potential partial exon deletions or complete exon-skipping events – where part of or the whole of the exon gets removed, respectively (Additional files 2 and 3, and Fig. 4). We have also seen three cases of new intron creation, where a sequence internal to a previously annotated exon gets removed from the transcript; one of these events involved exon 2, and the other two involved exon 6.

**Figure 5:**
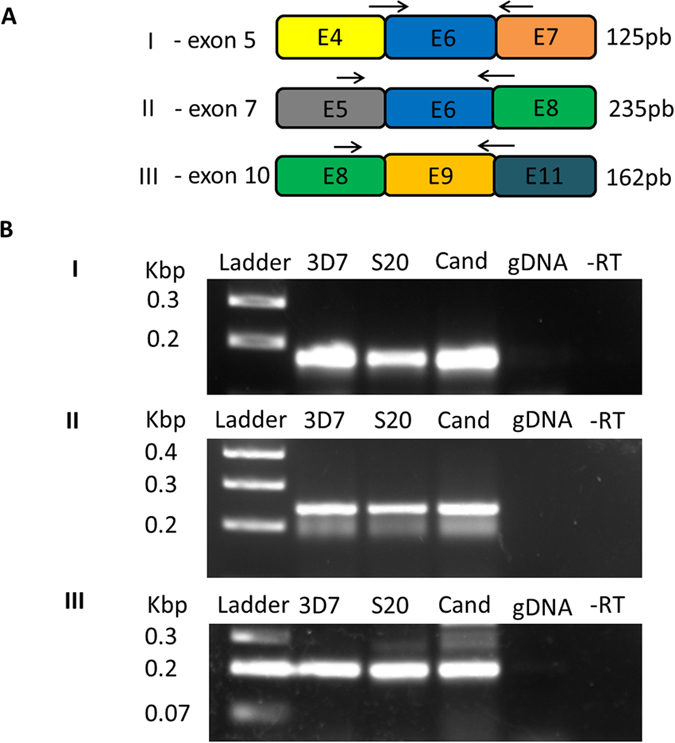
Reverse-transcriptase PCR essays for confirming isoforms in the reference strain and in strains that were isolated from patients. (**A**) Primers were designed to target regions of a size specific for detecting deletion of a particular exon. (**B**) I—Amplification products obtained for primers in A I (exon 5 deleted). II—Amplification products obtained for primers in A II (exon 7 deleted). III—Amplification products obtained for primers in A III (exon 10 deleted). Isolates: 3D7 (reference strain); S20 (isolated from a patient); (**C**) and (isolated from a patient); gDNA (genomic DNA from 3D7); -RT (reaction without reverse transcriptase for 3D7); Ladder (GeneRuler 1 kb Plus, Thermo Scientific).

**Figure 6:**
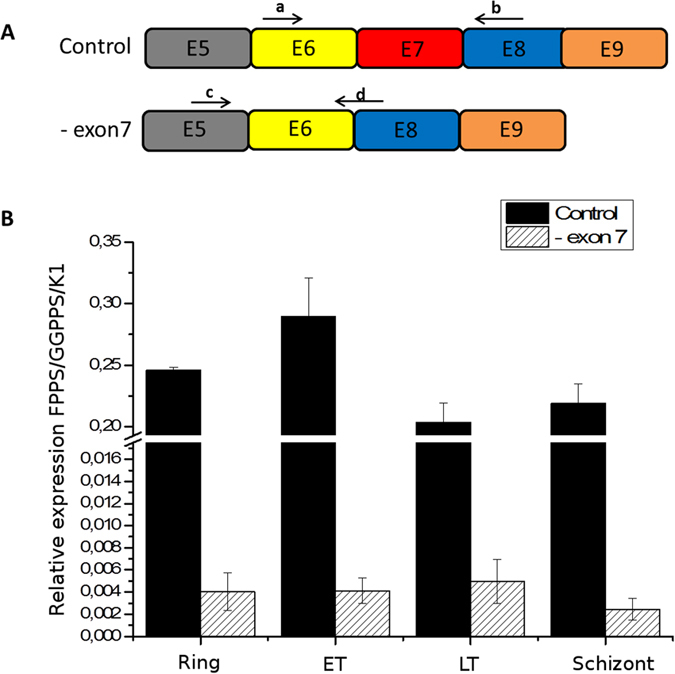
qPCR comparison of expression between isoforms lacking exon 7 or generating the intact primary protein. (**A**) Oligonucleotide design for detecting an alternative splicing event; a and b (control primers that flank exon 7); c and d (primers designed to detect deletion of exon 7). (**B**) Transcript levels of the gene encoding FPPS/GGPPS in isolate 3D7 were normalized by the control gene K1. Statistical significance was determined by one-way ANOVA. All differences between control and variant isoforms were significant (*p* < 0.05). Control (transcript for the primary protein); -exon7 (isoform lacking exon 7); a and b (F-iso and R-iso); c and d (F-iso7 and R-iso7); ET (earlytrophozoite); LT (late trophozoite).

To validate the intron-exon junctions and alternatively spliced events detected here by RNA-Seq, we randomly selected three of the novel splicing junctions (Fig. 5). Primers flanking the newly described splice sites were used for PCR amplification using cDNA from the schizont stage of isolate 3D7 (culture and extraction independent from those used for RNA-Seq). In addition, the same amplification was also performed using two recently obtained field isolates from patients, in order to discard the possibility of any artifacts due to strains being kept in culture for extended periods, as is 3D7’s case. This experiment confirmed the occurrence of these three alternative splicing events, both in 3D7 and the two recent field isolates, and also corroborated the data quantification by RT-PCR, indicating that the isoform(s) lacking exon 7, for example, are about 100 times less expressed than those presenting it (Fig. 6). And finally, as further validation, we have confirmed the alternative splicing events involving the deletions of exons 7 and 10 by Sanger sequencing of the respective cDNA PCR products (data not shown).

### Intron retention

Intron retention is the splicing variant event where a piece of sequence that is usually removed from the primary transcript is included in the mature transcript. We have investigated the presence of intron retention in FPPS/GGPPS from all four stages present in this study. To try to minimize the number of false positives due to the possible sequencing of unprocessed transcripts, we have only considered those events occurring in reads that presented at least one other intron removed from the transcript. Table 1 presents the number of putative intron retention events observed in our FPPS/GGPPS reads, and all sequences containing retained introns are present in Additional file 5. Retention events of introns 1, 2, or 4 were significantly different between stages of the intraerythrocytic cycle of the parasite, while introns 7, 8, and 10 presented similar (low) levels of retention in all stages. Introns 3, 5, 6, and 9 were not involved in any retention event at all. It is interesting to note that introns 1, 2, and 4 have much higher GC content (15.25 to 16.67%) than the other introns that have not been seen retained, or that have been retained in very low numbers (less than 10%, with the exception of intron 6, which presented 14.42% GC content); the complete spliced transcript, as annotated, presents 27.85% GC content. Although our data does not allow for a conclusion on the reason for this discrepancy, two hypotheses present themselves: either the higher GC is a cause of higher retention (e.g. by changing the overall sequence characteristics of the intron and thus lowering splicing efficiency); or the higher GC of the retained introns is a consequence of higher retention (by having to maintain a certain coding potential for the amino acids inserted when the intron is retained). Further clarification on which, if any, of these hypotheses is a better explanation for the putative intron retention seen in this gene will depend on adequate experimental data.

Certain life-cycle stages seemed to be more intensely involved in different retention events (Table 2). Thus, the schizont retention count was significantly different from counts from other stages for intron 1 (but the other three stages had similar counts to each other), for example. For intron 4, the retention count for late trophozoite was the outlier. For intron 2, on the other hand, the early trophozoite retention count was significantly different from both ring and schizont counts, but not from the late trophozoite count. It is not clear why certain introns, especially those closer to the start of the transcript, are much more retained than others (Table 1). However, the fact that intron retention seems to be stage-specific suggests that these events may have regulatory importance. Previous work reported intron retention cases in *P. falciparum* which occurs in less than 50% of the analyzed transcripts^32^,^53^. Our data support these conclusions, showing that this is among the rarest categories of alternative splicing in these organisms, and again emphasizing the importance of the new splice junctions in the regulation and development of the parasite.

The only intron retention event that could eventually lead to a functional protein is the one involving intron 1, which introduces an in-frame stop codon (UAA) about halfway through the translated intron sequence. As mentioned above for other kinds of splicing variants, this could be another case of in-frame stop codon-readthrough leading to extra proteins in the organism. Although the significance of this extra sequence is not completely clear at present, it is very interesting to note that a search for targeting signal with TargetP indicates a very high (0.974) NN score for “other” location (i.e. not mitochondria, chloroplasts, nor secreted), while the annotated isoform presents a slightly lower score of 0.881. Further experimental investigation of this possible variant could show whether it is contributing to the concentration of FPPS/GGPPS in the spots seen in the cytoplasm described above.

### Antisense transcripts

*P. falciparum* variability in transcription of the FPPS/GGPPS enzyme had been previously demonstrated in sexual stages by López-Barragán *et al.* 2011^30^, in the antisense transcript category. Antisense transcripts are non-coding RNAs that may act predominantly as regulators of sense gene expression^30^. Genes with antisense transcription have an increased number of annotated isoforms compared to genes without antisense transcription, as described in several organisms, such as amphibians, fish, insects, birds, nematodes, and mammals^54^. In the RNA masking model, an antisense RNA may mask a splice site on the sense pre-mRNA sequence, leading to an alternative splicing event^30^. Due to the nature of the method we used, which employs primers to PCR-amplify only one gene in particular, the description of the likely occurring antisense transcripts was not possible in this work.

### Proteomic investigation

In order to investigate whether any of the isoforms present in the parasite got translated into protein, we have analyzed parasite protein extracts by mass spectrometry. From a total of 17 samples from the SDS-PAGE, with proteins in the size range of 30 to 50 kDa, we obtained a total of 1,383 high confidence peptides. In our results, we have found proteins already well-studied in this parasite, such as MSP2—merozoite surface protein-2^55^ or EXP-2—Exported protein-2, a vacuolar membrane protein exposed into the vacuolar space^56^. We also found SR1 protein, a pre-mRNA splicing factor involved in regulation of alternative splicing in *Plasmodium falciparum*^57^.

More significantly, as an internal control, is the fact that we have found, in the 41 kDa gel band, three high confidence peptides from the annotated version of the FPPS/GGPPS protein. Taken together, these observations show that our data correctly reflects the expected proteome of *P. falciparum*.

On the other hand, we have failed to detect any peptide mapping to variant protein forms predicted from the alternatively spliced transcripts uncovered here. Given the sensitivity limits of the proteomic methodology employed, and taking into account that the annotated isoform is almost always more than 1,000 times more abundant than any of the variants in the transcriptome (Fig. 3), it is likely that a different, more sensitive method must be used in order to detect any proteins potentially derived from variant isoforms of the FPPS/GGPPS gene in *P. falciparum*.

## Conclusions

FPPS/GGPPS is an essential enzyme, responsible for the regulation of a whole pathway that is extremely important for the survival of the parasite, as previously mentioned^15^,^34^. Alternative splicing events in *P. falciparum*, as seen in many other organisms, are important mechanisms that both work in increasing protein diversity and possible functionalities beyond what could be afforded by the organism’s gene complement by itself. Although our data does not directly allow for conclusions in that regard, it is possible to speculate that such variation could also contribute to differences in protein localization throughout the cell, as well as provide significant post-transcriptional gene regulation. In this work, we have examined in detail the transcriptional products for the FPPS/GGPPS gene, uncovering unprecedented variability in splicing of this *P. falciparum* gene. This suggests that proteomic complexity in this organism could be much higher than estimated by looking at the relatively small annotated genome, and more in line with what seems necessary for a complex life-cycle like the one displayed by malaria parasites.

## Methods

### Organism growth conditions

Cultures of *P*. *falciparum* clone 3D7 were grown as described^58^, except that human serum was replaced with Albumax I (0.5%, Invitrogen/Life Technologies). Parasite development and multiplication were monitored by microscopic evaluation of Giemsa-stained thin smears.

### Plasmid construction

The integration vector pFPPS/GGPPS-HA used herein is described elsewhere^15^. The HA tag of this vector was replaced with the sequence encoding a GFP-HA fusion, retrieved from pEF-Luc-GFP-HA-DD24^59^, generating pFPPS/GGPPS-GFP-HA.

### Parasite transfection

Parasites were transfected as previously described^60^, using the electroporation conditions established elsewhere^61^. Briefly, *P. falciparum* 3D7 was cultured in 4% hematocrit in RPMI-HEPES supplemented with 0.5% Albumax 1 (Invitrogen). Ring stage parasites at 5–8% parasitemia were transfected with 150 μg of pFPPS/GGPPS-GFP-HA and submitted to drug pressure with 2.5 nM WR99210 starting two days later. Parasites were cultivated in standard conditions until parasites re-appeared and normal growth was re-established. The integration at the genomic FPPS/GGPPS locus was selected by intermittent exposure and retrieval of WR99210 and checked by PCR under standard conditions using oligonucleotides inside and outside the integrated locus. Parasites transfected with pFPPS/GGPPS-HA are described in Jordão *et al.*^15^.

### Immunofluorescence

For immunofluorescence analysis, we have followed the protocol described by Furtado *et al.*^62^ with modifications. Infected erythrocytes (5–10% parasitemia in 10% hematocrit) were fixed with 3.7% formaldehyde/PBS for 3 hours, permeabilized with 0.1% BSA, 0.005% PBS-saponin for 25 min twice and incubated with α-HA primary antibody (1:100 dilution, Sigma), and α-ACP primary antibody (1:200 dilution) diluted in 0.1% BSA, 0.001% saponin/PBSfor 1 hour at 37 °C and finally incubated with their secondary antibody Alexa Fluor® 488 diluted in 0.1% BSA, 0.001% saponin/PBS for 45 min at 37 °C. After washing, the material was dried in air for immunofluorescence slides prepared with Vectashield and then analyzed by confocal microscopy (LSM 780-NLO).

For analysis with live parasites expressing GFP, we have utilized Mitotracker (1:10000 dilution, Molecular Probes) for 30 min at 37 °C and then visualized by fluorescence microscopy (Zeiss LSM710) or confocal microscopy (LSM 780-NLO).

### cDNA preparation; PCR and RT-PCR

The total RNA of intraerythrocytic stages of *P. falciparum* (ring – at 10 hours, early trophozoite — at 24 hours, late trophozoite – at 35 hours, and schizont – at 45 hours) was extracted using TRIZOL LS (Invitrogen) following manufacturer instructions. The washed RNA pellet was briefly dried at room temperature and dissolved in water and stored at −80 °C until use. About 4 μg of total RNA were used for cDNA synthesis. Briefly, total RNA was treated 3 times with DNAse I (Fermentas) prior to synthesis to prevent genomic DNA contamination. Treated RNA was reverse transcribed using MuLV-Revert Aid reverse transcriptase (Fermentas) and random hexamer primers following the manufacturer’s instructions. Oligonucleotides (F-FPPS/GGPPS-BamHI; R-FPPS/GGPPS-PstI – Additional file 6) that amplify the transcript that encodes the FPPS enzyme were designed using the Primer3 server (http://frodo.wi.mit.edu/). PCR reactions were performed with Taq polymerase enzyme (Invitrogen) using the following program: 95 °C for 5 minutes; 35 cycles of 95 °C for 40 seconds; annealing temperature of 58 °C for 40 seconds; 72 °C for 90 seconds; followed by a final extension at 72 °C for 10 minutes.

For confirmation experiments, oligonucleotides (F-iso5; R-iso5—F-iso7; R-iso7—F-iso10; R-iso10 – Additional file 6) that target regions specific for detection of a particular alternative splicing event were designed using Primer3. PCR reactions were performed with the Taq polymerase enzyme (Invitrogen) using the following program: 95 °C for 5 minutes; 35 cycles of 95 °C for 40 seconds; annealing temperatures of: 59 °C for F-iso5-R-iso5; 57 °C for F-iso7-R-iso7, and 59 °C for F-iso10-R-iso10 for 40 seconds; 72 °C for 90 seconds; followed by a final extension at 72 °C for 10 minutes.

For RT-PCR experiments, the oligonucleotides (control – F-iso-R-iso and F-iso7-R-iso7 – Additional file 6) amplified with the same performance regarding the internal control gene (±1 Ct), seryl-tRNAsynthetase (PF3D7_0717700^63^). Quantitative PCR assays were performed using the SYBR Green Mix (Thermo Scientific), using the PCR template in a StepOne Real-Time PCR machine (Applied Biosystems). All reactions were performed in triplicate permitting not more than 0.5 units deviation between individual Ct values. Samples with Cts over 35 were considered as unamplified targets. Dissociation curves of amplicons were analyzed for all reactions, where each amplicon has a specific temperature characterizing the specificity of the obtained products. The relative transcript abundance for each pair of primers was determined independently by subtracting the Ct value measured for each sample from the Ct value of the internal control target using the formula 2^−ΔCt^ 64.

### High-throughput sequencing

Amplified FPPS/GGPPS transcripts were sequenced to high depth using the 454 GS FLX + platform (performed by Macrogen Inc., South Korea), with expected read lengths of about 600–800 bases on average. For each of the four intra-erythrocytic time-points, one sequence library was constructed using 25 ng/μl for ring, 75 ng/μl for early trophozoite, 45 ng/μl for late trophozoite and 65 ng/μl for schizonts of the amplification product. The sequencing was designed to generate an average of 50,000 reads per library. All sequencing data has been submitted to the Short Read Archive (http://www.ncbi.nlm.nih.gov/sra), and are accessible under accession numbers SRR2180188 (ring), SRR2180184 (early trophozoite), SRR2180186 (late trophozoite), and SRR2180187 (schizont).

### Transcript sequence analysis

Resulting transcript sequences were quality trimmed by sickle^65^ using a minimum average quality of 20 and minimum trimmed read length of 30 bases. Trimmed reads were mapped to the full FPPS/GGPPS gene (locus tag PF11_0295, GeneID:810842) using STAR^66^, with visualization and coverage analysis performed in Tablet^67^. Graphs of sequence coverage and splice junction positioning and abundance along the gene were generated using R (http://www.r-project.org/). STAR results pertaining to intron boundaries were analyzed using software developed specifically for this work (isoform_threader, available at http://isoformthreader.sourceforge.com/), which uses the SJ (splice junction) and SAM files generated by STAR, plus the original FASTQ files containing the raw transcript sequencing reads, and i) generates a report file detailing how many reads support each splice junction seen (both those found in the SJ file and those not, due to not being one of the three known junction sequence combinations); ii) identifies all combinations of splice junctions and the reads supporting them; and iii) separates reads to different files according to which splice junction combination they support. The identification of the potential intron boundaries are identified from the N operations in the SAM file’s CIGAR strings. Each and every putative novel splice junction was manually examined (using Tablet) in alignments against the full, annotated gene in order to discard any junction that was too close to alignments ends, as well as any junction near homopolymer regions longer than three bases (which are particularly error-prone in pyrosequencing).

To quantitatively characterize events of intron retention, all FPPS/GGPPS intronic sequences (as defined in the annotated isoform) were searched by BLASTN against the full gene sequence (low complexity filter turned off, 95% minimum identity threshold, 1E-30 maximum E-value threshold), and only instances where the whole intron is contained within the read were scored as positive. Also, a minimum of 30 (25 in the case of the first and last exons) bases aligning in the flanking exon were required to avoid false positives from bad alignments, and a read supporting intron retention must have at least one other intron removed, to minimize the chance of false positives due to unprocessed transcripts being sequenced.

All statistical analyses of splice junction or intron retention event counts were performed in R by using Fisher’s exact test with Bonferroni-corrected threshold P-values (using 0.05 as the family-wide error rate).

Potential cellular targeting signals were investigated using TargetP 1.1^68^, with organism group set to “plant”.

### SDS-PAGE separation and in gel-digestion

Asynchronous cultures of *P*. *falciparum* were recovered and treated with 0.15% saponin in RPMI media to release hemoglobin from the red blood cells. Proteins were extracted with buffer: 0.05 M Tris–HCl, pH 6.8, 10% glycerol, 2 mM EDTA, 2% SDS, 0.05% bromophenol blue, 50 mM dithiothreitol^69^ for separation by SDS-PAGE. Seventeen bands, differing by about 1 kDa each, were cut from the gel, ranging from 30 to 50 kDa.

Excised gel bands were in-gel digested with trypsin as previously described^70^. Briefly, gel bands were minced and destained using 100 mM ammonium bicarbonate/acetonitrile (1:1, vol/vol), followed by neat acetonitrile to shrink the gel. Disulfide bonds were reduced by 10 mM DTT at 56 °C for 30 min and alkylated with 40 mM iodoacetamide for 30 min at room temperature in the dark. Proteins were digested with sequencing grade trypsin (Promega) overnight at 37 °C. Tryptic activity was quenched by TFA acidification. Peptides were extracted by acetonitrile/water and desalted and concentrated on ZipTip C18-microcolumns (Millipore) according to manufacturer instructions.

### Mass spectrometry analysis

Peptides were separated by nano-LC-MS/MS on an Acclaim^®^ PepMap100 15 cm × 75 μm packed with C18 material (3 μm; 100 Å, Thermo Fisher) using an Easy-LC nano-HPLC (Thermo Fisher). The HPLC gradient was 0–35% solvent B (A = 0.1% formic acid; B = Acetonitrile, 0.1% formic acid) in a total of 70 min run at a flow of 250 nL/min. Mass spectrometric analysis was performed using an LTQ OrbitrapVelosETD (Thermo Scientific, Bremen, Germany). An MS scan (400–2000*m*/z) was recorded in the Orbitrap mass analyser at a resolution of 60,000 at 400*m*/*z* for a target of 10^6^ ions followed by data-dependent collision-induced dissociation (CID) MS/MS analysis of the top twenty most intense ions with charge state ≥ 2.The following parameters: activation time = 10 ms, normalized energy = 35, Q-activation = 0.25, dynamic exclusion = enabled with repeat count 1, exclusion duration = 30 s and, intensity threshold = 10,000, target ions = 10^4^.

### Proteome Data Analysis

Raw files were analyzed using Proteome Discoverer v1.4 (Thermo Scientific). MS/MS spectra were converted to .mgf files and searched against the UniProtKB *Plasmodium* database (March 2015) using Sequest HT search engine. Database searches were performed with the following fixed parameters: precursor mass tolerance 10 ppm; MS/MS mass tolerance 0.6 Da and full trypsin cleavage with two possible missed cleavages. Fixed modifications: cysteine carbamidomethylation. Variable modifications included: methionine oxidation. Shared peptide sequences were reported as protein grouped accessions. False discovery rates were obtained using Percolator^71^ selecting identification with a q-value equal or less than 0.01. A minimum of two peptides was chosen to identify a protein.

## Additional Information

**How to cite this article**: Gabriel, H. B. *et al.* Single-target high-throughput transcription analyses reveal high levels of alternative splicing present in the FPPS/GGPPS from *Plasmodium falciparum.* *Sci. Rep.* **5**, 18429; doi: 10.1038/srep18429 (2015).

## Supplementary Material

Supplementary Files

## Figures and Tables

**Figure 1:**
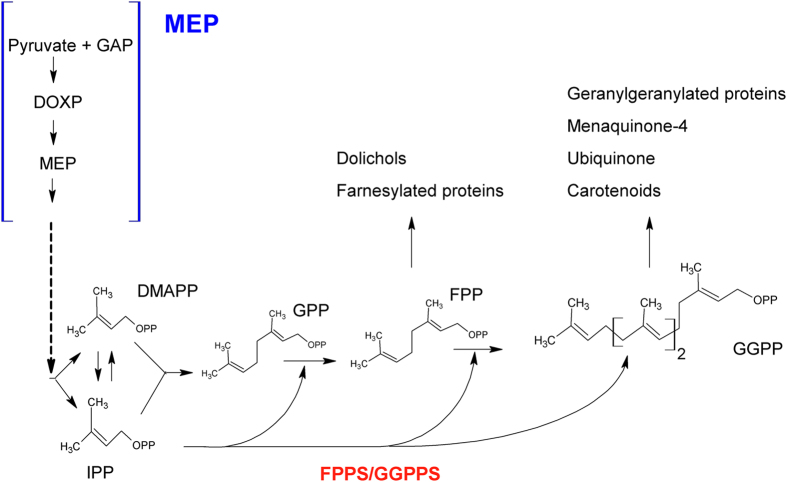
Schematic diagram of the isoprenoid biosynthesis in *P. falciparum*. Downstream of the MEP pathway, all isoprenoids are derived from a common precursor, IPP, and its DMAPP isomer. FPPS/GGPPS catalyzes the consecutive condensation of IPP with DMAPP to form GPP. Then, a second condensation between GPP and IPP forms FPP. In *P. falciparum* FPPS/GGPPS condenses FPP with a further molecule of IPP to form GGPP.

**Table 1:** FPPS/GGPPS intron retention events in four *P. falciparum* stages.

	Ring	Schizont	Early tropho	Late tropho	Ring no	Schizont no	ET no	LT no
Intron 1	31	155	43	20	30,648	35,261	48,270	24,389
Intron 2	14	22	4	4	28,029	31,344	40,393	22,250
Intron 3								
Intron 4	31	18	21	61	27,000	33,069	38,097	22,862
Intron 5								
Intron 6								
Intron 7	1	5	0	1	11,593	23,046	18,994	17,064
Intron 8	6	4	0	3	15,300	28,808	24,926	21,584
Intron 9								
Intron 10	0	1	5	0	18,122	32,245	30,220	24,091
Total reads	48,944	76,087	80,727	54,352				

First four columns of numbers present amount of reads presenting intron retention; remaining four columns show how many reads did not present retention. Total read numbers are for reference only and were not used in the statistical analyses. Statistical analysis (two-sided Fisher exact test, alpha = 0.05/6 = 0.008) of differential number of retention events: Intron 1: significant (P < 2.2 × 10^−16^); Intron 2: significant (P = 5.3 × 10^−5^); Intron 4: significant (P = 3.7 × 10^−13^); Intron 7: not significant (P = 0.149); Intron 8: not significant (P = 0.011); Intron 10: not significant (P = 0.041).

**Table 2:**
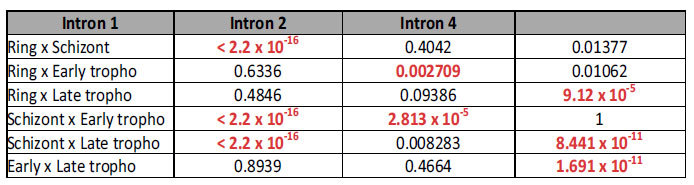
Intron retention comparison between intra-erythrocytic forms.

Only the three introns whose retention numbers were statistically significant in the global analysis are included. All pairwise comparisons of life cycle stage retention numbers were performed with a level of significance alpha = 0.05/18 = 0.0028. Note: numbers in bold and red typeface indicate significant difference (Fisher’s exact test P-value below 0.0028).
