# Supplementary material for: Single-target high-throughput transcription analyses reveal high levels of alternative splicing present in the FPPS/GGPPS from Plasmodium falciparum
Source: Sci Rep. 2015 Dec 21;5:18429. doi: 10.1038/srep18429 (PMC4685265; doi:10.1038/srep18429)
Supplement: Supplementary Files [file srep18429-s1.pdf]

Single-target high-throughput transcription analysis reveal high levels of alternative splicing present in the FPPS/GGPPS from *Plasmodium falciparum*

Heloisa B. Gabriel, Mauro F. de Azevedo, Giuseppe Palmisano, Gerhard Wunderlich, Emília A. Kimura, Alejandro M. Katzin, João M. P. Alves

ADDITIONAL FILES

Additional file 1

Title: Overall sequence coverage from RNA-seq.

Description: Coverage levels indicate number of reads covering each position along the annotated *P. falciparum* FPPS/GGPPS gene. Coverage along intronic regions is usually represented by a line following from the last available exonic coverage, with the exception of cases where some intronic sequence was added to an exon (see main text). Each life-cycle stage is represented by a line, according to the legend. Vertical white areas represent annotated exons (numbered).

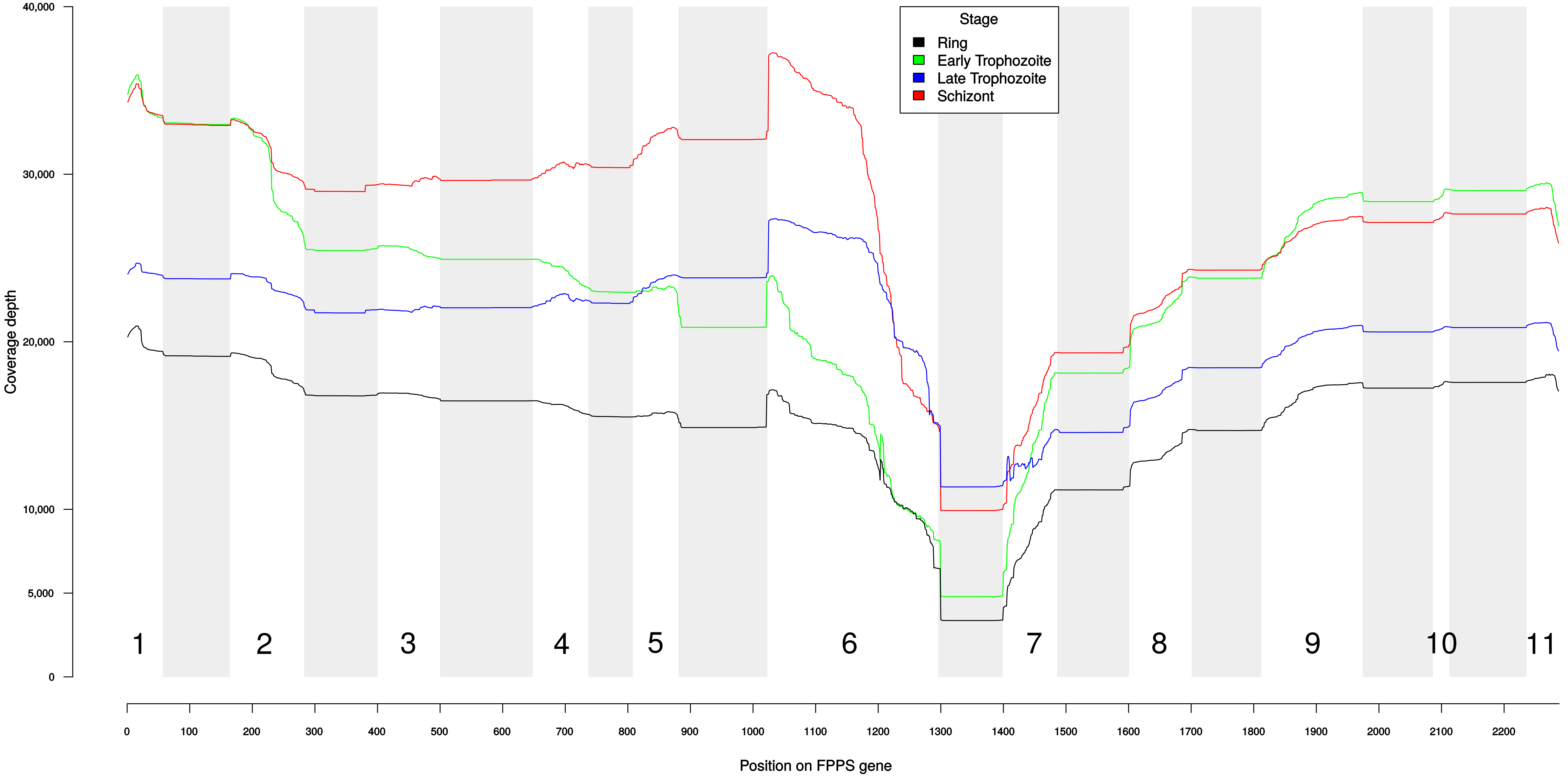

Stage
Ring
Early Trophozoite
Late Trophozoite
Schizont
Coverage depth
40,000
30,000
20,000
10,000
5,000
0
1
2
3
4
5
6
7
8
9
10
11
0
100
200
300
400
500
600
700
800
900
1000
1100
1200
1300
1400
1500
1600
1700
1800
1900
2000
2100
2200
Position on FPPS gene

Additional file 2

Title: High confidence splice junctions

Description: Splice junction identifiers, coordinates, and junction types are represented, as well as number of reads where each junction has been observed in each of the four life-cycle stages studied here. Notes indicate predicted effect each alternative junction might have on an eventual protein product.

Junction	Start	End	Ring	ET	LT	Schizont	Type	Notes
2	38	165	0	4	8	1		deletes about 1/3 of the end of exon 1
5	56	401	0	1	0	8		deletes 2 bases at the end of exon 1 plus the whole of exon 2
10	58	131	0	1	0	0	GT:TG	
11	58	154	0	2	2	2	GT:TG	
**12**	**58**	**165**	**19045**	**32788**	**23630**	**32627**		
14	58	176	50	108	60	119		deletes 11 bases at the start of exon 2
15	58	182	0	9	1	0		
17	58	401	1	0	7	0		deletes exon 2
19	61	165	1	4	0	3	AG:AG	
20	62	165	5	11	15	8		
25	71	165	0	0	0	1		
30	103	401	0	0	2	0		
32	193	253	1	0	0	0	AT:CT	creates a new intron inside exon 2, removing about ½ of its sequence
33	214	401	0	0	0	1		
34	222	689	0	0	0	1	AG:GC	
35	266	401	0	1	0	0	TT:AG	
44	283	401	207	300	25	76	GG:AG	"stage-specific error" (4 instead of 5 G)
47	284	649	3	3	19	0		
48	284	685	0	0	1	0		deletes exon 3 and part of exon 4
52	284	389	0	0	0	3	GT:TT	
53	284	400	7575	7914	276	204	GT:TA	"stage-specific error" (6 instead of 5 G)
**54**	**284**	**401**	**8917**	**17169**	**21215**	**28506**		
55	284	417	6	5	16	4		deletes part of exon 3
56	284	428	4	1	22	4		deletes part of exon 3
58	285	410	0	0	22	0	TA:AA	
63	293	401	18	12	20	43		adds 9 bases to the end of exon 2
67	293	459	0	0	12	21	AG:TA	
69	322	401	1	3	6	0		
73	338	809	0	0	1	0		adds about ½ of intron 2 to exon 2, deletes exons 3 and 4
75	405	649	0	0	2	0		
76	445	649	0	0	4	0		deletes about ½ of exon 3
77	445	809	0	0	2	0		deletes about ½ of exon 3 plus the whole of exon 4
78	474	646	0	0	0	2	AA:TT	
81	491	649	0	0	6	0	GC:AG	
83	493	654	1	3	0	2	TT:GG	
84	495	654	0	0	0	1	GT:GG	
92	500	652	0	5	0	0	GG:AT	
**94**	**501**	**649**	**16451**	**24863**	**21934**	**29546**		
95	501	650	0	1	0	0	GT:GC	
96	501	661	7	14	40	26		deletes 12 bases at the start of exon 4
97	501	685	1	8	1	1		deletes about ¼ at the start of exon 4

98	501	688	0	0	1	2		deletes about ¼ at the start of exon 4
103	734	809	15	20	45	19		deletes 4 bases at the end of exon 4
104	734	1024	0	0	6	0		deletes end of exon 4 plus the whole of exon 5
109	736	822	0	0	1	0	TG:AA	
111	738	808	2	0	192	94	?	“stage-specific error” (3 instead of 2 G), GT:TA or TA:AG
**112**	**738**	**809**	**15281**	**22611**	**21705**	**29828**		
114	738	823	41	67	117	76		deletes about 1/5 of the start of exon 5
116	738	837	0	5	3	3		deletes about 2/5 of the start of exon 5
117	738	1024	17	9	30	27		deletes exon 5
118	738	1084	1	0	0	7		deletes exon 5 plus about ¼ of the start of exon 6
119	738	1107	2	0	0	0		
121	740	810	25	23	0	32	AT:GG	
125	742	809	24	21	29	27		adds 4 bases at the end of exon 4
126	742	812	75	151	11	130		“stage-specific error” (5 instead of 4 T at start of exon 5)
130	745	809	0	0	0	3	TT:AG	adds 7 bases at the end of exon 4
131	748	844	0	0	1	0	TG:GG	
133	864	1024	0	9	0	17		deletes about ¼ of exon 5 at the end
134	880	1023	0	1	5	4	AA:CA	
137	881	1024	76	213	3511	5960	AG:AG	“stage-specific error” (2 instead of 3 A)
138	882	1014	0	0	1	0	GT:TT	
**139**	**882**	**1024**	**14773**	**20621**	**20253**	**26043**		
140	882	1032	1	0	0	0	GT:TG	
141	882	1069	1	0	0	1	GT:TT	deletes about 1/6 of exon 6
142	882	1084	2	1	4	3		deletes about 1/5 of exon 6
143	883	1015	0	0	0	1	TT:TA	
145	887	1028	0	1	0	0	AT:TA	
146	892	1216	5	1	0	0	AC:AA	
152	1107	1232	3	0	11	14		creates a new intron inside exon 6, removing about ½ of its sequence
153	1114	1241	0	0	0	6	AA:AA	creates a new intron inside exon 6, removing about ½ of its sequence
154	1120	1618	0	1	0	0	TA:TA	
161	1201	1221	0	1	0	0	AT:GT	
163	1233	1400	2	0	2	0		deletes about ¼ of exon 6
166	1266	1384	1	0	0	0	TA:GA	
171	1290	1400	1	0	0	0		
173	1294	1400	1572	2098	46	58		
176	1296	1400	1	8	15	24	?	dubious alignment, GT:GG or GG:AG
**178**	**1297**	**1400**	**1734**	**2618**	**11191**	**9709**		
179	1297	1602	39	21	34	19		deletes exon 7
199	1448	1602	46	72	48	79		deletes about ½ of exon 7
**205**	**1487**	**1602**	**11050**	**17976**	**14462**	**19195**		
207	1487	1621	0	0	0	1		
212	1654	1813	3	5	1	7		deletes about ½ of exon 8

216	1654	2087	0	0	0	4		deletes about ½ of exon 8 plus the whole of exon 9
223	1702	1783	0	1	0	0	GT:TT	
224	1702	1810	1	6	1	1	GT:TG	
**225**	**1702**	**1813**	**14658**	**23699**	**18392**	**24215**		
227	1702	1844	1	2	0	1		
228	1702	2087	0	0	19	0		
231	1715	1839	0	2	0	0	AA:GA	
233	1891	1960	1	0	0	0		
**236**	**1975**	**2087**	**17118**	**28281**	**20495**	**27086**		
237	1975	2089	4	16	3	10		
238	1975	2237	100	58	56	15		deletes exon 10
239	1995	2087	1	3	0	2		adds 20 bases to the end of exon 9
242	2112	2237	0	0	0	2	AG:AG	
**247**	**2114**	**2237**	**17361**	**28805**	**20705**	**27453**		
248	2114	2243	91	97	65	112		deletes 6 bases from exon 11

Legend

ET	Early trophozoite
LT	Late trophozoite
Start	**start coordinate** for the putative intron involved in the current splice junction
End	**end coordinate** for the putative intron involved in the current splice junction
Ring	number of reads corroborating the current splice junction, as reported by STAR, in the **ring** life cycle stage
Early trophozoite	same as above, but for the **early trophozoite** stage
Late trophozoite	same as above, but for the **late trophozoite** stage
Schizont	same as above, but for the **schizont** stage
Type	type of junction, classified by splice donor and acceptor sequences; when absent, it is the canonical pair (GT:AG); ? when undetermined
Notes	any pertinent notes on the effect, on the final protein, of a particular splice junction variant

Other observations: Rows in **bold typeface** indicate splice junctions that were present in the annotated isoform present in GenBank

Splice junctions whose IDs are in **red background** indicate "stage-specific errors"

Splice junctions whose IDs are in **light blue background** indicate those, excluding previously annotated ones, present in at least one of the 40 newly identified isoforms (see main text)

Additional file 3

Title: Viable isoform splice junction composition

Description: Alignment version of Figure 4, representing the amino acid composition of each predicted viable isoform found. Boldface type distinguishes positions differing between a variant and the annotated isoform, and highlights help visualize in-frame stop codons (red) and length differences (yellow). Alternating black and blue bars above the alignment blocks represent the annotated exon positions along the FPPS/GGPPS, identified by numbers immediately above.

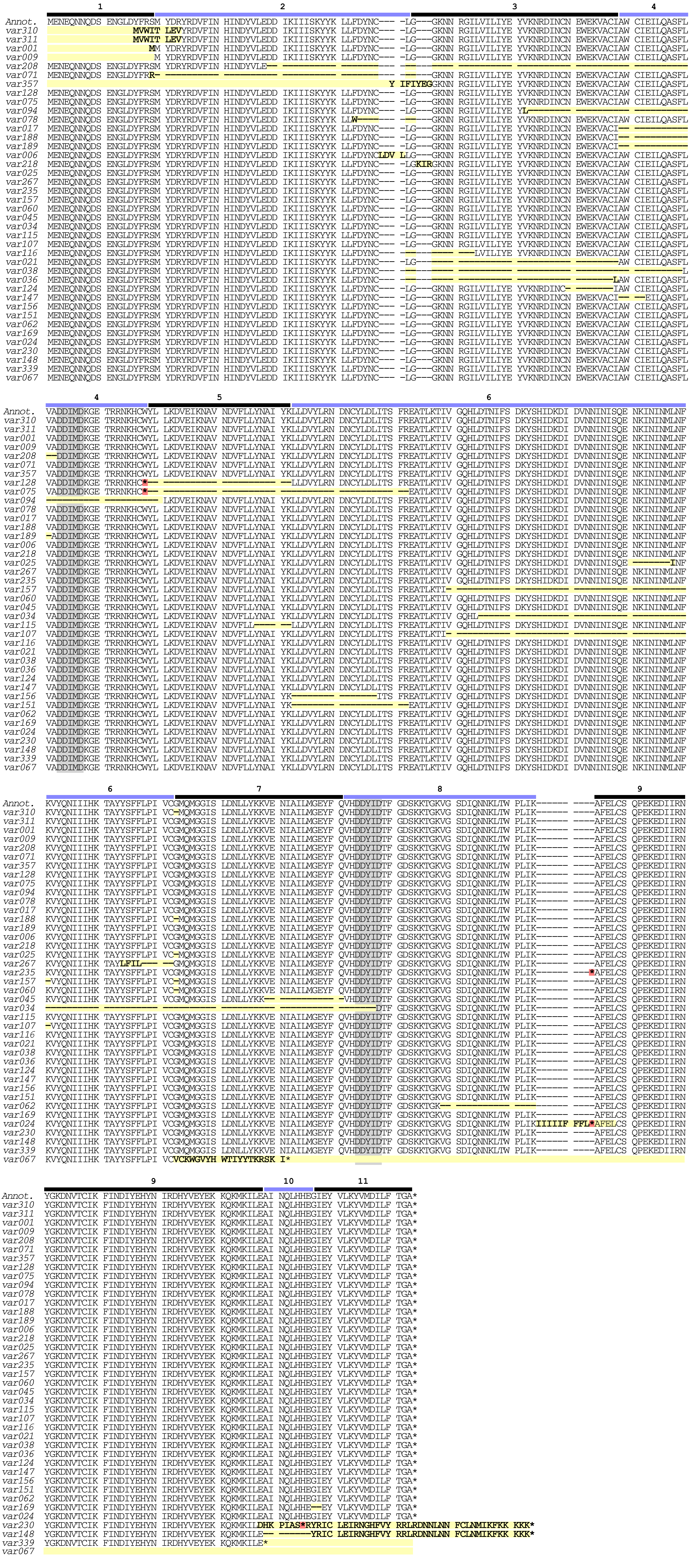
1 2 3 4
Annot. MENEQNNQDS ENGLDYFRSM YDRYRDVFIN HINDYVLEDD IKIIISKYYK LLFDYNC--- -LG---GKNN RGILVILIYE YVKNRDINCN EWEKVACIAW CIEILQASFL
4 5 6
Annot. VADDIMDKGE TRRNKHCWYL LKDVEIKNAV NDVFLLYNAI YKLLDVYLRN DNCYLDLITS FREATLKTIV GQHLDTNIFS DKYSHIDKDI DVNNINISQE NKININMLNF
6 7 8 9
Annot. KVYQNIIIHK TAYYSFFLPI VCGMQMGGIS LDNLLYKKVE NIAILMGEYF QVHDDYIDTF GDSKKTGKVG SDIQNNKLTW PLIK------ ----AFELCS QPEKEDIIRN
9 10 11
Annot. YGKDNVTCIK FINDIYEHYN IRDHYVEYEK KQKMKILEAI NQLHHEGIEY VLKYVMDILF TGA*

Additional file 4

Title: Splice junction combinations detected and their quantifications during the intra-erythrocytic cycle

Description: Named variants represent the viable isoforms presented in Figure 4 and Additional file 4.

Variant	Splice junction combinations	Ring	Early trophozoite	Late trophozoite	Schizont	Total
	2		1	1		2
	5				1	1
	10		1			1
	11		1		1	2
	**12**	2418	7596	2166	4052	16232
	14	13	54	8	24	99
	15		5			5
	19	1	1		1	3
	20		2			2
	25				1	1
var006	52				3	3
	53	1	2			3
	**54**	4	9	10	16	39
	**94**	6	12	1	8	27
	**112**		1			1
	**139**	2	5	2	1	10
	**178**	1	6	10	4	21
	**205**	2	3		2	7
	**225**	70	132	38	113	353
	231		2			2
	**236**	18	37	8	17	80
	238	13	25	3	5	46
	**247**	424	775	314	620	2133
	248	5	4	9	11	29
	103 137 178 205 225 236 247			2	1	3
	109 137 178 205 225 236 247			1		1
	11 54 94 112 139		1	1	1	3
var009	11 54 94 112 139 178			1		1
	111 137 178 205 225				1	1
	112 134 178 205 225 236 247			1	1	2
	112 137 173 205			1		1
	112 137 173 205 225			1	2	3
	112 137 173 205 225 236 247			6	10	16
	112 137 176 205 225 236 247			1	4	5
	112 137 178 205			3	3	6

	112 137 178 205 224 236 247				1	1
	112 137 178 205 225			1	10	11
	112 137 178 205 225 236			2	6	8
	112 137 178 205 225 236 242				1	1
	112 137 178 205 225 236 247			1475	1848	3323
	112 137 178 205 225 237 247				2	2
	112 137 178 205 225 239 247				1	1
	**112 139**	13	9	1	2	25
	112 139 153				6	6
	112 139 163 205 225 236 247			1		1
	112 139 176		1			1
	112 139 176 205		2		1	3
	**112 139 178**	9	11	4	1	25
	112 139 178 199 225 236 247	2	3	3	3	11
	**112 139 178 205**	6	11	5	10	32
	112 139 178 205 216 247				1	1
	**112 139 178 205 225**	15	12	12	27	66
	112 139 178 205 225 236	1		2	4	7
	**112 139 178 205 225 236 247**	37	56	251	342	686
	112 139 178 205 225 236 248	3		4	6	13
var148	112 139 178 205 225 238	1		1	1	3
	112 139 178 205 225 238		1			1
	112 139 178 205 228 247			1		1
	112 139 179	1				1
	112 139 179 225 236 247		1			1
	112 139 179 225 236 247	2		4		6
	112 142 178 205 225 236 247	1				1
	114 137 178 205 225 236 247			7	6	13
	117 178 205 225 236 247		1			1
	117 178 205 225 236 247	1		10	4	15
	117 179 225 236 247				5	5
	118 178 205 225 236 247				1	1
	12 32 54 94	1				1
	12 33 94 112 139 178				1	1
var208	12 34 112 139				1	1
var078	12 35 94		1			1

	12 44	10	12	3	10	35
	12 44 94	13	27	1	10	51
	12 44 94 111			1		1
	12 44 94 112	14	17	4	4	39
	12 44 94 112 137				2	2
	12 44 94 112 139	149	220	7	45	421
	12 44 94 112 139 178	14	11	4	1	30
	12 44 94 112 139 178 205	1		1	1	3
	12 44 94 117	1	1			2
	12 44 96 112 139		3	1		4
	12 47		1	1		2
	12 47 112			1		1
	12 47 112 139	3	1	12		16
var036	12 47 112 139 178		1	4		5
var038	12 48 112 139			1		1
	12 53	198	239	24	16	477
	12 53 83 112 139		1			1
	12 53 92		2			2
	12 53 92 112		3			3
	12 53 94	469	673	36	24	1202
	12 53 94 103 139	5	5			10
	12 53 94 111			1		1
	12 53 94 111 139			6		6
	12 53 94 112	518	965	4	7	1494
	12 53 94 112 133		4			4
	12 53 94 112 137	44	78		3	125
	12 53 94 112 139	6177	5778	136	136	12227
	12 53 94 112 139 163	2				2
	12 53 94 112 139 173	1	2			3
	12 53 94 112 139 178	43	9	56	6	114
	12 53 94 112 142	1				1
	12 53 94 114	11	13			24
	12 53 94 114 139	8	5			13
	12 53 94 117	5	2			7
	12 53 94 119	1				1
	12 53 94 121	6	3			9

	12 53 94 121 139	2	2			4
	12 53 94 125		1			1
	12 53 94 125 139	11	7		1	19
	12 53 94 126	8	10			18
	12 53 94 126 139	30	41			71
	12 53 94 139	2				2
	12 53 95		1			1
	12 53 96		1			1
	12 53 96 112		1			1
	12 53 96 112 139	2	1			3
	12 53 97		1			1
	12 53 97 112 139	1	1			2
	**12 54**	306	587	551	886	2330
	12 54 76 112 139			3		3
	12 54 76 112 139 178			1		1
var094	12 54 77 139			2		2
	12 54 78 112				1	1
var124	12 54 78 112 139				1	1
	12 54 81			1		1
	12 54 81 111			1		1
	12 54 81 111 139			4		4
	12 54 83		2		1	3
	12 54 83 111 139				1	1
	12 54 83 112 139	1				1
	12 54 84 112 139				1	1
	**12 54 94**	521	1297	1590	1559	4967
	12 54 94 103		4	3		7
	12 54 94 103 139	7	11	36	14	68
	12 54 94 104			6		6
	12 54 94 111			24	5	29
	12 54 94 111 139	2		124	56	182
	12 54 94 111 139 173				1	1
	12 54 94 111 139 178			13	7	20
	**12 54 94 112**	580	1892	589	1020	4081
	12 54 94 112 133		4		15	19
	12 54 94 112 134		1		2	3

	12 54 94 112 137	23	118	6	1032	1179
	12 54 94 112 137 152				1	1
	12 54 94 112 137 178		1		2	3
	12 54 94 112 138			1		1
	**12 54 94 112 139**	7127	12732	13680	22453	55992
	12 54 94 112 139 152			1	9	10
	12 54 94 112 139 152 178			2	3	5
var107	12 54 94 112 139 152 178 205			3		3
var060	12 54 94 112 139 173	1	1	19	10	31
var067	12 54 94 112 139 176	1	2	7	6	16
	**12 54 94 112 139 178**	108	78	4096	719	5001
	12 54 94 112 139 178 199			1		1
	**12 54 94 112 139 178 205**			1	3	4
	12 54 94 112 139 179			5		5
	12 54 94 112 140	1				1
var156	12 54 94 112 141				1	1
var151	12 54 94 112 142		1	4	2	7
	12 54 94 112 143				1	1
	12 54 94 112 145		1			1
	12 54 94 114	10	29	20	7	66
	12 54 94 114 133		1			1
	12 54 94 114 137		2			2
	12 54 94 114 139	8	17	71	55	151
	12 54 94 114 139 178	1			3	4
	12 54 94 116		2			2
	12 54 94 116 139		3	3	2	8
	12 54 94 116 139 178				1	1
	12 54 94 117	8	5	17	10	40
	12 54 94 117 178				2	2
	12 54 94 117 178 205			1		1
var128	12 54 94 117 178 205 225			1		1
var075	12 54 94 118	1			6	7
	12 54 94 119	1				1
	12 54 94 121	8	5		7	20
	12 54 94 121 137	1			4	5
	12 54 94 121 139	8	12		20	40

	12 54 94 125	2	2	2		6
	12 54 94 125 137				2	2
	12 54 94 125 139	11	10	21	22	64
	12 54 94 125 139 178			4		4
	12 54 94 126	9	13		8	30
	12 54 94 126 137	1	1		6	8
	12 54 94 126 139	24	78	3	102	207
	12 54 94 126 139 152				1	1
	12 54 94 126 139 173			1		1
	12 54 94 130 139				3	3
	12 54 94 131 139			1		1
	**12 54 94 139**		1			1
	12 54 96	1	1	6	4	12
	12 54 96 111 139			1	16	17
	12 54 96 112			3		3
	12 54 96 112 139	4	5	28	6	43
var147	12 54 96 112 139 178			1		1
	12 54 96 126 139		1			1
	12 54 97		2			2
	12 54 97 112		1			1
	12 54 97 112 139		1			1
var188	12 54 97 112 139 173				1	1
var189	12 54 98 112 139			1	1	2
	12 54 98 114 139				1	1
	12 55		1		1	2
	12 55 94	1	2	1	2	6
	12 55 94 111				1	1
	12 55 94 112	2				2
	12 55 94 112 139	3	2	10		15
	12 55 94 112 139 178			5		5
	12 56		1			1
	12 56 94			1	2	3
	12 56 94 111 139			12		12
	12 56 94 111 139 178			1		1
	12 56 94 112 139	3			2	5
var116	12 56 94 112 139 178	1				1

	12 56 94 114 139			8		8
	12 58 94 112 139			14		14
	12 58 94 112 139 178			8		8
	12 63	3		1	1	5
	12 63 94	4	2	1	3	10
	12 63 94 112		1		3	4
	12 63 94 112 137				5	5
	12 63 94 112 139	10	8	11	29	58
var218	12 63 94 112 139 178		1	4	1	6
	12 69 94 112	1				1
	12 69 94 112 139			6		6
	12 73 139			1		1
	**12 94**				5	5
	**12 94 112**			1		1
	121 139 178				1	1
	125 137 178 205 225 236 247			1	1	2
	126 137 178 205 225 236 247			4	6	10
	126 139 178 205 225		1		1	2
var115	133 178 205 225 236 247				2	2
	134 178 205 225 236 247			2	1	3
	137 173 205 225			1	1	2
	137 173 205 225 236 247			5	14	19
	137 176 205 225 236 247				5	5
	137 178			1		1
	137 178 199 225 236 247			2	3	5
	137 178 205	1	1	1	5	8
	137 178 205 225			4	7	11
	137 178 205 225 236			1	6	7
	137 178 205 225 236 242				1	1
	137 178 205 225 236 247	2	5	1291	1683	2981
	137 178 205 225 238				2	2
	137 178 205 228 247			1		1
var157	139 152 173 205 225	1				1
	139 152 178 205 225 236 247			1		1
	139 171 205	1				1
	139 173 205 225	2	4			6

	139 173 205 225 236	1				1
	139 173 205 225 236 247	26	33			59
	139 176 205 225 236 247		1	2	3	6
	**139 178**	3	2	28	1	34
	139 178 199 225 236 247	6	14	17	14	51
	**139 178 205**	42	45	5	5	97
var062	139 178 205 212 236 247		1			1
	**139 178 205 225**	11	24	18	32	85
	**139 178 205 225 236**	5	7	10	15	37
	**139 178 205 225 236 247**	418	737	794	911	2860
	139 178 205 225 236 248	9	8	4	13	34
	139 178 205 225 238	6	1	10	1	18
	139 178 205 228 247			2		2
	139 179 225 236 247	8	7	12		27
	139 179 225 238			1		1
	14 53 94 112 139				2	2
	14 53 94 112 139 178				1	1
	14 54	6	10	1	9	26
	14 54 94	8	15	3	19	45
	14 54 94 103 139			1		1
	14 54 94 111 139				4	4
	14 54 94 112		1	1		2
	14 54 94 112 139	20	24	37	43	124
var310	14 54 94 112 139 173				2	2
var311	14 54 94 112 139 178		1	9	13	23
	14 54 94 114 139	3				3
	14 54 96 112 139		1			1
	142 178 205 225 236 247				1	1
	146 173 205 225 236 247	5	1			6
	15 44 94 112 139		1			1
	15 54 94 111 139			1		1
	15 69 94 112 139		3			3
	152 178 205 225 236 247			1		1
var025	161 173 205 225 236 247		1			1
	163 205 225 236 247			1		1
var267	166 205 225 236 247	1				1

	17 94 112 139	1		6		7
	173 205 225	14	9	1	1	25
	173 205 225 236	15	20			35
	173 205 225 236 247	1503	2018	11	13	3545
	173 205 225 237 247	1				1
	173 205 225 239 247		2			2
	176 199 225 236 247			1	2	3
	176 205 225		1			1
	176 205 225 236 247		1	2	2	5
	178 199 225		1			1
	178 199 225 236 247	12	13	10	19	54
	**178 205**	23	18	2	20	63
	178 205 212 236 247	3	1	1		5
	178 205 216 247				1	1
var235	178 205 224 236 247		1			1
	**178 205 225**	62	45	21	68	196
	**178 205 225 236**	21	14	7	21	63
	**178 205 225 236 247**	768	1390	2062	2492	6712
	178 205 225 236 248	6	14	7	10	37
	178 205 225 238	29	9	9		47
	178 205 227 236 247	1				1
	178 205 228 247			4		4
	178 207 225 236 247				1	1
	179 225	2	3			5
	179 225 236 247	25	10	11	12	58
	19 53 94 112		1			1
	19 53 94 112 139		1			1
	19 54 94				1	1
	19 54 94 112 137		1		1	2
	199 225			6		6
	199 225 236		1			1
	199 225 236 247	26	39	8	36	109
	2 44		1			1
	2 54 94 112		1			1
var001	2 54 94 112 139		1	7	1	9
	20 53 94	1		1		2

	20 53 94 112		1			1
	20 53 94 112 139	1	2			3
	20 54			2		2
	20 54 94			1		1
	20 54 94 112		1			1
	20 54 94 112 137				1	1
	20 54 94 112 139	3	5	8	7	23
	20 54 94 114 139			3		3
	205 216 247				2	2
	205 224 236 247	1	4	1		6
	**205 225**	28	65	26	52	171
	**205 225 236**	10	26	16	24	76
	**205 225 236 247**	7874	13227	7484	10042	38627
	205 225 236 248	8	10	10	11	39
	205 225 237 247	2	7		5	14
	205 225 238	18	9	13	3	43
	205 225 239 247	1				1
	205 227 236 247		1			1
	205 228 247			1		1
	205 236 247			1	2	3
	212 236 247		3		7	10
var024	223 236 247		1			1
	224 236 247		1			1
	**225 236**	14	16	17	37	84
	**225 236 247**	3453	5519	3801	4807	17580
	225 236 248	44	28	21	34	127
	225 237 247		5	1	1	7
	225 238	32	12	19	3	66
	227 236 247		1		1	2
	228 247			5		5
	**236 247**	2694	4763	2247	3121	12825
	236 248	15	31	7	25	78
var230	237 247	1	4	2	2	9
var339	239 247		1		1	2
var357	30 94 112 139			2		2
	44 94 112	1		1		2

	44 94 112 139				2	2
	44 94 112 139 178	1		1		2
	44 94 112 139 178 199		1			1
	44 94 112 139 178 205	1			1	2
	5 94				1	1
	5 94 112 139		1		1	2
var071	5 94 112 139 178 205				4	4
	53 94	3	4	1	2	10
	53 94 112		4			4
	53 94 112 139	10	27	2	5	44
	53 94 112 139 178			1		1
	53 94 126 139	2	1			3
	**54 94**	15	20	4	23	62
	54 94 103 139	3				3
	**54 94 112**	3	9	9	5	26
	54 94 112 137				3	3
	**54 94 112 139**	34	82	86	128	330
	54 94 112 139 178	1	1	14	12	28
	**54 94 112 139 178 205**	3	4	11	17	35
	**54 94 112 139 178 205 225**		1		1	2
	54 94 112 139 179			1		1
	63 94 112 137				1	1
	63 94 112 139	1				1
	67 94 112 137 178 205 225 236 247			12	21	33
var021	75 112 139			2		2
	94 103 137				1	1
	94 103 137 178 205 225 236 247			3	3	6
	94 111 139			2	1	3
	**94 112**		1	2		3
	94 112 134 178 205 225 236 247			2		2
	94 112 137	2	4		42	48
	94 112 137 176 205 225 236 247				1	1
	94 112 137 176 205 225 236 247			2		2
	94 112 137 178 205	2		1	1	4
	94 112 137 178 205 225			1		1
	94 112 137 178 205 225 236 247			600	1046	1646

	94 112 137 178 205 225 236 248				2	2
	**94 112 139**	190	292	192	427	1101
	94 112 139 152	1				1
	94 112 139 152 178 205 225	1				1
var034	94 112 139 154 225 236 247		1			1
	**94 112 139 178**	13	7	27	10	57
var045	94 112 139 178 199 225 236 247				2	2
	**94 112 139 178 205**	11	4	8	10	33
	**94 112 139 178 205 225**	15	17	9	19	60
	**94 112 139 178 205 225 236 247**			17	20	37
var169	94 112 139 178 205 225 236 248			1		1
	94 112 139 178 205 228 247			5		5
	94 112 139 179	1				1
	94 112 141	1				1
	94 114 137 178 205 225 236 247			1	3	4
	94 114 139			5	1	6
	94 114 139 178 205			1		1
	94 114 139 178 205 225			1		1
	94 117	2				2
	94 117 178 205 225 236 247			1		1
	94 117 179 225 236 247				2	2
	94 121 139		1			1
	94 125 139			1	1	2
	94 126 137		1			1
	94 126 137 178 205 225 236 247			2	4	6
	94 126 139	1	4		2	7
	94 126 139 178 205 225 236 247			1		1
	**94 139 178**			5		5
var017	97 112 139 178 205 225 236 247			1		1

**Variant**	variant name, only present for those variants displayed in **Figure 4** and **Additional file 4**
**Splice junctions**	splice junctions (as named in **Additional file 3**) present in each variant
**Ring**	number of reads in the **ring** life-cycle stage that display a particular combination of splice junctions
**Early trophozoite**	same as above, but for **early trophozoite**
**Late trophozoite**	same as above, but for **late trophozoite**

**Schizont**	same as above, but for **schizont**
**Other observations:**	Splice junction combinations in **bold typeface** are those containing only splice junctions present in the annotated isoform of the gene, present in GenBank

Additional file 5

Title: Viable and intron-retention isoform sequences

Description: Sequences of all viable and intron-retention isoforms identified in this study. Records labeled as "padded" have additional sequence to put transcript fragments into full gene context, while records labeled "original" present only the original sequence. Translated amino acid sequences for the "padded" versions are also included in the file. Comment lines begin with the # character.

>Annot complete annotated isoform of FPPS/GGPPS, with blank spaces marking annotated intron boundaries
ATGGAGAACGAGCAGAATAACCAAGATTCAGAAAATGGTCTGGATTACTTTAGAAGT AT
GTACGATAGATACAGAGATGTGTTCATAAACCATATTAATGATTACGTATTAGAAGATGA
TATAAAAATTATAATTTCAAAATACTATAAACTATTATTTGATTATAACTGCTTAG GGG
GTAAAAATAATAGAGGAATTTTAGTTATATTAATTTATGAGTATGTAAAGAATAGAGATA
TTAATTGTAATGAGTGGGAAAAAGTGGCTTGTATAG CATGGTGTATAGAAATTTTACAA
GCATCTTTTTTAGTAGCAGATGATATTATGGATAAGGGAGAAACACGCAGAAACAAACAT
TGTTG GTATTTGTTAAAAGACGTTGAAATTAAGAATGCGGTGAATGATGTGTTTCTTCT
GTATAACGCTATATACAA ATTACTTGATGTATATTTGCGCAATGATAACTGTTACCTTG
ATTTAATTACATCCTTTAGAGAAGCCACTTTAAAAACTATAGTAGGACAACATTTAGATA
CAAATATATTTTCAGATAAATACTCCCATATAGACAAAGATATAGATGTTAATAATATTA
ATATATCTCAAGAGAATAAAATTAATATAAACATGTTAAATTTTAAGGTTTATCAAAACA
TTATTATTCATAAAACTGCTTATTATTCATTCTTTTTACCTATTGTTTGTG GTATGCAA
ATGGGGGGTATATCATTGGACAATTTATTATACAAAAAGGTCGAAAATATAGCAATTCTT
ATGGGGGAATATTTTCAA GTCCATGATGATTATATAGATACCTTTGGAGATTCTAAAAA
GACGGGAAAAGTTGGCTCAGATATTCAAAATAATAAATTAACGTGGCCCTTGATAAAA G
CATTTGAACTATGTTCACAACCTGAAAAAGAGGACATAATAAGAAATTATGGGAAAGATA
ATGTAACATGTATTAAGTTTATTAATGATATATATGAACATTATAATATCAGGGATCATT
ATGTGGAATATGAAAAGAAGCAGAAGATGAAAATATTAGA AGCCATAAACCAATTGCAT
CATGAAG GTATAGAATATGTCTTGAAATACGTAATGGACATTTTGTTTACAGGCGCTTG
A

>var009_original combination of splice junctions {11 54 94 112 139 178} (see Additional files 3 and 5), with 30 bases flanking the ends
TCAGAAAATGGTCTGGATTACTTTAGAAGT TTCCCCTTCAGATGTACGATAGATACAGA
GATGTGTTCATAAACCATATTAATGATTACGTATTAGAAGATGATATAAAAATTATAATT
TCAAAATACTATAAACTATTATTTGATTATAACTGCTTAG GGGGTAAAAATAATAGAGG
AATTTTAGTTATATTAATTTATGAGTATGTAAAGAATAGAGATATTAATTGTAATGAGTG
GGAAAAAGTGGCTTGTATAG CATGGTGTATAGAAATTTTACAAGCATCTTTTTTAGTAG
CAGATGATATTATGGATAAGGGAGAAACACGCAGAAACAAACATTGTTG GTATTTGTTA
AAAGACGTTGAAATTAAGAATGCGGTGAATGATGTGTTTCTTCTGTATAACGCTATATAC
AA ATTACTTGATGTATATTTGCGCAATGATAACTGTTACCTTGATTTAATTACATCCTT
TAGAGAAGCCACTTTAAAAACTATAGTAGGACAACATTTAGATACAAATATATTTTCAGA
TAAATACTCCCATATAGACAAAGATATAGATGTTAATAATATTAATATATCTCAAGAGAA
TAAAATTAATATAAACATGTTAAATTTTAAGGTTTATCAAAACATTATTATTCATAAAAC
TGCTTATTATTCATTCTTTTTACCTATTGTTTGTG GTATGCAAATGGGGGGTATATCAT
TGGACA

>var148_original combination of splice junctions {112 139 178 205 225 238} (see Additional files 3 and 5), with 30 bases flanking the ends
GGGAGAAACACGCAGAAACAAACATTGTTG GTATTTGTTAAAAGACGTTGAAATTAAGA
ATGCGGTGAATGATGTGTTTCTTCTGTATAACGCTATATACAA ATTACTTGATGTATAT
TTGCGCAATGATAACTGTTACCTTGATTTAATTACATCCTTTAGAGAAGCCACTTTAAAA
ACTATAGTAGGACAACATTTAGATACAAATATATTTTCAGATAAATACTCCCATATAGAC
AAAGATATAGATGTTAATAATATTAATATATCTCAAGAGAATAAAATTAATATAAACATG
TTAAATTTTAAGGTTTATCAAAACATTATTATTCATAAAACTGCTTATTATTCATTCTTT
TTACCTATTGTTTGTG GTATGCAAATGGGGGGTATATCATTGGACAATTTATTATACAA
AAAGGTCGAAAATATAGCAATTCTTATGGGGGAATATTTTCAA GTCCATGATGATTATA
TAGATACCTTTGGAGATTCTAAAAAGACGGGAAAAGTTGGCTCAGATATTCAAAATAATA
AATTAACGTGGCCCTTGATAAAA GCATTTGAACTATGTTCACAACCTGAAAAAGAGGAC
ATAATAAGAAATTATGGGAAAGATAATGTAACATGTATTAAGTTTATTAATGATATATAT
GAACATTATAATATCAGGGATCATTATGTGGAATATGAAAAGAAGCAGAAGATGAAAATA
TTAGA GTATAGAATATGTCTTGAAATACGTAATGG

>var208_original combination of splice junctions {12 34 112 139} (see Additional files 3 and 5), with 30 bases flanking the ends
TCAGAAAATGGTCTGGATTACTTTAGAAGT ATGTACGATAGATACAGAGATGTGTTCAT
AAACCATATTAATGATTACGTATTAGA AGATGATATTATGGATAAGGGAGAAACACGCA
GAAACAAACATTGTTG GTATTTGTTAAAAGACGTTGAAATTAAGAATGCGGTGAATGAT
GTGTTTCTTCTGTATAACGCTATATACAA ATTACTTGATGTATATTTGCGCAATGATAA

>var078_original combination of splice junctions {12 35 94} (see Additional files 3 and 5), with 30 bases flanking the ends

TCAGAAAATGGTCTGGATTACTTTAGAAGT ATGTACGATAGATACAGAGATGTGTTCAT
AAACCATATTAATGATTACGTATTAGAAGATGATATAAAAATTATAATTTCAAAATACTA
TAAACTATTAT GGGGTAAAAATAATAGAGGAATTTTAGTTATATTAATTTATGAGTATG
TAAAGAATAGAGATATTAATTGTAATGAGTGGGAAAAAGTGGCTTGTATAG CATGGTGT
ATAGAAATTTTACAAGCATCTT

>var036_original combination of splice junctions {12 47 112 139 178} (see Additional files 3 and 5), with 30 bases flanking the ends
TCAGAAAATGGTCTGGATTACTTTAGAAGT ATGTACGATAGATACAGAGATGTGTTCAT
AAACCATATTAATGATTACGTATTAGAAGATGATATAAAAATTATAATTTCAAAATACTA
TAAACTATTATTTGATTATAACTGCTTAG CATGGTGTATAGAAATTTTACAAGCATCTT
TTTTAGTAGCAGATGATATTATGGATAAGGGAGAAACACGCAGAAACAAACATTGTTG G
TATTTGTTAAAAGACGTTGAAATTAAGAATGCGGTGAATGATGTGTTTCTTCTGTATAAC
GCTATATACAA ATTACTTGATGTATATTTGCGCAATGATAACTGTTACCTTGATTTAAT
TACATCCTTTAGAGAAGCCACTTTAAAAACTATAGTAGGACAACATTTAGATACAAATAT
ATTTTCAGATAAATACTCCCATATAGACAAAGATATAGATGTTAATAATATTAATATATC
TCAAGAGAATAAAATTAATATAAACATGTTAAATTTTAAGGTTTATCAAAACATTATTAT
TCATAAAACTGCTTATTATTCATTCTTTTTACCTATTGTTTGTG GTATGCAAATGGGGG
GTATATCATTGGACA

>var038_original combination of splice junctions {12 48 112 139} (see Additional files 3 and 5), with 30 bases flanking the ends
TCAGAAAATGGTCTGGATTACTTTAGAAGT ATGTACGATAGATACAGAGATGTGTTCAT
AAACCATATTAATGATTACGTATTAGAAGATGATATAAAAATTATAATTTCAAAATACTA
TAAACTATTATTTGATTATAACTGCTTAG TAGCAGATGATATTATGGATAAGGGAGAAA
CACGCAGAAACAAACATTGTTG GTATTTGTTAAAAGACGTTGAAATTAAGAATGCGGTG
AATGATGTGTTTCTTCTGTATAACGCTATATACAA ATTACTTGATGTATATTTGCGCAA
TGATAA

>var094_original combination of splice junctions {12 54 77 139} (see Additional files 3 and 5), with 30 bases flanking the ends
TCAGAAAATGGTCTGGATTACTTTAGAAGT ATGTACGATAGATACAGAGATGTGTTCAT
AAACCATATTAATGATTACGTATTAGAAGATGATATAAAAATTATAATTTCAAAATACTA
TAAACTATTATTTGATTATAACTGCTTAG GGGGTAAAAATAATAGAGGAATTTTAGTTA
TATTAATTTATGA GTATTTGTTAAAAGACGTTGAAATTAAGAATGCGGTGAATGATGTG
TTTCTTCTGTATAACGCTATATACAA ATTACTTGATGTATATTTGCGCAATGATAA

>var124_original combination of splice junctions {12 54 78 112 139} (see Additional files 3 and 5), with 30 bases flanking the ends
TCAGAAAATGGTCTGGATTACTTTAGAAGT ATGTACGATAGATACAGAGATGTGTTCAT
AAACCATATTAATGATTACGTATTAGAAGATGATATAAAAATTATAATTTCAAAATACTA
TAAACTATTATTTGATTATAACTGCTTAG GGGGTAAAAATAATAGAGGAATTTTAGTTA
TATTAATTTATGAGTATGTAAAGAATAGAGATATTAATTGTA TAGCATGGTGTATAGAA
ATTTTACAAGCATCTTTTTTAGTAGCAGATGATATTATGGATAAGGGAGAAACACGCAGA
AACAAACATTGTTG GTATTTGTTAAAAGACGTTGAAATTAAGAATGCGGTGAATGATGT
GTTTCTTCTGTATAACGCTATATACAA ATTACTTGATGTATATTTGCGCAATGATAA

>var107_original combination of splice junctions {12 54 94 112 139 152 178 205} (see Additional files 3 and 5), with 30 bases flanking the ends
TCAGAAAATGGTCTGGATTACTTTAGAAGT ATGTACGATAGATACAGAGATGTGTTCAT
AAACCATATTAATGATTACGTATTAGAAGATGATATAAAAATTATAATTTCAAAATACTA
TAAACTATTATTTGATTATAACTGCTTAG GGGGTAAAAATAATAGAGGAATTTTAGTTA
TATTAATTTATGAGTATGTAAAGAATAGAGATATTAATTGTAATGAGTGGGAAAAAGTGG
CTTGTATAG CATGGTGTATAGAAATTTTACAAGCATCTTTTTTAGTAGCAGATGATATT
ATGGATAAGGGAGAAACACGCAGAAACAAACATTGTTG GTATTTGTTAAAAGACGTTGA
AATTAAGAATGCGGTGAATGATGTGTTTCTTCTGTATAACGCTATATACAA ATTACTTG
ATGTATATTTGCGCAATGATAACTGTTACCTTGATTTAATTACATCCTTTAGAGAAGCCA
CTTTAAAAACTATA GTTTATCAAAACATTATTATTCATAAAACTGCTTATTATTCATTC
TTTTTACCTATTGTTTGTG GTATGCAAATGGGGGGTATATCATTGGACAATTTATTATA
CAAAAAGGTCGAAAATATAGCAATTCTTATGGGGGAATATTTTCAA GTCCATGATGATT
ATATAGATACCTTTGGA

>var060_original combination of splice junctions {12 54 94 112 139 173} (see Additional files 3 and 5), with 30 bases flanking the ends

TCAGAAAATGGTCTGGATTACTTTAGAAGT ATGTACGATAGATACAGAGATGTGTTCAT
AAACCATATTAATGATTACGTATTAGAAGATGATATAAAAATTATAATTTCAAAATACTA
TAAACTATTATTTGATTATAACTGCTTAG GGGGTAAAAATAATAGAGGAATTTTAGTTA
TATTAATTTATGAGTATGTAAAGAATAGAGATATTAATTGTAATGAGTGGGAAAAAGTGG
CTTGTATAG CATGGTGTATAGAAATTTTACAAGCATCTTTTTTAGTAGCAGATGATATT
ATGGATAAGGGAGAAACACGCAGAAACAAACATTGTTG GTATTTGTTAAAAGACGTTGA
AATTAAGAATGCGGTGAATGATGTGTTTCTTCTGTATAACGCTATATACAA ATTACTTG
ATGTATATTTGCGCAATGATAACTGTTACCTTGATTTAATTACATCCTTTAGAGAAGCCA
CTTTAAAAACTATAGTAGGACAACATTTAGATACAAATATATTTTCAGATAAATACTCCC
ATATAGACAAAGATATAGATGTTAATAATATTAATATATCTCAAGAGAATAAAATTAATA
TAAACATGTTAAATTTTAAGGTTTATCAAAACATTATTATTCATAAAACTGCTTATTATT
CATTCTTTTTACCTATTGTTT GTATGCAAATGGGGGGTATATCATTGGACA

>var067_original combination of splice junctions {12 54 94 112 139 176} (see Additional files 3 and 5), with 30 bases flanking the ends
TCAGAAAATGGTCTGGATTACTTTAGAAGT ATGTACGATAGATACAGAGATGTGTTCAT
AAACCATATTAATGATTACGTATTAGAAGATGATATAAAAATTATAATTTCAAAATACTA
TAAACTATTATTTGATTATAACTGCTTAG GGGGTAAAAATAATAGAGGAATTTTAGTTA
TATTAATTTATGAGTATGTAAAGAATAGAGATATTAATTGTAATGAGTGGGAAAAAGTGG
CTTGTATAG CATGGTGTATAGAAATTTTACAAGCATCTTTTTTAGTAGCAGATGATATT
ATGGATAAGGGAGAAACACGCAGAAACAAACATTGTTG GTATTTGTTAAAAGACGTTGA
AATTAAGAATGCGGTGAATGATGTGTTTCTTCTGTATAACGCTATATACAA ATTACTTG
ATGTATATTTGCGCAATGATAACTGTTACCTTGATTTAATTACATCCTTTAGAGAAGCCA
CTTTAAAAACTATAGTAGGACAACATTTAGATACAAATATATTTTCAGATAAATACTCCC
ATATAGACAAAGATATAGATGTTAATAATATTAATATATCTCAAGAGAATAAAATTAATA
TAAACATGTTAAATTTTAAGGTTTATCAAAACATTATTATTCATAAAACTGCTTATTATT
CATTCTTTTTACCTATTGTTTGT GTATGCAAATGGGGGGTATATCATTGGACA

>var156_original combination of splice junctions {12 54 94 112 141} (see Additional files 3 and 5), with 30 bases flanking the ends
TCAGAAAATGGTCTGGATTACTTTAGAAGT ATGTACGATAGATACAGAGATGTGTTCAT
AAACCATATTAATGATTACGTATTAGAAGATGATATAAAAATTATAATTTCAAAATACTA
TAAACTATTATTTGATTATAACTGCTTAG GGGGTAAAAATAATAGAGGAATTTTAGTTA
TATTAATTTATGAGTATGTAAAGAATAGAGATATTAATTGTAATGAGTGGGAAAAAGTGG
CTTGTATAG CATGGTGTATAGAAATTTTACAAGCATCTTTTTTAGTAGCAGATGATATT
ATGGATAAGGGAGAAACACGCAGAAACAAACATTGTTG GTATTTGTTAAAAGACGTTGA
AATTAAGAATGCGGTGAATGATGTGTTTCTTCTGTATAACGCTATATACAA AATTACAT
CCTTTAGAGAAGCCACTTTAAA

>var151_original combination of splice junctions {12 54 94 112 142} (see Additional files 3 and 5), with 30 bases flanking the ends
TCAGAAAATGGTCTGGATTACTTTAGAAGT ATGTACGATAGATACAGAGATGTGTTCAT
AAACCATATTAATGATTACGTATTAGAAGATGATATAAAAATTATAATTTCAAAATACTA
TAAACTATTATTTGATTATAACTGCTTAG GGGGTAAAAATAATAGAGGAATTTTAGTTA
TATTAATTTATGAGTATGTAAAGAATAGAGATATTAATTGTAATGAGTGGGAAAAAGTGG
CTTGTATAG CATGGTGTATAGAAATTTTACAAGCATCTTTTTTAGTAGCAGATGATATT
ATGGATAAGGGAGAAACACGCAGAAACAAACATTGTTG GTATTTGTTAAAAGACGTTGA
AATTAAGAATGCGGTGAATGATGTGTTTCTTCTGTATAACGCTATATACAA AGAAGCCA
CTTTAAAAACTATAGTAGGACA

>var128_original combination of splice junctions {12 54 94 117 178 205 225} (see Additional files 3 and 5), with 30 bases flanking the ends
TCAGAAAATGGTCTGGATTACTTTAGAAGT ATGTACGATAGATACAGAGATGTGTTCAT
AAACCATATTAATGATTACGTATTAGAAGATGATATAAAAATTATAATTTCAAAATACTA
TAAACTATTATTTGATTATAACTGCTTAG GGGGTAAAAATAATAGAGGAATTTTAGTTA
TATTAATTTATGAGTATGTAAAGAATAGAGATATTAATTGTAATGAGTGGGAAAAAGTGG
CTTGTATAG CATGGTGTATAGAAATTTTACAAGCATCTTTTTTAGTAGCAGATGATATT
ATGGATAAGGGAGAAACACGCAGAAACAAACATTGTTG ATTACTTGATGTATATTTGCG
CAATGATAACTGTTACCTTGATTTAATTACATCCTTTAGAGAAGCCACTTTAAAAACTAT
AGTAGGACAACATTTAGATACAAATATATTTTCAGATAAATACTCCCATATAGACAAAGA
TATAGATGTTAATAATATTAATATATCTCAAGAGAATAAAATTAATATAAACATGTTAAA
TTTTAAGGTTTATCAAAACATTATTATTCATAAAACTGCTTATTATTCATTCTTTTTACC
TATTGTTTGTG GTATGCAAATGGGGGGTATATCATTGGACAATTTATTATACAAAAAGG
TCGAAAATATAGCAATTCTTATGGGGGAATATTTTCAA GTCCATGATGATTATATAGAT

ACCTTTGGAGATTCTAAAAAGACGGGAAAAGTTGGCTCAGATATTCAAAATAATAAATTA
ACGTGGCCCTTGATAAAA GCATTTGAACTATGTTCACAACCTGAAAAA

>var075_original combination of splice junctions {12 54 94 118} (see Additional files 3 and 5), with 30 bases flanking the ends
TCAGAAAATGGTCTGGATTACTTTAGAAGT ATGTACGATAGATACAGAGATGTGTTCAT
AAACCATATTAATGATTACGTATTAGAAGATGATATAAAAATTATAATTTCAAAATACTA
TAAACTATTATTTGATTATAACTGCTTAG GGGGTAAAAATAATAGAGGAATTTTAGTTA
TATTAATTTATGAGTATGTAAAGAATAGAGATATTAATTGTAATGAGTGGGAAAAAGTGG
CTTGTATAG CATGGTGTATAGAAATTTTACAAGCATCTTTTTTAGTAGCAGATGATATT
ATGGATAAGGGAGAAACACGCAGAAACAAACATTGTTG AGAAGCCACTTTAAAAACTAT
AGTAGGACA

>var147_original combination of splice junctions {12 54 96 112 139 178} (see Additional files 3 and 5), with 30 bases flanking the ends
TCAGAAAATGGTCTGGATTACTTTAGAAGT ATGTACGATAGATACAGAGATGTGTTCAT
AAACCATATTAATGATTACGTATTAGAAGATGATATAAAAATTATAATTTCAAAATACTA
TAAACTATTATTTGATTATAACTGCTTAG GGGGTAAAAATAATAGAGGAATTTTAGTTA
TATTAATTTATGAGTATGTAAAGAATAGAGATATTAATTGTAATGAGTGGGAAAAAGTGG
CTTGTATAG AAATTTTACAAGCATCTTTTTTAGTAGCAGATGATATTATGGATAAGGGA
GAAACACGCAGAAACAAACATTGTTG GTATTTGTTAAAAGACGTTGAAATTAAGAATGC
GGTGAATGATGTGTTTCTTCTGTATAACGCTATATACAA ATTACTTGATGTATATTTGC
GCAATGATAACTGTTACCTTGATTTAATTACATCCTTTAGAGAAGCCACTTTAAAAACTA
TAGTAGGACAACATTTAGATACAAATATATTTTCAGATAAATACTCCCATATAGACAAAG
ATATAGATGTTAATAATATTAATATATCTCAAGAGAATAAAATTAATATAAACATGTTAA
ATTTTAAGGTTTATCAAAACATTATTATTCATAAAACTGCTTATTATTCATTCTTTTTAC
CTATTGTTTGTG GTATGCAAATGGGGGGTATATCATTGGACA

>var188_original combination of splice junctions {12 54 97 112 139 173} (see Additional files 3 and 5), with 30 bases flanking the ends
TCAGAAAATGGTCTGGATTACTTTAGAAGT ATGTACGATAGATACAGAGATGTGTTCAT
AAACCATATTAATGATTACGTATTAGAAGATGATATAAAAATTATAATTTCAAAATACTA
TAAACTATTATTTGATTATAACTGCTTAG GGGGTAAAAATAATAGAGGAATTTTAGTTA
TATTAATTTATGAGTATGTAAAGAATAGAGATATTAATTGTAATGAGTGGGAAAAAGTGG
CTTGTATAG TAGCAGATGATATTATGGATAAGGGAGAAACACGCAGAAACAAACATTGT
TG GTATTTGTTAAAAGACGTTGAAATTAAGAATGCGGTGAATGATGTGTTTCTTCTGTA
TAACGCTATATACAA ATTACTTGATGTATATTTGCGCAATGATAACTGTTACCTTGATT
TAATTACATCCTTTAGAGAAGCCACTTTAAAAACTATAGTAGGACAACATTTAGATACAA
ATATATTTTCAGATAAATACTCCCATATAGACAAAGATATAGATGTTAATAATATTAATA
TATCTCAAGAGAATAAAATTAATATAAACATGTTAAATTTTAAGGTTTATCAAAACATTA
TTATTCATAAAACTGCTTATTATTCATTCTTTTTACCTATTGTTT GTATGCAAATGGGG
GGTATATCATTGGACA

>var189_original combination of splice junctions {12 54 98 112 139} (see Additional files 3 and 5), with 30 bases flanking the ends
TCAGAAAATGGTCTGGATTACTTTAGAAGT ATGTACGATAGATACAGAGATGTGTTCAT
AAACCATATTAATGATTACGTATTAGAAGATGATATAAAAATTATAATTTCAAAATACTA
TAAACTATTATTTGATTATAACTGCTTAG GGGGTAAAAATAATAGAGGAATTTTAGTTA
TATTAATTTATGAGTATGTAAAGAATAGAGATATTAATTGTAATGAGTGGGAAAAAGTGG
CTTGTATAG CAGATGATATTATGGATAAGGGAGAAACACGCAGAAACAAACATTGTTG
GTATTTGTTAAAAGACGTTGAAATTAAGAATGCGGTGAATGATGTGTTTCTTCTGTATAA
CGCTATATACAA ATTACTTGATGTATATTTGCGCAATGATAA

>var218_original combination of splice junctions {12 63 94 112 139 178} (see Additional files 3 and 5), with 30 bases flanking the ends
TCAGAAAATGGTCTGGATTACTTTAGAAGT ATGTACGATAGATACAGAGATGTGTTCAT
AAACCATATTAATGATTACGTATTAGAAGATGATATAAAAATTATAATTTCAAAATACTA
TAAACTATTATTTGATTATAACTGCTTAGGTAAGATAA GGGGTAAAAATAATAGAGGAA
TTTTAGTTATATTAATTTATGAGTATGTAAAGAATAGAGATATTAATTGTAATGAGTGGG
AAAAAGTGGCTTGTATAG CATGGTGTATAGAAATTTTACAAGCATCTTTTTTAGTAGCA
GATGATATTATGGATAAGGGAGAAACACGCAGAAACAAACATTGTTG GTATTTGTTAAA
AGACGTTGAAATTAAGAATGCGGTGAATGATGTGTTTCTTCTGTATAACGCTATATACAA
 ATTACTTGATGTATATTTGCGCAATGATAACTGTTACCTTGATTTAATTACATCCTTTA
GAGAAGCCACTTTAAAAACTATAGTAGGACAACATTTAGATACAAATATATTTTCAGATA

AATACTCCCATATAGACAAAGATATAGATGTTAATAATATTAATATATCTCAAGAGAATA
AAATTAATATAAACATGTTAAATTTTAAGGTTTATCAAAACATTATTATTCATAAAACTG
CTTATTATTCATTCTTTTTACCTATTGTTTGTG GTATGCAAATGGGGGGTATATCATTG
GACA

>var115_original combination of splice junctions {133 178 205 225 236 247} (see Additional files 3 and 5), with 30 bases flanking the ends
TAAGAATGCGGTGAATGATGTGTTTCTTCT ATTACTTGATGTATATTTGCGCAATGATA
ACTGTTACCTTGATTTAATTACATCCTTTAGAGAAGCCACTTTAAAAACTATAGTAGGAC
AACATTTAGATACAAATATATTTTCAGATAAATACTCCCATATAGACAAAGATATAGATG
TTAATAATATTAATATATCTCAAGAGAATAAAATTAATATAAACATGTTAAATTTTAAGG
TTTATCAAAACATTATTATTCATAAAACTGCTTATTATTCATTCTTTTTACCTATTGTTT
GTG GTATGCAAATGGGGGGTATATCATTGGACAATTTATTATACAAAAAGGTCGAAAAT
ATAGCAATTCTTATGGGGGAATATTTTCAA GTCCATGATGATTATATAGATACCTTTGG
AGATTCTAAAAAGACGGGAAAAGTTGGCTCAGATATTCAAAATAATAAATTAACGTGGCC
CTTGATAAAA GCATTTGAACTATGTTCACAACCTGAAAAAGAGGACATAATAAGAAATT
ATGGGAAAGATAATGTAACATGTATTAAGTTTATTAATGATATATATGAACATTATAATA
TCAGGGATCATTATGTGGAATATGAAAAGAAGCAGAAGATGAAAATATTAGA AGCCATA
AACCAATTGCATCATGAAG GTATAGAATATGTCTTGAAATACGTAATGG

>var116_original combination of splice junctions {12 56 94 112 139 178} (see Additional files 3 and 5), with 30 bases flanking the ends
TCAGAAAATGGTCTGGATTACTTTAGAAGT ATGTACGATAGATACAGAGATGTGTTCAT
AAACCATATTAATGATTACGTATTAGAAGATGATATAAAAATTATAATTTCAAAATACTA
TAAACTATTATTTGATTATAACTGCTTAG TTATATTAATTTATGAGTATGTAAAGAATA
GAGATATTAATTGTAATGAGTGGGAAAAAGTGGCTTGTATAG CATGGTGTATAGAAATT
TTACAAGCATCTTTTTTAGTAGCAGATGATATTATGGATAAGGGAGAAACACGCAGAAAC
AAACATTGTTG GTATTTGTTAAAAGACGTTGAAATTAAGAATGCGGTGAATGATGTGTT
TCTTCTGTATAACGCTATATACAA ATTACTTGATGTATATTTGCGCAATGATAACTGTT
ACCTTGATTTAATTACATCCTTTAGAGAAGCCACTTTAAAAACTATAGTAGGACAACATT
TAGATACAAATATATTTTCAGATAAATACTCCCATATAGACAAAGATATAGATGTTAATA
ATATTAATATATCTCAAGAGAATAAAATTAATATAAACATGTTAAATTTTAAGGTTTATC
AAAACATTATTATTCATAAAACTGCTTATTATTCATTCTTTTTACCTATTGTTTGTG GT
ATGCAAATGGGGGGTATATCATTGGACA

>var157_original combination of splice junctions {139 152 173 205 225} (see Additional files 3 and 5), with 30 bases flanking the ends
TGTGTTTCTTCTGTATAACGCTATATACAA ATTACTTGATGTATATTTGCGCAATGATA
ACTGTTACCTTGATTTAATTACATCCTTTAGAGAAGCCACTTTAAAAACTATA GTTTAT
CAAAACATTATTATTCATAAAACTGCTTATTATTCATTCTTTTTACCTATTGTTT GTAT
GCAAATGGGGGGTATATCATTGGACAATTTATTATACAAAAAGGTCGAAAATATAGCAAT
TCTTATGGGGGAATATTTTCAA GTCCATGATGATTATATAGATACCTTTGGAGATTCTA
AAAAGACGGGAAAAGTTGGCTCAGATATTCAAAATAATAAATTAACGTGGCCCTTGATAA
AA GCATTTGAACTATGTTCACAACCTGAAAAA

>var062_original combination of splice junctions {139 178 205 212 236 247} (see Additional files 3 and 5), with 30 bases flanking the ends
TGTGTTTCTTCTGTATAACGCTATATACAA ATTACTTGATGTATATTTGCGCAATGATA
ACTGTTACCTTGATTTAATTACATCCTTTAGAGAAGCCACTTTAAAAACTATAGTAGGAC
AACATTTAGATACAAATATATTTTCAGATAAATACTCCCATATAGACAAAGATATAGATG
TTAATAATATTAATATATCTCAAGAGAATAAAATTAATATAAACATGTTAAATTTTAAGG
TTTATCAAAACATTATTATTCATAAAACTGCTTATTATTCATTCTTTTTACCTATTGTTT
GTG GTATGCAAATGGGGGGTATATCATTGGACAATTTATTATACAAAAAGGTCGAAAAT
ATAGCAATTCTTATGGGGGAATATTTTCAA GTCCATGATGATTATATAGATACCTTTGG
AGATTCTAAAAAGACGGGAAAA GCATTTGAACTATGTTCACAACCTGAAAAAGAGGACA
TAATAAGAAATTATGGGAAAGATAATGTAACATGTATTAAGTTTATTAATGATATATATG
AACATTATAATATCAGGGATCATTATGTGGAATATGAAAAGAAGCAGAAGATGAAAATAT
TAGA AGCCATAAACCAATTGCATCATGAAG GTATAGAATATGTCTTGAAATACGTAAT
GG

>var310_original combination of splice junctions {14 54 94 112 139 173} (see Additional files 3 and 5), with 30 bases flanking the ends
TCAGAAAATGGTCTGGATTACTTTAGAAGT ATACAGAGATGTGTTCATAAACCATATTA
ATGATTACGTATTAGAAGATGATATAAAAATTATAATTTCAAAATACTATAAACTATTAT

TTGATTATAACTGCTTAG GGGGTAAAAATAATAGAGGAATTTTAGTTATATTAATTTAT
GAGTATGTAAAGAATAGAGATATTAATTGTAATGAGTGGGAAAAAGTGGCTTGTATAG C
ATGGTGTATAGAAATTTTACAAGCATCTTTTTTAGTAGCAGATGATATTATGGATAAGGG
AGAAACACGCAGAAACAAACATTGTTG GTATTTGTTAAAAGACGTTGAAATTAAGAATG
CGGTGAATGATGTGTTTCTTCTGTATAACGCTATATACAA ATTACTTGATGTATATTTG
CGCAATGATAACTGTTACCTTGATTTAATTACATCCTTTAGAGAAGCCACTTTAAAAACT
ATAGTAGGACAACATTTAGATACAAATATATTTTCAGATAAATACTCCCATATAGACAAA
GATATAGATGTTAATAATATTAATATATCTCAAGAGAATAAAATTAATATAAACATGTTA
AATTTTAAGGTTTATCAAAACATTATTATTCATAAAACTGCTTATTATTCATTCTTTTTA
CCTATTGTTT GTATGCAAATGGGGGGTATATCATTGGACA

>var311_original combination of splice junctions {14 54 94 112 139 178} (see Additional files 3 and 5), with 30 bases flanking the ends
TCAGAAAATGGTCTGGATTACTTTAGAAGT ATACAGAGATGTGTTCATAAACCATATTA
ATGATTACGTATTAGAAGATGATATAAAAATTATAATTTCAAAATACTATAAACTATTAT
TTGATTATAACTGCTTAG GGGGTAAAAATAATAGAGGAATTTTAGTTATATTAATTTAT
GAGTATGTAAAGAATAGAGATATTAATTGTAATGAGTGGGAAAAAGTGGCTTGTATAG C
ATGGTGTATAGAAATTTTACAAGCATCTTTTTTAGTAGCAGATGATATTATGGATAAGGG
AGAAACACGCAGAAACAAACATTGTTG GTATTTGTTAAAAGACGTTGAAATTAAGAATG
CGGTGAATGATGTGTTTCTTCTGTATAACGCTATATACAA ATTACTTGATGTATATTTG
CGCAATGATAACTGTTACCTTGATTTAATTACATCCTTTAGAGAAGCCACTTTAAAAACT
ATAGTAGGACAACATTTAGATACAAATATATTTTCAGATAAATACTCCCATATAGACAAA
GATATAGATGTTAATAATATTAATATATCTCAAGAGAATAAAATTAATATAAACATGTTA
AATTTTAAGGTTTATCAAAACATTATTATTCATAAAACTGCTTATTATTCATTCTTTTTA
CCTATTGTTTGTG GTATGCAAATGGGGGGTATATCATTGGACA

>var025_original combination of splice junctions {161 173 205 225 236 247} (see Additional files 3 and 5), with 30 bases flanking the ends
ATGTTAATAATATTAATATATCTCAAGAGA TAAATTTTAAGGTTTATCAAAACATTATT
ATTCATAAAACTGCTTATTATTCATTCTTTTTACCTATTGTTT GTATGCAAATGGGGGG
TATATCATTGGACAATTTATTATACAAAAAGGTCGAAAATATAGCAATTCTTATGGGGGA
ATATTTTCAA GTCCATGATGATTATATAGATACCTTTGGAGATTCTAAAAAGACGGGAA
AAGTTGGCTCAGATATTCAAAATAATAAATTAACGTGGCCCTTGATAAAA GCATTTGAA
CTATGTTCACAACCTGAAAAAGAGGACATAATAAGAAATTATGGGAAAGATAATGTAACA
TGTATTAAGTTTATTAATGATATATATGAACATTATAATATCAGGGATCATTATGTGGAA
TATGAAAAGAAGCAGAAGATGAAAATATTAGA AGCCATAAACCAATTGCATCATGAAG
GTATAGAATATGTCTTGAAATACGTAATGG

>var267_original combination of splice junctions {166 205 225 236 247} (see Additional files 3 and 5), with 30 bases flanking the ends
TATCAAAACATTATTATTCATAAAACTGCT TATTTATTCATTTTAGGTATGCAAATGGG
GGGTATATCATTGGACAATTTATTATACAAAAAGGTCGAAAATATAGCAATTCTTATGGG
GGAATATTTTCAA GTCCATGATGATTATATAGATACCTTTGGAGATTCTAAAAAGACGG
GAAAAGTTGGCTCAGATATTCAAAATAATAAATTAACGTGGCCCTTGATAAAA GCATTT
GAACTATGTTCACAACCTGAAAAAGAGGACATAATAAGAAATTATGGGAAAGATAATGTA
ACATGTATTAAGTTTATTAATGATATATATGAACATTATAATATCAGGGATCATTATGTG
GAATATGAAAAGAAGCAGAAGATGAAAATATTAGA AGCCATAAACCAATTGCATCATGA
AG GTATAGAATATGTCTTGAAATACGTAATGG

>var235_original combination of splice junctions {178 205 224 236 247} (see Additional files 3 and 5), with 30 bases flanking the ends
ATTATTCATTCTTTTTACCTATTGTTTGTG GTATGCAAATGGGGGGTATATCATTGGAC
AATTTATTATACAAAAAGGTCGAAAATATAGCAATTCTTATGGGGGAATATTTTCAA GT
CCATGATGATTATATAGATACCTTTGGAGATTCTAAAAAGACGGGAAAAGTTGGCTCAGA
TATTCAAAATAATAAATTAACGTGGCCCTTGATAAAA TAGGCATTTGAACTATGTTCAC
AACCTGAAAAAGAGGACATAATAAGAAATTATGGGAAAGATAATGTAACATGTATTAAGT
TTATTAATGATATATATGAACATTATAATATCAGGGATCATTATGTGGAATATGAAAAGA
AGCAGAAGATGAAAATATTAGA AGCCATAAACCAATTGCATCATGAAG GTATAGAATA
TGTCTTGAAATACGTAATGG

>var001_original combination of splice junctions {2 54 94 112 139} (see Additional files 3 and 5), with 30 bases flanking the ends
ACGAGCAGAATAACCAAGATTCAGAAAATG ATGTACGATAGATACAGAGATGTGTTCAT
AAACCATATTAATGATTACGTATTAGAAGATGATATAAAAATTATAATTTCAAAATACTA

TAAACTATTATTTGATTATAACTGCTTAG GGGGTAAAAATAATAGAGGAATTTTAGTTA
TATTAATTTATGAGTATGTAAAGAATAGAGATATTAATTGTAATGAGTGGGAAAAAGTGG
CTTGTATAG CATGGTGTATAGAAATTTTACAAGCATCTTTTTTAGTAGCAGATGATATT
ATGGATAAGGGAGAAACACGCAGAAACAAACATTGTTG GTATTTGTTAAAAGACGTTGA
AATTAAGAATGCGGTGAATGATGTGTTTCTTCTGTATAACGCTATATACAA ATTACTTG
ATGTATATTTGCGCAATGATAA

>var024_original combination of splice junctions {223 236 247} (see Additional files 3 and 5), with 30 bases flanking the ends
AATAATAAATTAACGTGGCCCTTGATAAAA ATTATTATTATTATTTTTTTTTTTTTGTA
GGCATTTGAACTATGTTCACAACCTGAAAAAGAGGACATAATAAGAAATTATGGGAAAGA
TAATGTAACATGTATTAAGTTTATTAATGATATATATGAACATTATAATATCAGGGATCA
TTATGTGGAATATGAAAAGAAGCAGAAGATGAAAATATTAGA AGCCATAAACCAATTGC
ATCATGAAG GTATAGAATATGTCTTGAAATACGTAATGG

>var230_original combination of splice junctions {237 247} (see Additional files 3 and 5), with 30 bases flanking the ends
TGAAAAGAAGCAGAAGATGAAAATATTAGA CCATAAACCAATTGCATCATGAAG GTAT
AGAATATGTCTTGAAATACGTAATGG

>var339_original combination of splice junctions {239 247} (see Additional files 3 and 5), with 30 bases flanking the ends
AAATATTAGAGTAATAATATATAAGCATAA AGCCATAAACCAATTGCATCATGAAG GT
ATAGAATATGTCTTGAAATACGTAATGG

>var357_original combination of splice junctions {30 94 112 139} (see Additional files 3 and 5), with 30 bases flanking the ends
AGTTATAATAGTATATTTTTATATATGAAG GGGGTAAAAATAATAGAGGAATTTTAGTT
ATATTAATTTATGAGTATGTAAAGAATAGAGATATTAATTGTAATGAGTGGGAAAAAGTG
GCTTGTATAG CATGGTGTATAGAAATTTTACAAGCATCTTTTTTAGTAGCAGATGATAT
TATGGATAAGGGAGAAACACGCAGAAACAAACATTGTTG GTATTTGTTAAAAGACGTTG
AAATTAAGAATGCGGTGAATGATGTGTTTCTTCTGTATAACGCTATATACAA ATTACTT
GATGTATATTTGCGCAATGATAA

>var071_original combination of splice junctions {5 94 112 139 178 205} (see Additional files 3 and 5), with 30 bases flanking the ends
ATTCAGAAAATGGTCTGGATTACTTTAGAA GGGGTAAAAATAATAGAGGAATTTTAGTT
ATATTAATTTATGAGTATGTAAAGAATAGAGATATTAATTGTAATGAGTGGGAAAAAGTG
GCTTGTATAG CATGGTGTATAGAAATTTTACAAGCATCTTTTTTAGTAGCAGATGATAT
TATGGATAAGGGAGAAACACGCAGAAACAAACATTGTTG GTATTTGTTAAAAGACGTTG
AAATTAAGAATGCGGTGAATGATGTGTTTCTTCTGTATAACGCTATATACAA ATTACTT
GATGTATATTTGCGCAATGATAACTGTTACCTTGATTTAATTACATCCTTTAGAGAAGCC
ACTTTAAAAACTATAGTAGGACAACATTTAGATACAAATATATTTTCAGATAAATACTCC
CATATAGACAAAGATATAGATGTTAATAATATTAATATATCTCAAGAGAATAAAATTAAT
ATAAACATGTTAAATTTTAAGGTTTATCAAAACATTATTATTCATAAAACTGCTTATTAT
TCATTCTTTTTACCTATTGTTTGTG GTATGCAAATGGGGGGTATATCATTGGACAATTT
ATTATACAAAAAGGTCGAAAATATAGCAATTCTTATGGGGGAATATTTTCAA GTCCATG
ATGATTATATAGATACCTTTGGA

>var006_original combination of splice junctions {52} (see Additional files 3 and 5), with 30 bases flanking the ends
ATAAACTATTATTTGATTATAACTGCTTAG ATGTCTTATTAGGGGGTAAAAATAATAGA
G

>var021_original combination of splice junctions {75 112 139} (see Additional files 3 and 5), with 30 bases flanking the ends
TATATATATATGTATATGTCTTATTAGGGG CATGGTGTATAGAAATTTTACAAGCATCT
TTTTTAGTAGCAGATGATATTATGGATAAGGGAGAAACACGCAGAAACAAACATTGTTG
GTATTTGTTAAAAGACGTTGAAATTAAGAATGCGGTGAATGATGTGTTTCTTCTGTATAA
CGCTATATACAA ATTACTTGATGTATATTTGCGCAATGATAA

>var034_original combination of splice junctions {94 112 139 154 225 236 247} (see Additional files 3 and 5), with 30 bases flanking the ends
GTAATGAGTGGGAAAAAGTGGCTTGTATAG CATGGTGTATAGAAATTTTACAAGCATCT

TTTTTAGTAGCAGATGATATTATGGATAAGGGAGAAACACGCAGAAACAAACATTGTTG
GTATTTGTTAAAAGACGTTGAAATTAAGAATGCGGTGAATGATGTGTTTCTTCTGTATAA
CGCTATATACAA ATTACTTGATGTATATTTGCGCAATGATAACTGTTACCTTGATTTAA
TTACATCCTTTAGAGAAGCCACTTTAAAAACTATAGTAGGACAACATT TAGATACCTTT
GGAGATTCTAAAAAGACGGGAAAAGTTGGCTCAGATATTCAAAATAATAAATTAACGTGG
CCCTTGATAAAA GCATTTGAACTATGTTCACAACCTGAAAAAGAGGACATAATAAGAAA
TTATGGGAAAGATAATGTAACATGTATTAAGTTTATTAATGATATATATGAACATTATAA
TATCAGGGATCATTATGTGGAATATGAAAAGAAGCAGAAGATGAAAATATTAGA AGCCA
TAAACCAATTGCATCATGAAG GTATAGAATATGTCTTGAAATACGTAATGG

>var045_original combination of splice junctions {94 112 139 178 199 225 236 247} (see Additional files 3 and 5), with 30 bases flanking the ends
GTAATGAGTGGGAAAAAGTGGCTTGTATAG CATGGTGTATAGAAATTTTACAAGCATCT
TTTTTAGTAGCAGATGATATTATGGATAAGGGAGAAACACGCAGAAACAAACATTGTTG
GTATTTGTTAAAAGACGTTGAAATTAAGAATGCGGTGAATGATGTGTTTCTTCTGTATAA
CGCTATATACAA ATTACTTGATGTATATTTGCGCAATGATAACTGTTACCTTGATTTAA
TTACATCCTTTAGAGAAGCCACTTTAAAAACTATAGTAGGACAACATTTAGATACAAATA
TATTTTCAGATAAATACTCCCATATAGACAAAGATATAGATGTTAATAATATTAATATAT
CTCAAGAGAATAAAATTAATATAAACATGTTAAATTTTAAGGTTTATCAAAACATTATTA
TTCATAAAACTGCTTATTATTCATTCTTTTTACCTATTGTTTGTG GTATGCAAATGGGG
GGTATATCATTGGACAATTTATTATACAAAAAG GTCCATGATGATTATATAGATACCTT
TGGAGATTCTAAAAAGACGGGAAAAGTTGGCTCAGATATTCAAAATAATAAATTAACGTG
GCCCTTGATAAAA GCATTTGAACTATGTTCACAACCTGAAAAAGAGGACATAATAAGAA
ATTATGGGAAAGATAATGTAACATGTATTAAGTTTATTAATGATATATATGAACATTATA
ATATCAGGGATCATTATGTGGAATATGAAAAGAAGCAGAAGATGAAAATATTAGA AGCC
ATAAACCAATTGCATCATGAAG GTATAGAATATGTCTTGAAATACGTAATGG

>var169_original combination of splice junctions {94 112 139 178 205 225 236 248} (see Additional files 3 and 5), with 30 bases flanking the ends
GTAATGAGTGGGAAAAAGTGGCTTGTATAG CATGGTGTATAGAAATTTTACAAGCATCT
TTTTTAGTAGCAGATGATATTATGGATAAGGGAGAAACACGCAGAAACAAACATTGTTG
GTATTTGTTAAAAGACGTTGAAATTAAGAATGCGGTGAATGATGTGTTTCTTCTGTATAA
CGCTATATACAA ATTACTTGATGTATATTTGCGCAATGATAACTGTTACCTTGATTTAA
TTACATCCTTTAGAGAAGCCACTTTAAAAACTATAGTAGGACAACATTTAGATACAAATA
TATTTTCAGATAAATACTCCCATATAGACAAAGATATAGATGTTAATAATATTAATATAT
CTCAAGAGAATAAAATTAATATAAACATGTTAAATTTTAAGGTTTATCAAAACATTATTA
TTCATAAAACTGCTTATTATTCATTCTTTTTACCTATTGTTTGTG GTATGCAAATGGGG
GGTATATCATTGGACAATTTATTATACAAAAAGGTCGAAAATATAGCAATTCTTATGGGG
GAATATTTTCAA GTCCATGATGATTATATAGATACCTTTGGAGATTCTAAAAAGACGGG
AAAAGTTGGCTCAGATATTCAAAATAATAAATTAACGTGGCCCTTGATAAAA GCATTTG
AACTATGTTCACAACCTGAAAAAGAGGACATAATAAGAAATTATGGGAAAGATAATGTAA
CATGTATTAAGTTTATTAATGATATATATGAACATTATAATATCAGGGATCATTATGTGG
AATATGAAAAGAAGCAGAAGATGAAAATATTAGA AGCCATAAACCAATTGCATCATGAA
G AATATGTCTTGAAATACGTAATGGACATTT

>var017_original combination of splice junctions {97 112 139 178 205 225 236 247} (see Additional files 3 and 5), with 30 bases flanking the ends
GTAATGAGTGGGAAAAAGTGGCTTGTATAG TAGCAGATGATATTATGGATAAGGGAGAA
ACACGCAGAAACAAACATTGTTG GTATTTGTTAAAAGACGTTGAAATTAAGAATGCGGT
GAATGATGTGTTTCTTCTGTATAACGCTATATACAA ATTACTTGATGTATATTTGCGCA
ATGATAACTGTTACCTTGATTTAATTACATCCTTTAGAGAAGCCACTTTAAAAACTATAG
TAGGACAACATTTAGATACAAATATATTTTCAGATAAATACTCCCATATAGACAAAGATA
TAGATGTTAATAATATTAATATATCTCAAGAGAATAAAATTAATATAAACATGTTAAATT
TTAAGGTTTATCAAAACATTATTATTCATAAAACTGCTTATTATTCATTCTTTTTACCTA
TTGTTTGTG GTATGCAAATGGGGGGTATATCATTGGACAATTTATTATACAAAAAGGTC
GAAAATATAGCAATTCTTATGGGGGAATATTTTCAA GTCCATGATGATTATATAGATAC
CTTTGGAGATTCTAAAAAGACGGGAAAAGTTGGCTCAGATATTCAAAATAATAAATTAAC
GTGGCCCTTGATAAAA GCATTTGAACTATGTTCACAACCTGAAAAAGAGGACATAATAA
GAAATTATGGGAAAGATAATGTAACATGTATTAAGTTTATTAATGATATATATGAACATT
ATAATATCAGGGATCATTATGTGGAATATGAAAAGAAGCAGAAGATGAAAATATTAGA A
GCCATAAACCAATTGCATCATGAAG GTATAGAATATGTCTTGAAATACGTAATGG

####
Below, the "padded" versions of the sequences above

>var009_padded
ATGTACGATAGATACAGAGATGTGTTCATAAACCATATTAATGATTACGTATTAGAAGAT
GATATAAAAATTATAATTTCAAAATACTATAAACTATTATTTGATTATAACTGCTTAG G
GGGTAAAAATAATAGAGGAATTTTAGTTATATTAATTTATGAGTATGTAAAGAATAGAGA
TATTAATTGTAATGAGTGGGAAAAAGTGGCTTGTATAG CATGGTGTATAGAAATTTTAC
AAGCATCTTTTTTAGTAGCAGATGATATTATGGATAAGGGAGAAACACGCAGAAACAAAC
ATTGTTG GTATTTGTTAAAAGACGTTGAAATTAAGAATGCGGTGAATGATGTGTTTCTT
CTGTATAACGCTATATACAA ATTACTTGATGTATATTTGCGCAATGATAACTGTTACCT
TGATTTAATTACATCCTTTAGAGAAGCCACTTTAAAAACTATAGTAGGACAACATTTAGA
TACAAATATATTTTCAGATAAATACTCCCATATAGACAAAGATATAGATGTTAATAATAT
TAATATATCTCAAGAGAATAAAATTAATATAAACATGTTAAATTTTAAGGTTTATCAAAA
CATTATTATTCATAAAACTGCTTATTATTCATTCTTTTTACCTATTGTTTGTG GTATGC
AAATGGGGGGTATATCATTGGACAATTTATTATACAAAAAGGTCGAAAATATAGCAATTC
TTATGGGGGAATATTTTCAA GTCCATGATGATTATATAGATACCTTTGGAGATTCTAAA
AAGACGGGAAAAGTTGGCTCAGATATTCAAAATAATAAATTAACGTGGCCCTTGATAAAA
 GCATTTGAACTATGTTCACAACCTGAAAAAGAGGACATAATAAGAAATTATGGGAAAGA
TAATGTAACATGTATTAAGTTTATTAATGATATATATGAACATTATAATATCAGGGATCA
TTATGTGGAATATGAAAAGAAGCAGAAGATGAAAATATTAGA AGCCATAAACCAATTGC
ATCATGAAG GTATAGAATATGTCTTGAAATACGTAATGGACATTTTGTTTACAGGCGCT
TGA

>var045_padded
ATGGAGAACGAGCAGAATAACCAAGATTCAGAAAATGGTCTGGATTACTTTAGAAGT AT
GTACGATAGATACAGAGATGTGTTCATAAACCATATTAATGATTACGTATTAGAAGATGA
TATAAAAATTATAATTTCAAAATACTATAAACTATTATTTGATTATAACTGCTTAG GGG
GTAAAAATAATAGAGGAATTTTAGTTATATTAATTTATGAGTATGTAAAGAATAGAGATA
TTAATTGTAATGAGTGGGAAAAAGTGGCTTGTATAG CATGGTGTATAGAAATTTTACAA
GCATCTTTTTTAGTAGCAGATGATATTATGGATAAGGGAGAAACACGCAGAAACAAACAT
TGTTG GTATTTGTTAAAAGACGTTGAAATTAAGAATGCGGTGAATGATGTGTTTCTTCT
GTATAACGCTATATACAA ATTACTTGATGTATATTTGCGCAATGATAACTGTTACCTTG
ATTTAATTACATCCTTTAGAGAAGCCACTTTAAAAACTATAGTAGGACAACATTTAGATA
CAAATATATTTTCAGATAAATACTCCCATATAGACAAAGATATAGATGTTAATAATATTA
ATATATCTCAAGAGAATAAAATTAATATAAACATGTTAAATTTTAAGGTTTATCAAAACA
TTATTATTCATAAAACTGCTTATTATTCATTCTTTTTACCTATTGTTTGTG GTATGCAA
ATGGGGGGTATATCATTGGACAATTTATTATACAAAAAG GTCCATGATGATTATATAGA
TACCTTTGGAGATTCTAAAAAGACGGGAAAAGTTGGCTCAGATATTCAAAATAATAAATT
AACGTGGCCCTTGATAAAA GCATTTGAACTATGTTCACAACCTGAAAAAGAGGACATAA
TAAGAAATTATGGGAAAGATAATGTAACATGTATTAAGTTTATTAATGATATATATGAAC
ATTATAATATCAGGGATCATTATGTGGAATATGAAAAGAAGCAGAAGATGAAAATATTAG
A AGCCATAAACCAATTGCATCATGAAG GTATAGAATATGTCTTGAAATACGTAATGGA
CATTTTGTTTACAGGCGCTTGA

>var169_padded
ATGGAGAACGAGCAGAATAACCAAGATTCAGAAAATGGTCTGGATTACTTTAGAAGT AT
GTACGATAGATACAGAGATGTGTTCATAAACCATATTAATGATTACGTATTAGAAGATGA
TATAAAAATTATAATTTCAAAATACTATAAACTATTATTTGATTATAACTGCTTAG GGG
GTAAAAATAATAGAGGAATTTTAGTTATATTAATTTATGAGTATGTAAAGAATAGAGATA
TTAATTGTAATGAGTGGGAAAAAGTGGCTTGTATAG CATGGTGTATAGAAATTTTACAA
GCATCTTTTTTAGTAGCAGATGATATTATGGATAAGGGAGAAACACGCAGAAACAAACAT
TGTTG GTATTTGTTAAAAGACGTTGAAATTAAGAATGCGGTGAATGATGTGTTTCTTCT
GTATAACGCTATATACAA ATTACTTGATGTATATTTGCGCAATGATAACTGTTACCTTG
ATTTAATTACATCCTTTAGAGAAGCCACTTTAAAAACTATAGTAGGACAACATTTAGATA
CAAATATATTTTCAGATAAATACTCCCATATAGACAAAGATATAGATGTTAATAATATTA
ATATATCTCAAGAGAATAAAATTAATATAAACATGTTAAATTTTAAGGTTTATCAAAACA
TTATTATTCATAAAACTGCTTATTATTCATTCTTTTTACCTATTGTTTGTG GTATGCAA
ATGGGGGGTATATCATTGGACAATTTATTATACAAAAAGGTCGAAAATATAGCAATTCTT
ATGGGGGAATATTTTCAA GTCCATGATGATTATATAGATACCTTTGGAGATTCTAAAAA
GACGGGAAAAGTTGGCTCAGATATTCAAAATAATAAATTAACGTGGCCCTTGATAAAA G
CATTTGAACTATGTTCACAACCTGAAAAAGAGGACATAATAAGAAATTATGGGAAAGATA
ATGTAACATGTATTAAGTTTATTAATGATATATATGAACATTATAATATCAGGGATCATT
ATGTGGAATATGAAAAGAAGCAGAAGATGAAAATATTAGA AGCCATAAACCAATTGCAT

CATGAAG AATATGTCTTGAAATACGTAATGGACATTTTGTTTACAGGCGCTTGA

>var148_padded
ATGGAGAACGAGCAGAATAACCAAGATTCAGAAAATGGTCTGGATTACTTTAGAAGT AT
GTACGATAGATACAGAGATGTGTTCATAAACCATATTAATGATTACGTATTAGAAGATGA
TATAAAAATTATAATTTCAAAATACTATAAACTATTATTTGATTATAACTGCTTAG GGG
GTAAAAATAATAGAGGAATTTTAGTTATATTAATTTATGAGTATGTAAAGAATAGAGATA
TTAATTGTAATGAGTGGGAAAAAGTGGCTTGTATAG CATGGTGTATAGAAATTTTACAA
GCATCTTTTTTAGTAGCAGATGATATTATGGATAAGGGAGAAACACGCAGAAACAAACAT
TGTTG GTATTTGTTAAAAGACGTTGAAATTAAGAATGCGGTGAATGATGTGTTTCTTCT
GTATAACGCTATATACAA ATTACTTGATGTATATTTGCGCAATGATAACTGTTACCTTG
ATTTAATTACATCCTTTAGAGAAGCCACTTTAAAAACTATAGTAGGACAACATTTAGATA
CAAATATATTTTCAGATAAATACTCCCATATAGACAAAGATATAGATGTTAATAATATTA
ATATATCTCAAGAGAATAAAATTAATATAAACATGTTAAATTTTAAGGTTTATCAAAACA
TTATTATTCATAAAACTGCTTATTATTCATTCTTTTTACCTATTGTTTGTG GTATGCAA
ATGGGGGGTATATCATTGGACAATTTATTATACAAAAAGGTCGAAAATATAGCAATTCTT
ATGGGGGAATATTTTCAA GTCCATGATGATTATATAGATACCTTTGGAGATTCTAAAAA
GACGGGAAAAGTTGGCTCAGATATTCAAAATAATAAATTAACGTGGCCCTTGATAAAA G
CATTTGAACTATGTTCACAACCTGAAAAAGAGGACATAATAAGAAATTATGGGAAAGATA
ATGTAACATGTATTAAGTTTATTAATGATATATATGAACATTATAATATCAGGGATCATT
ATGTGGAATATGAAAAGAAGCAGAAGATGAAAATATTAGA GTATAGAATATGTCTTGAA
ATACGTAATGGACATTTTGTTTACAGGCGCTTGAGAGATAACAACCTTAACAACTTTTGT
TTAAATATGATCAAGTTTAAAAAAAAAAAAAAAATAA

>var208_padded
ATGGAGAACGAGCAGAATAACCAAGATTCAGAAAATGGTCTGGATTACTTTAGAAGT AT
GTACGATAGATACAGAGATGTGTTCATAAACCATATTAATGATTACGTATTAGA AGATG
ATATTATGGATAAGGGAGAAACACGCAGAAACAAACATTGTTG GTATTTGTTAAAAGAC
GTTGAAATTAAGAATGCGGTGAATGATGTGTTTCTTCTGTATAACGCTATATACAA ATT
ACTTGATGTATATTTGCGCAATGATAACTGTTACCTTGATTTAATTACATCCTTTAGAGA
AGCCACTTTAAAAACTATAGTAGGACAACATTTAGATACAAATATATTTTCAGATAAATA
CTCCCATATAGACAAAGATATAGATGTTAATAATATTAATATATCTCAAGAGAATAAAAT
TAATATAAACATGTTAAATTTTAAGGTTTATCAAAACATTATTATTCATAAAACTGCTTA
TTATTCATTCTTTTTACCTATTGTTTGTG GTATGCAAATGGGGGGTATATCATTGGACA
ATTTATTATACAAAAAGGTCGAAAATATAGCAATTCTTATGGGGGAATATTTTCAA GTC
CATGATGATTATATAGATACCTTTGGAGATTCTAAAAAGACGGGAAAAGTTGGCTCAGAT
ATTCAAAATAATAAATTAACGTGGCCCTTGATAAAA GCATTTGAACTATGTTCACAACC
TGAAAAAGAGGACATAATAAGAAATTATGGGAAAGATAATGTAACATGTATTAAGTTTAT
TAATGATATATATGAACATTATAATATCAGGGATCATTATGTGGAATATGAAAAGAAGCA
GAAGATGAAAATATTAGA AGCCATAAACCAATTGCATCATGAAG GTATAGAATATGTC
TTGAAATACGTAATGGACATTTTGTTTACAGGCGCTTGA

>var078_padded
ATGGAGAACGAGCAGAATAACCAAGATTCAGAAAATGGTCTGGATTACTTTAGAAGT AT
GTACGATAGATACAGAGATGTGTTCATAAACCATATTAATGATTACGTATTAGAAGATGA
TATAAAAATTATAATTTCAAAATACTATAAACTATTAT GGGGTAAAAATAATAGAGGAA
TTTTAGTTATATTAATTTATGAGTATGTAAAGAATAGAGATATTAATTGTAATGAGTGGG
AAAAAGTGGCTTGTATAG CATGGTGTATAGAAATTTTACAAGCATCTTTTTTAGTAGCA
GATGATATTATGGATAAGGGAGAAACACGCAGAAACAAACATTGTTG GTATTTGTTAAA
AGACGTTGAAATTAAGAATGCGGTGAATGATGTGTTTCTTCTGTATAACGCTATATACAA
ATTACTTGATGTATATTTGCGCAATGATAACTGTTACCTTGATTTAATTACATCCTTTA
GAGAAGCCACTTTAAAAACTATAGTAGGACAACATTTAGATACAAATATATTTTCAGATA
AATACTCCCATATAGACAAAGATATAGATGTTAATAATATTAATATATCTCAAGAGAATA
AAATTAATATAAACATGTTAAATTTTAAGGTTTATCAAAACATTATTATTCATAAAACTG
CTTATTATTCATTCTTTTTACCTATTGTTTGTG GTATGCAAATGGGGGGTATATCATTG
GACAATTTATTATACAAAAAGGTCGAAAATATAGCAATTCTTATGGGGGAATATTTTCAA
GTCCATGATGATTATATAGATACCTTTGGAGATTCTAAAAAGACGGGAAAAGTTGGCTC
AGATATTCAAAATAATAAATTAACGTGGCCCTTGATAAAA GCATTTGAACTATGTTCAC
AACCTGAAAAAGAGGACATAATAAGAAATTATGGGAAAGATAATGTAACATGTATTAAGT
TTATTAATGATATATATGAACATTATAATATCAGGGATCATTATGTGGAATATGAAAAGA
AGCAGAAGATGAAAATATTAGA AGCCATAAACCAATTGCATCATGAAG GTATAGAATA
TGTCTTGAAATACGTAATGGACATTTTGTTTACAGGCGCTTGA

>var036_padded

ATGGAGAACGAGCAGAATAACCAAGATTCAGAAAATGGTCTGGATTACTTTAGAAGT AT
GTACGATAGATACAGAGATGTGTTCATAAACCATATTAATGATTACGTATTAGAAGATGA
TATAAAAATTATAATTTCAAAATACTATAAACTATTATTTGATTATAACTGCTTAG CAT
GGTGTATAGAAATTTTACAAGCATCTTTTTTAGTAGCAGATGATATTATGGATAAGGGAG
AAACACGCAGAAACAAACATTGTTG GTATTTGTTAAAAGACGTTGAAATTAAGAATGCG
GTGAATGATGTGTTTCTTCTGTATAACGCTATATACAA ATTACTTGATGTATATTTGCG
CAATGATAACTGTTACCTTGATTTAATTACATCCTTTAGAGAAGCCACTTTAAAAACTAT
AGTAGGACAACATTTAGATACAAATATATTTTCAGATAAATACTCCCATATAGACAAAGA
TATAGATGTTAATAATATTAATATATCTCAAGAGAATAAAATTAATATAAACATGTTAAA
TTTTAAGGTTTATCAAAACATTATTATTCATAAAACTGCTTATTATTCATTCTTTTTACC
TATTGTTTGTG GTATGCAAATGGGGGGTATATCATTGGACAATTTATTATACAAAAAGG
TCGAAAATATAGCAATTCTTATGGGGGAATATTTTCAA GTCCATGATGATTATATAGAT
ACCTTTGGAGATTCTAAAAAGACGGGAAAAGTTGGCTCAGATATTCAAAATAATAAATTA
ACGTGGCCCTTGATAAAA GCATTTGAACTATGTTCACAACCTGAAAAAGAGGACATAAT
AAGAAATTATGGGAAAGATAATGTAACATGTATTAAGTTTATTAATGATATATATGAACA
TTATAATATCAGGGATCATTATGTGGAATATGAAAAGAAGCAGAAGATGAAAATATTAGA
 AGCCATAAACCAATTGCATCATGAAG GTATAGAATATGTCTTGAAATACGTAATGGAC
ATTTTGTTTACAGGCGCTTGA

>var038_padded
ATGGAGAACGAGCAGAATAACCAAGATTCAGAAAATGGTCTGGATTACTTTAGAAGT AT
GTACGATAGATACAGAGATGTGTTCATAAACCATATTAATGATTACGTATTAGAAGATGA
TATAAAAATTATAATTTCAAAATACTATAAACTATTATTTGATTATAACTGCTTAG TAG
CAGATGATATTATGGATAAGGGAGAAACACGCAGAAACAAACATTGTTG GTATTTGTTA
AAAGACGTTGAAATTAAGAATGCGGTGAATGATGTGTTTCTTCTGTATAACGCTATATAC
AA ATTACTTGATGTATATTTGCGCAATGATAACTGTTACCTTGATTTAATTACATCCTT
TAGAGAAGCCACTTTAAAAACTATAGTAGGACAACATTTAGATACAAATATATTTTCAGA
TAAATACTCCCATATAGACAAAGATATAGATGTTAATAATATTAATATATCTCAAGAGAA
TAAAATTAATATAAACATGTTAAATTTTAAGGTTTATCAAAACATTATTATTCATAAAAC
TGCTTATTATTCATTCTTTTTACCTATTGTTTGTG GTATGCAAATGGGGGGTATATCAT
TGGACAATTTATTATACAAAAAGGTCGAAAATATAGCAATTCTTATGGGGGAATATTTTC
AA GTCCATGATGATTATATAGATACCTTTGGAGATTCTAAAAAGACGGGAAAAGTTGGC
TCAGATATTCAAAATAATAAATTAACGTGGCCCTTGATAAAA GCATTTGAACTATGTTC
ACAACCTGAAAAAGAGGACATAATAAGAAATTATGGGAAAGATAATGTAACATGTATTAA
GTTTATTAATGATATATATGAACATTATAATATCAGGGATCATTATGTGGAATATGAAAA
GAAGCAGAAGATGAAAATATTAGA AGCCATAAACCAATTGCATCATGAAG GTATAGAA
TATGTCTTGAAATACGTAATGGACATTTTGTTTACAGGCGCTTGA

>var094_padded
ATGGAGAACGAGCAGAATAACCAAGATTCAGAAAATGGTCTGGATTACTTTAGAAGT AT
GTACGATAGATACAGAGATGTGTTCATAAACCATATTAATGATTACGTATTAGAAGATGA
TATAAAAATTATAATTTCAAAATACTATAAACTATTATTTGATTATAACTGCTTAG GGG
GTAAAAATAATAGAGGAATTTTAGTTATATTAATTTATGA GTATTTGTTAAAAGACGTT
GAAATTAAGAATGCGGTGAATGATGTGTTTCTTCTGTATAACGCTATATACAA ATTACT
TGATGTATATTTGCGCAATGATAACTGTTACCTTGATTTAATTACATCCTTTAGAGAAGC
CACTTTAAAAACTATAGTAGGACAACATTTAGATACAAATATATTTTCAGATAAATACTC
CCATATAGACAAAGATATAGATGTTAATAATATTAATATATCTCAAGAGAATAAAATTAA
TATAAACATGTTAAATTTTAAGGTTTATCAAAACATTATTATTCATAAAACTGCTTATTA
TTCATTCTTTTTACCTATTGTTTGTG GTATGCAAATGGGGGGTATATCATTGGACAATT
TATTATACAAAAAGGTCGAAAATATAGCAATTCTTATGGGGGAATATTTTCAA GTCCAT
GATGATTATATAGATACCTTTGGAGATTCTAAAAAGACGGGAAAAGTTGGCTCAGATATT
CAAAATAATAAATTAACGTGGCCCTTGATAAAA GCATTTGAACTATGTTCACAACCTGA
AAAAGAGGACATAATAAGAAATTATGGGAAAGATAATGTAACATGTATTAAGTTTATTAA
TGATATATATGAACATTATAATATCAGGGATCATTATGTGGAATATGAAAAGAAGCAGAA
GATGAAAATATTAGA AGCCATAAACCAATTGCATCATGAAG GTATAGAATATGTCTTG
AAATACGTAATGGACATTTTGTTTACAGGCGCTTGA

>var124_padded
ATGGAGAACGAGCAGAATAACCAAGATTCAGAAAATGGTCTGGATTACTTTAGAAGT AT
GTACGATAGATACAGAGATGTGTTCATAAACCATATTAATGATTACGTATTAGAAGATGA
TATAAAAATTATAATTTCAAAATACTATAAACTATTATTTGATTATAACTGCTTAG GGG
GTAAAAATAATAGAGGAATTTTAGTTATATTAATTTATGAGTATGTAAAGAATAGAGATA
TTAATTGTA TAGCATGGTGTATAGAAATTTTACAAGCATCTTTTTTAGTAGCAGATGAT
ATTATGGATAAGGGAGAAACACGCAGAAACAAACATTGTTG GTATTTGTTAAAAGACGT

TGAAATTAAGAATGCGGTGAATGATGTGTTTCTTCTGTATAACGCTATATACAA ATTAC
TTGATGTATATTTGCGCAATGATAACTGTTACCTTGATTTAATTACATCCTTTAGAGAAG
CCACTTTAAAAACTATAGTAGGACAACATTTAGATACAAATATATTTTCAGATAAATACT
CCCATATAGACAAAGATATAGATGTTAATAATATTAATATATCTCAAGAGAATAAAATTA
ATATAAACATGTTAAATTTTAAGGTTTATCAAAACATTATTATTCATAAAACTGCTTATT
ATTCATTCTTTTTACCTATTGTTTGTG GTATGCAAATGGGGGGTATATCATTGGACAAT
TTATTATACAAAAAGGTCGAAAATATAGCAATTCTTATGGGGGAATATTTTCAA GTCCA
TGATGATTATATAGATACCTTTGGAGATTCTAAAAAGACGGGAAAAGTTGGCTCAGATAT
TCAAAATAATAAATTAACGTGGCCCTTGATAAAA GCATTTGAACTATGTTCACAACCTG
AAAAAGAGGACATAATAAGAAATTATGGGAAAGATAATGTAACATGTATTAAGTTTATTA
ATGATATATATGAACATTATAATATCAGGGATCATTATGTGGAATATGAAAAGAAGCAGA
AGATGAAAATATTAGA AGCCATAAACCAATTGCATCATGAAG GTATAGAATATGTCTT
GAAATACGTAATGGACATTTTGTTTACAGGCGCTTGA

>var157_padded
ATGGAGAACGAGCAGAATAACCAAGATTCAGAAAATGGTCTGGATTACTTTAGAAGT AT
GTACGATAGATACAGAGATGTGTTCATAAACCATATTAATGATTACGTATTAGAAGATGA
TATAAAAATTATAATTTCAAAATACTATAAACTATTATTTGATTATAACTGCTTAG GGG
GTAAAAATAATAGAGGAATTTTAGTTATATTAATTTATGAGTATGTAAAGAATAGAGATA
TTAATTGTAATGAGTGGGAAAAAGTGGCTTGTATAG CATGGTGTATAGAAATTTTACAA
GCATCTTTTTTAGTAGCAGATGATATTATGGATAAGGGAGAAACACGCAGAAACAAACAT
TGTTG GTATTTGTTAAAAGACGTTGAAATTAAGAATGCGGTGAATGATGTGTTTCTTCT
GTATAACGCTATATACAA ATTACTTGATGTATATTTGCGCAATGATAACTGTTACCTTG
ATTTAATTACATCCTTTAGAGAAGCCACTTTAAAAACTATA GTTTATCAAAACATTATT
ATTCATAAAACTGCTTATTATTCATTCTTTTTACCTATTGTTT GTATGCAAATGGGGGG
TATATCATTGGACAATTTATTATACAAAAAGGTCGAAAATATAGCAATTCTTATGGGGGA
ATATTTTCAA GTCCATGATGATTATATAGATACCTTTGGAGATTCTAAAAAGACGGGAA
AAGTTGGCTCAGATATTCAAAATAATAAATTAACGTGGCCCTTGATAAAA GCATTTGAA
CTATGTTCACAACCTGAAAAAGAGGACATAATAAGAAATTATGGGAAAGATAATGTAACA
TGTATTAAGTTTATTAATGATATATATGAACATTATAATATCAGGGATCATTATGTGGAA
TATGAAAAGAAGCAGAAGATGAAAATATTAGA AGCCATAAACCAATTGCATCATGAAG
GTATAGAATATGTCTTGAAATACGTAATGGACATTTTGTTTACAGGCGCTTGA

>var107_padded
ATGGAGAACGAGCAGAATAACCAAGATTCAGAAAATGGTCTGGATTACTTTAGAAGT AT
GTACGATAGATACAGAGATGTGTTCATAAACCATATTAATGATTACGTATTAGAAGATGA
TATAAAAATTATAATTTCAAAATACTATAAACTATTATTTGATTATAACTGCTTAG GGG
GTAAAAATAATAGAGGAATTTTAGTTATATTAATTTATGAGTATGTAAAGAATAGAGATA
TTAATTGTAATGAGTGGGAAAAAGTGGCTTGTATAG CATGGTGTATAGAAATTTTACAA
GCATCTTTTTTAGTAGCAGATGATATTATGGATAAGGGAGAAACACGCAGAAACAAACAT
TGTTG GTATTTGTTAAAAGACGTTGAAATTAAGAATGCGGTGAATGATGTGTTTCTTCT
GTATAACGCTATATACAA ATTACTTGATGTATATTTGCGCAATGATAACTGTTACCTTG
ATTTAATTACATCCTTTAGAGAAGCCACTTTAAAAACTATA GTTTATCAAAACATTATT
ATTCATAAAACTGCTTATTATTCATTCTTTTTACCTATTGTTTGTG GTATGCAAATGGG
GGGTATATCATTGGACAATTTATTATACAAAAAGGTCGAAAATATAGCAATTCTTATGGG
GGAATATTTTCAA GTCCATGATGATTATATAGATACCTTTGGAGATTCTAAAAAGACGG
GAAAAGTTGGCTCAGATATTCAAAATAATAAATTAACGTGGCCCTTGATAAAA GCATTT
GAACTATGTTCACAACCTGAAAAAGAGGACATAATAAGAAATTATGGGAAAGATAATGTA
ACATGTATTAAGTTTATTAATGATATATATGAACATTATAATATCAGGGATCATTATGTG
GAATATGAAAAGAAGCAGAAGATGAAAATATTAGA AGCCATAAACCAATTGCATCATGA
AG GTATAGAATATGTCTTGAAATACGTAATGGACATTTTGTTTACAGGCGCTTGA

>var060_padded
ATGGAGAACGAGCAGAATAACCAAGATTCAGAAAATGGTCTGGATTACTTTAGAAGT AT
GTACGATAGATACAGAGATGTGTTCATAAACCATATTAATGATTACGTATTAGAAGATGA
TATAAAAATTATAATTTCAAAATACTATAAACTATTATTTGATTATAACTGCTTAG GGG
GTAAAAATAATAGAGGAATTTTAGTTATATTAATTTATGAGTATGTAAAGAATAGAGATA
TTAATTGTAATGAGTGGGAAAAAGTGGCTTGTATAG CATGGTGTATAGAAATTTTACAA
GCATCTTTTTTAGTAGCAGATGATATTATGGATAAGGGAGAAACACGCAGAAACAAACAT
TGTTG GTATTTGTTAAAAGACGTTGAAATTAAGAATGCGGTGAATGATGTGTTTCTTCT
GTATAACGCTATATACAA ATTACTTGATGTATATTTGCGCAATGATAACTGTTACCTTG
ATTTAATTACATCCTTTAGAGAAGCCACTTTAAAAACTATAGTAGGACAACATTTAGATA
CAAATATATTTTCAGATAAATACTCCCATATAGACAAAGATATAGATGTTAATAATATTA
ATATATCTCAAGAGAATAAAATTAATATAAACATGTTAAATTTTAAGGTTTATCAAAACA

TTATTATTCATAAAACTGCTTATTATTCATTCTTTTTACCTATTGTTT GTATGCAAATG
GGGGGTATATCATTGGACAATTTATTATACAAAAAGGTCGAAAATATAGCAATTCTTATG
GGGGAATATTTTCAA GTCCATGATGATTATATAGATACCTTTGGAGATTCTAAAAAGAC
GGGAAAAGTTGGCTCAGATATTCAAAATAATAAATTAACGTGGCCCTTGATAAAA GCAT
TTGAACTATGTTCACAACCTGAAAAAGAGGACATAATAAGAAATTATGGGAAAGATAATG
TAACATGTATTAAGTTTATTAATGATATATATGAACATTATAATATCAGGGATCATTATG
TGGAATATGAAAAGAAGCAGAAGATGAAAATATTAGA AGCCATAAACCAATTGCATCAT
GAAG GTATAGAATATGTCTTGAAATACGTAATGGACATTTTGTTTACAGGCGCTTGA

>var067_padded
ATGGAGAACGAGCAGAATAACCAAGATTCAGAAAATGGTCTGGATTACTTTAGAAGT AT
GTACGATAGATACAGAGATGTGTTCATAAACCATATTAATGATTACGTATTAGAAGATGA
TATAAAAATTATAATTTCAAAATACTATAAACTATTATTTGATTATAACTGCTTAG GGG
GTAAAAATAATAGAGGAATTTTAGTTATATTAATTTATGAGTATGTAAAGAATAGAGATA
TTAATTGTAATGAGTGGGAAAAAGTGGCTTGTATAG CATGGTGTATAGAAATTTTACAA
GCATCTTTTTTAGTAGCAGATGATATTATGGATAAGGGAGAAACACGCAGAAACAAACAT
TGTTG GTATTTGTTAAAAGACGTTGAAATTAAGAATGCGGTGAATGATGTGTTTCTTCT
GTATAACGCTATATACAA ATTACTTGATGTATATTTGCGCAATGATAACTGTTACCTTG
ATTTAATTACATCCTTTAGAGAAGCCACTTTAAAAACTATAGTAGGACAACATTTAGATA
CAAATATATTTTCAGATAAATACTCCCATATAGACAAAGATATAGATGTTAATAATATTA
ATATATCTCAAGAGAATAAAATTAATATAAACATGTTAAATTTTAAGGTTTATCAAAACA
TTATTATTCATAAAACTGCTTATTATTCATTCTTTTTACCTATTGTTTGT GTATGCAAA
TGGGGGGTATATCATTGGACAATTTATTATACAAAAAGGTCGAAAATATAG

>var156_padded
ATGGAGAACGAGCAGAATAACCAAGATTCAGAAAATGGTCTGGATTACTTTAGAAGT AT
GTACGATAGATACAGAGATGTGTTCATAAACCATATTAATGATTACGTATTAGAAGATGA
TATAAAAATTATAATTTCAAAATACTATAAACTATTATTTGATTATAACTGCTTAG GGG
GTAAAAATAATAGAGGAATTTTAGTTATATTAATTTATGAGTATGTAAAGAATAGAGATA
TTAATTGTAATGAGTGGGAAAAAGTGGCTTGTATAG CATGGTGTATAGAAATTTTACAA
GCATCTTTTTTAGTAGCAGATGATATTATGGATAAGGGAGAAACACGCAGAAACAAACAT
TGTTG GTATTTGTTAAAAGACGTTGAAATTAAGAATGCGGTGAATGATGTGTTTCTTCT
GTATAACGCTATATACAA AATTACATCCTTTAGAGAAGCCACTTTAAAAACTATAGTAG
GACAACATTTAGATACAAATATATTTTCAGATAAATACTCCCATATAGACAAAGATATAG
ATGTTAATAATATTAATATATCTCAAGAGAATAAAATTAATATAAACATGTTAAATTTTA
AGGTTTATCAAAACATTATTATTCATAAAACTGCTTATTATTCATTCTTTTTACCTATTG
TTTGTG GTATGCAAATGGGGGGTATATCATTGGACAATTTATTATACAAAAAGGTCGAA
AATATAGCAATTCTTATGGGGGAATATTTTCAA GTCCATGATGATTATATAGATACCTT
TGGAGATTCTAAAAAGACGGGAAAAGTTGGCTCAGATATTCAAAATAATAAATTAACGTG
GCCCTTGATAAAA GCATTTGAACTATGTTCACAACCTGAAAAAGAGGACATAATAAGAA
ATTATGGGAAAGATAATGTAACATGTATTAAGTTTATTAATGATATATATGAACATTATA
ATATCAGGGATCATTATGTGGAATATGAAAAGAAGCAGAAGATGAAAATATTAGA AGCC
ATAAACCAATTGCATCATGAAG GTATAGAATATGTCTTGAAATACGTAATGGACATTTT
GTTTACAGGCGCTTGA

>var151_padded
ATGGAGAACGAGCAGAATAACCAAGATTCAGAAAATGGTCTGGATTACTTTAGAAGT AT
GTACGATAGATACAGAGATGTGTTCATAAACCATATTAATGATTACGTATTAGAAGATGA
TATAAAAATTATAATTTCAAAATACTATAAACTATTATTTGATTATAACTGCTTAG GGG
GTAAAAATAATAGAGGAATTTTAGTTATATTAATTTATGAGTATGTAAAGAATAGAGATA
TTAATTGTAATGAGTGGGAAAAAGTGGCTTGTATAG CATGGTGTATAGAAATTTTACAA
GCATCTTTTTTAGTAGCAGATGATATTATGGATAAGGGAGAAACACGCAGAAACAAACAT
TGTTG GTATTTGTTAAAAGACGTTGAAATTAAGAATGCGGTGAATGATGTGTTTCTTCT
GTATAACGCTATATACAA AGAAGCCACTTTAAAAACTATAGTAGGACAACATTTAGATA
CAAATATATTTTCAGATAAATACTCCCATATAGACAAAGATATAGATGTTAATAATATTA
ATATATCTCAAGAGAATAAAATTAATATAAACATGTTAAATTTTAAGGTTTATCAAAACA
TTATTATTCATAAAACTGCTTATTATTCATTCTTTTTACCTATTGTTTGTG GTATGCAA
ATGGGGGGTATATCATTGGACAATTTATTATACAAAAAGGTCGAAAATATAGCAATTCTT
ATGGGGGAATATTTTCAA GTCCATGATGATTATATAGATACCTTTGGAGATTCTAAAAA
GACGGGAAAAGTTGGCTCAGATATTCAAAATAATAAATTAACGTGGCCCTTGATAAAA G
CATTTGAACTATGTTCACAACCTGAAAAAGAGGACATAATAAGAAATTATGGGAAAGATA
ATGTAACATGTATTAAGTTTATTAATGATATATATGAACATTATAATATCAGGGATCATT
ATGTGGAATATGAAAAGAAGCAGAAGATGAAAATATTAGA AGCCATAAACCAATTGCAT
CATGAAG GTATAGAATATGTCTTGAAATACGTAATGGACATTTTGTTTACAGGCGCTTG

A

>var128_padded
ATGGAGAACGAGCAGAATAACCAAGATTCAGAAAATGGTCTGGATTACTTTAGAAGT AT
GTACGATAGATACAGAGATGTGTTCATAAACCATATTAATGATTACGTATTAGAAGATGA
TATAAAAATTATAATTTCAAAATACTATAAACTATTATTTGATTATAACTGCTTAG GGG
GTAAAAATAATAGAGGAATTTTAGTTATATTAATTTATGAGTATGTAAAGAATAGAGATA
TTAATTGTAATGAGTGGGAAAAAGTGGCTTGTATAG CATGGTGTATAGAAATTTTACAA
GCATCTTTTTTAGTAGCAGATGATATTATGGATAAGGGAGAAACACGCAGAAACAAACAT
TGTTG ATTACTTGATGTATATTTGCGCAATGATAACTGTTACCTTGATTTAATTACATC
CTTTAGAGAAGCCACTTTAAAAACTATAGTAGGACAACATTTAGATACAAATATATTTTC
AGATAAATACTCCCATATAGACAAAGATATAGATGTTAATAATATTAATATATCTCAAGA
GAATAAAATTAATATAAACATGTTAAATTTTAAGGTTTATCAAAACATTATTATTCATAA
AACTGCTTATTATTCATTCTTTTTACCTATTGTTTGTG GTATGCAAATGGGGGGTATAT
CATTGGACAATTTATTATACAAAAAGGTCGAAAATATAGCAATTCTTATGGGGGAATATT
TTCAA GTCCATGATGATTATATAGATACCTTTGGAGATTCTAAAAAGACGGGAAAAGTT
GGCTCAGATATTCAAAATAATAAATTAACGTGGCCCTTGATAAAA GCATTTGAACTATG
TTCACAACCTGAAAAAGAGGACATAATAAGAAATTATGGGAAAGATAATGTAACATGTAT
TAAGTTTATTAATGATATATATGAACATTATAATATCAGGGATCATTATGTGGAATATGA
AAAGAAGCAGAAGATGAAAATATTAGA AGCCATAAACCAATTGCATCATGAAG GTATA
GAATATGTCTTGAAATACGTAATGGACATTTTGTTTACAGGCGCTTGA

>var075_padded
ATGGAGAACGAGCAGAATAACCAAGATTCAGAAAATGGTCTGGATTACTTTAGAAGT AT
GTACGATAGATACAGAGATGTGTTCATAAACCATATTAATGATTACGTATTAGAAGATGA
TATAAAAATTATAATTTCAAAATACTATAAACTATTATTTGATTATAACTGCTTAG GGG
GTAAAAATAATAGAGGAATTTTAGTTATATTAATTTATGAGTATGTAAAGAATAGAGATA
TTAATTGTAATGAGTGGGAAAAAGTGGCTTGTATAG CATGGTGTATAGAAATTTTACAA
GCATCTTTTTTAGTAGCAGATGATATTATGGATAAGGGAGAAACACGCAGAAACAAACAT
TGTTG AGAAGCCACTTTAAAAACTATAGTAGGACAACATTTAGATACAAATATATTTTC
AGATAAATACTCCCATATAGACAAAGATATAGATGTTAATAATATTAATATATCTCAAGA
GAATAAAATTAATATAAACATGTTAAATTTTAAGGTTTATCAAAACATTATTATTCATAA
AACTGCTTATTATTCATTCTTTTTACCTATTGTTTGTG GTATGCAAATGGGGGGTATAT
CATTGGACAATTTATTATACAAAAAGGTCGAAAATATAGCAATTCTTATGGGGGAATATT
TTCAA GTCCATGATGATTATATAGATACCTTTGGAGATTCTAAAAAGACGGGAAAAGTT
GGCTCAGATATTCAAAATAATAAATTAACGTGGCCCTTGATAAAA GCATTTGAACTATG
TTCACAACCTGAAAAAGAGGACATAATAAGAAATTATGGGAAAGATAATGTAACATGTAT
TAAGTTTATTAATGATATATATGAACATTATAATATCAGGGATCATTATGTGGAATATGA
AAAGAAGCAGAAGATGAAAATATTAGA AGCCATAAACCAATTGCATCATGAAG GTATA
GAATATGTCTTGAAATACGTAATGGACATTTTGTTTACAGGCGCTTGA

>var147_padded
ATGGAGAACGAGCAGAATAACCAAGATTCAGAAAATGGTCTGGATTACTTTAGAAGT AT
GTACGATAGATACAGAGATGTGTTCATAAACCATATTAATGATTACGTATTAGAAGATGA
TATAAAAATTATAATTTCAAAATACTATAAACTATTATTTGATTATAACTGCTTAG GGG
GTAAAAATAATAGAGGAATTTTAGTTATATTAATTTATGAGTATGTAAAGAATAGAGATA
TTAATTGTAATGAGTGGGAAAAAGTGGCTTGTATAG AAATTTTACAAGCATCTTTTTTA
GTAGCAGATGATATTATGGATAAGGGAGAAACACGCAGAAACAAACATTGTTG GTATTT
GTTAAAAGACGTTGAAATTAAGAATGCGGTGAATGATGTGTTTCTTCTGTATAACGCTAT
ATACAA ATTACTTGATGTATATTTGCGCAATGATAACTGTTACCTTGATTTAATTACAT
CCTTTAGAGAAGCCACTTTAAAAACTATAGTAGGACAACATTTAGATACAAATATATTTT
CAGATAAATACTCCCATATAGACAAAGATATAGATGTTAATAATATTAATATATCTCAAG
AGAATAAAATTAATATAAACATGTTAAATTTTAAGGTTTATCAAAACATTATTATTCATA
AAACTGCTTATTATTCATTCTTTTTACCTATTGTTTGTG GTATGCAAATGGGGGGTATA
TCATTGGACAATTTATTATACAAAAAGGTCGAAAATATAGCAATTCTTATGGGGGAATAT
TTTCAA GTCCATGATGATTATATAGATACCTTTGGAGATTCTAAAAAGACGGGAAAAGT
TGGCTCAGATATTCAAAATAATAAATTAACGTGGCCCTTGATAAAA GCATTTGAACTAT
GTTCACAACCTGAAAAAGAGGACATAATAAGAAATTATGGGAAAGATAATGTAACATGTA
TTAAGTTTATTAATGATATATATGAACATTATAATATCAGGGATCATTATGTGGAATATG
AAAAGAAGCAGAAGATGAAAATATTAGA AGCCATAAACCAATTGCATCATGAAG GTAT
AGAATATGTCTTGAAATACGTAATGGACATTTTGTTTACAGGCGCTTGA

>var188_padded
ATGGAGAACGAGCAGAATAACCAAGATTCAGAAAATGGTCTGGATTACTTTAGAAGT AT

GTACGATAGATACAGAGATGTGTTCATAAACCATATTAATGATTACGTATTAGAAGATGA
TATAAAAATTATAATTTCAAAATACTATAAACTATTATTTGATTATAACTGCTTAG GGG
GTAAAAATAATAGAGGAATTTTAGTTATATTAATTTATGAGTATGTAAAGAATAGAGATA
TTAATTGTAATGAGTGGGAAAAAGTGGCTTGTATAG TAGCAGATGATATTATGGATAAG
GGAGAAACACGCAGAAACAAACATTGTTG GTATTTGTTAAAAGACGTTGAAATTAAGAA
TGCGGTGAATGATGTGTTTCTTCTGTATAACGCTATATACAA ATTACTTGATGTATATT
TGCGCAATGATAACTGTTACCTTGATTTAATTACATCCTTTAGAGAAGCCACTTTAAAAA
CTATAGTAGGACAACATTTAGATACAAATATATTTTCAGATAAATACTCCCATATAGACA
AAGATATAGATGTTAATAATATTAATATATCTCAAGAGAATAAAATTAATATAAACATGT
TAAATTTTAAGGTTTATCAAAACATTATTATTCATAAAACTGCTTATTATTCATTCTTTT
TACCTATTGTTT GTATGCAAATGGGGGGTATATCATTGGACAATTTATTATACAAAAAG
GTCGAAAATATAGCAATTCTTATGGGGGAATATTTTCAA GTCCATGATGATTATATAGA
TACCTTTGGAGATTCTAAAAAGACGGGAAAAGTTGGCTCAGATATTCAAAATAATAAATT
AACGTGGCCCTTGATAAAA GCATTTGAACTATGTTCACAACCTGAAAAAGAGGACATAA
TAAGAAATTATGGGAAAGATAATGTAACATGTATTAAGTTTATTAATGATATATATGAAC
ATTATAATATCAGGGATCATTATGTGGAATATGAAAAGAAGCAGAAGATGAAAATATTAG
A AGCCATAAACCAATTGCATCATGAAG GTATAGAATATGTCTTGAAATACGTAATGGA
CATTTTGTTTACAGGCGCTTGA

>var017_padded
ATGGAGAACGAGCAGAATAACCAAGATTCAGAAAATGGTCTGGATTACTTTAGAAGT AT
GTACGATAGATACAGAGATGTGTTCATAAACCATATTAATGATTACGTATTAGAAGATGA
TATAAAAATTATAATTTCAAAATACTATAAACTATTATTTGATTATAACTGCTTAG GGG
GTAAAAATAATAGAGGAATTTTAGTTATATTAATTTATGAGTATGTAAAGAATAGAGATA
TTAATTGTAATGAGTGGGAAAAAGTGGCTTGTATAG TAGCAGATGATATTATGGATAAG
GGAGAAACACGCAGAAACAAACATTGTTG GTATTTGTTAAAAGACGTTGAAATTAAGAA
TGCGGTGAATGATGTGTTTCTTCTGTATAACGCTATATACAA ATTACTTGATGTATATT
TGCGCAATGATAACTGTTACCTTGATTTAATTACATCCTTTAGAGAAGCCACTTTAAAAA
CTATAGTAGGACAACATTTAGATACAAATATATTTTCAGATAAATACTCCCATATAGACA
AAGATATAGATGTTAATAATATTAATATATCTCAAGAGAATAAAATTAATATAAACATGT
TAAATTTTAAGGTTTATCAAAACATTATTATTCATAAAACTGCTTATTATTCATTCTTTT
TACCTATTGTTTGTG GTATGCAAATGGGGGGTATATCATTGGACAATTTATTATACAAA
AAGGTCGAAAATATAGCAATTCTTATGGGGGAATATTTTCAA GTCCATGATGATTATAT
AGATACCTTTGGAGATTCTAAAAAGACGGGAAAAGTTGGCTCAGATATTCAAAATAATAA
ATTAACGTGGCCCTTGATAAAA GCATTTGAACTATGTTCACAACCTGAAAAAGAGGACA
TAATAAGAAATTATGGGAAAGATAATGTAACATGTATTAAGTTTATTAATGATATATATG
AACATTATAATATCAGGGATCATTATGTGGAATATGAAAAGAAGCAGAAGATGAAAATAT
TAGA AGCCATAAACCAATTGCATCATGAAG GTATAGAATATGTCTTGAAATACGTAAT
GGACATTTTGTTTACAGGCGCTTGA

>var189_padded
ATGGAGAACGAGCAGAATAACCAAGATTCAGAAAATGGTCTGGATTACTTTAGAAGT AT
GTACGATAGATACAGAGATGTGTTCATAAACCATATTAATGATTACGTATTAGAAGATGA
TATAAAAATTATAATTTCAAAATACTATAAACTATTATTTGATTATAACTGCTTAG GGG
GTAAAAATAATAGAGGAATTTTAGTTATATTAATTTATGAGTATGTAAAGAATAGAGATA
TTAATTGTAATGAGTGGGAAAAAGTGGCTTGTATAG CAGATGATATTATGGATAAGGGA
GAAACACGCAGAAACAAACATTGTTG GTATTTGTTAAAAGACGTTGAAATTAAGAATGC
GGTGAATGATGTGTTTCTTCTGTATAACGCTATATACAA ATTACTTGATGTATATTTGC
GCAATGATAACTGTTACCTTGATTTAATTACATCCTTTAGAGAAGCCACTTTAAAAACTA
TAGTAGGACAACATTTAGATACAAATATATTTTCAGATAAATACTCCCATATAGACAAAG
ATATAGATGTTAATAATATTAATATATCTCAAGAGAATAAAATTAATATAAACATGTTAA
ATTTTAAGGTTTATCAAAACATTATTATTCATAAAACTGCTTATTATTCATTCTTTTTAC
CTATTGTTTGTG GTATGCAAATGGGGGGTATATCATTGGACAATTTATTATACAAAAAG
GTCGAAAATATAGCAATTCTTATGGGGGAATATTTTCAA GTCCATGATGATTATATAGA
TACCTTTGGAGATTCTAAAAAGACGGGAAAAGTTGGCTCAGATATTCAAAATAATAAATT
AACGTGGCCCTTGATAAAA GCATTTGAACTATGTTCACAACCTGAAAAAGAGGACATAA
TAAGAAATTATGGGAAAGATAATGTAACATGTATTAAGTTTATTAATGATATATATGAAC
ATTATAATATCAGGGATCATTATGTGGAATATGAAAAGAAGCAGAAGATGAAAATATTAG
A AGCCATAAACCAATTGCATCATGAAG GTATAGAATATGTCTTGAAATACGTAATGGA
CATTTTGTTTACAGGCGCTTGA

>var116_padded
ATGGAGAACGAGCAGAATAACCAAGATTCAGAAAATGGTCTGGATTACTTTAGAAGT AT
GTACGATAGATACAGAGATGTGTTCATAAACCATATTAATGATTACGTATTAGAAGATGA

```
TATAAAAATTATAATTTCAAAATACTATAAACTATTATTTGATTATAACTGCTTAG TTA
TATTAATTTATGAGTATGTAAAGAATAGAGATATTAATTGTAATGAGTGGGAAAAAGTGG
CTTGTATAG CATGGTGTATAGAAATTTTACAAGCATCTTTTTTAGTAGCAGATGATATT
ATGGATAAGGGAGAAACACGCAGAAACAAACATTGTTG GTATTTGTTAAAAGACGTTGA
AATTAAGAATGCGGTGAATGATGTGTTTCTTCTGTATAACGCTATATACAA ATTACTTG
ATGTATATTTGCGCAATGATAACTGTTACCTTGATTTAATTACATCCTTTAGAGAAGCCA
CTTTAAAAACTATAGTAGGACAACATTTAGATACAAATATATTTTCAGATAAATACTCCC
ATATAGACAAAGATATAGATGTTAATAATATTAATATATCTCAAGAGAATAAAATTAATA
TAAACATGTTAAATTTTAAGGTTTATCAAAACATTATTATTCATAAAACTGCTTATTATT
CATTCTTTTTACCTATTGTTTGTG GTATGCAAATGGGGGGTATATCATTGGACAATTTA
TTATACAAAAAGGTCGAAAATATAGCAATTCTTATGGGGGAATATTTTCAA GTCCATGA
TGATTATATAGATACCTTTGGAGATTCTAAAAAGACGGGAAAAGTTGGCTCAGATATTCA
AAATAATAAATTAACGTGGCCCTTGATAAAA GCATTTGAACTATGTTCACAACCTGAAA
AAGAGGACATAATAAGAAATTATGGGAAAGATAATGTAACATGTATTAAGTTTATTAATG
ATATATATGAACATTATAATATCAGGGATCATTATGTGGAATATGAAAAGAAGCAGAAGA
TGAAAATATTAGA AGCCATAAACCAATTGCATCATGAAG GTATAGAATATGTCTTGAA
ATACGTAATGGACATTTTGTTTACAGGCGCTTGA

>var218_padded
ATGGAGAACGAGCAGAATAACCAAGATTCAGAAAATGGTCTGGATTACTTTAGAAGT AT
GTACGATAGATACAGAGATGTGTTCATAAACCATATTAATGATTACGTATTAGAAGATGA
TATAAAAATTATAATTTCAAAATACTATAAACTATTATTTGATTATAACTGCTTAGGTAA
GATAA GGGGTAAAAATAATAGAGGAATTTTAGTTATATTAATTTATGAGTATGTAAAGA
ATAGAGATATTAATTGTAATGAGTGGGAAAAAGTGGCTTGTATAG CATGGTGTATAGAA
ATTTTACAAGCATCTTTTTTAGTAGCAGATGATATTATGGATAAGGGAGAAACACGCAGA
AACAAACATTGTTG GTATTTGTTAAAAGACGTTGAAATTAAGAATGCGGTGAATGATGT
GTTTCTTCTGTATAACGCTATATACAA ATTACTTGATGTATATTTGCGCAATGATAACT
GTTACCTTGATTTAATTACATCCTTTAGAGAAGCCACTTTAAAAACTATAGTAGGACAAC
ATTTAGATACAAATATATTTTCAGATAAATACTCCCATATAGACAAAGATATAGATGTTA
ATAATATTAATATATCTCAAGAGAATAAAATTAATATAAACATGTTAAATTTTAAGGTTT
ATCAAAACATTATTATTCATAAAACTGCTTATTATTCATTCTTTTTACCTATTGTTTGTG
 GTATGCAAATGGGGGGTATATCATTGGACAATTTATTATACAAAAAGGTCGAAAATATA
GCAATTCTTATGGGGGAATATTTTCAA GTCCATGATGATTATATAGATACCTTTGGAGA
TTCTAAAAAGACGGGAAAAGTTGGCTCAGATATTCAAAATAATAAATTAACGTGGCCCTT
GATAAAA GCATTTGAACTATGTTCACAACCTGAAAAAGAGGACATAATAAGAAATTATG
GGAAAGATAATGTAACATGTATTAAGTTTATTAATGATATATATGAACATTATAATATCA
GGGATCATTATGTGGAATATGAAAAGAAGCAGAAGATGAAAATATTAGA AGCCATAAAC
CAATTGCATCATGAAG GTATAGAATATGTCTTGAAATACGTAATGGACATTTTGTTTAC
AGGCGCTTGA

>var115_padded
ATGGAGAACGAGCAGAATAACCAAGATTCAGAAAATGGTCTGGATTACTTTAGAAGT AT
GTACGATAGATACAGAGATGTGTTCATAAACCATATTAATGATTACGTATTAGAAGATGA
TATAAAAATTATAATTTCAAAATACTATAAACTATTATTTGATTATAACTGCTTAG GGG
GTAAAAATAATAGAGGAATTTTAGTTATATTAATTTATGAGTATGTAAAGAATAGAGATA
TTAATTGTAATGAGTGGGAAAAAGTGGCTTGTATAG CATGGTGTATAGAAATTTTACAA
GCATCTTTTTTAGTAGCAGATGATATTATGGATAAGGGAGAAACACGCAGAAACAAACAT
TGTTG GTATTTGTTAAAAGACGTTGAAATTAAGAATGCGGTGAATGATGTGTTTCTTCT
 ATTACTTGATGTATATTTGCGCAATGATAACTGTTACCTTGATTTAATTACATCCTTTA
GAGAAGCCACTTTAAAAACTATAGTAGGACAACATTTAGATACAAATATATTTTCAGATA
AATACTCCCATATAGACAAAGATATAGATGTTAATAATATTAATATATCTCAAGAGAATA
AAATTAATATAAACATGTTAAATTTTAAGGTTTATCAAAACATTATTATTCATAAAACTG
CTTATTATTCATTCTTTTTACCTATTGTTTGTG GTATGCAAATGGGGGGTATATCATTG
GACAATTTATTATACAAAAAGGTCGAAAATATAGCAATTCTTATGGGGGAATATTTTCAA
 GTCCATGATGATTATATAGATACCTTTGGAGATTCTAAAAAGACGGGAAAAGTTGGCTC
AGATATTCAAAATAATAAATTAACGTGGCCCTTGATAAAA GCATTTGAACTATGTTCAC
AACCTGAAAAAGAGGACATAATAAGAAATTATGGGAAAGATAATGTAACATGTATTAAGT
TTATTAATGATATATATGAACATTATAATATCAGGGATCATTATGTGGAATATGAAAAGA
AGCAGAAGATGAAAATATTAGA AGCCATAAACCAATTGCATCATGAAG GTATAGAATA
TGTCTTGAAATACGTAATGGACATTTTGTTTACAGGCGCTTGA

>var062_padded
ATGGAGAACGAGCAGAATAACCAAGATTCAGAAAATGGTCTGGATTACTTTAGAAGT AT
GTACGATAGATACAGAGATGTGTTCATAAACCATATTAATGATTACGTATTAGAAGATGA
```

TATAAAAATTATAATTTCAAAATACTATAAACTATTATTTGATTATAACTGCTTAG GGG
GTAAAAATAATAGAGGAATTTTAGTTATATTAATTTATGAGTATGTAAAGAATAGAGATA
TTAATTGTAATGAGTGGGAAAAAGTGGCTTGTATAG CATGGTGTATAGAAATTTTACAA
GCATCTTTTTTAGTAGCAGATGATATTATGGATAAGGGAGAAACACGCAGAAACAAACAT
TGTTG GTATTTGTTAAAAGACGTTGAAATTAAGAATGCGGTGAATGATGTGTTTCTTCT
GTATAACGCTATATACAA ATTACTTGATGTATATTTGCGCAATGATAACTGTTACCTTG
ATTTAATTACATCCTTTAGAGAAGCCACTTTAAAAACTATAGTAGGACAACATTTAGATA
CAAATATATTTTCAGATAAATACTCCCATATAGACAAAGATATAGATGTTAATAATATTA
ATATATCTCAAGAGAATAAAATTAATATAAACATGTTAAATTTTAAGGTTTATCAAAACA
TTATTATTCATAAAACTGCTTATTATTCATTCTTTTTACCTATTGTTTGTG GTATGCAA
ATGGGGGGTATATCATTGGACAATTTATTATACAAAAAGGTCGAAAATATAGCAATTCTT
ATGGGGGAATATTTTCAA GTCCATGATGATTATATAGATACCTTTGGAGATTCTAAAAA
GACGGGAAAA GCATTTGAACTATGTTCACAACCTGAAAAAGAGGACATAATAAGAAATT
ATGGGAAAGATAATGTAACATGTATTAAGTTTATTAATGATATATATGAACATTATAATA
TCAGGGATCATTATGTGGAATATGAAAAGAAGCAGAAGATGAAAATATTAGA AGCCATA
AACCAATTGCATCATGAAG GTATAGAATATGTCTTGAAATACGTAATGGACATTTTGTT
TACAGGCGCTTGA

>var310_padded
ATGGTCTGGATTACTTTAGAAGT ATA
CAGAGATGTGTTCATAAACCATATTAATGATTACGTATTAGAAGATGATATAAAAATTAT
AATTTCAAAATACTATAAACTATTATTTGATTATAACTGCTTAG GGGGTAAAAATAATA
GAGGAATTTTAGTTATATTAATTTATGAGTATGTAAAGAATAGAGATATTAATTGTAATG
AGTGGGAAAAAGTGGCTTGTATAG CATGGTGTATAGAAATTTTACAAGCATCTTTTTTA
GTAGCAGATGATATTATGGATAAGGGAGAAACACGCAGAAACAAACATTGTTG GTATTT
GTTAAAAGACGTTGAAATTAAGAATGCGGTGAATGATGTGTTTCTTCTGTATAACGCTAT
ATACAA ATTACTTGATGTATATTTGCGCAATGATAACTGTTACCTTGATTTAATTACAT
CCTTTAGAGAAGCCACTTTAAAAACTATAGTAGGACAACATTTAGATACAAATATATTTT
CAGATAAATACTCCCATATAGACAAAGATATAGATGTTAATAATATTAATATATCTCAAG
AGAATAAAATTAATATAAACATGTTAAATTTTAAGGTTTATCAAAACATTATTATTCATA
AAACTGCTTATTATTCATTCTTTTTACCTATTGTTT GTATGCAAATGGGGGGTATATCA
TTGGACAATTTATTATACAAAAAGGTCGAAAATATAGCAATTCTTATGGGGGAATATTTT
CAA GTCCATGATGATTATATAGATACCTTTGGAGATTCTAAAAAGACGGGAAAAGTTGG
CTCAGATATTCAAAATAATAAATTAACGTGGCCCTTGATAAAA GCATTTGAACTATGTT
CACAACCTGAAAAAGAGGACATAATAAGAAATTATGGGAAAGATAATGTAACATGTATTA
AGTTTATTAATGATATATATGAACATTATAATATCAGGGATCATTATGTGGAATATGAAA
AGAAGCAGAAGATGAAAATATTAGA AGCCATAAACCAATTGCATCATGAAG GTATAGA
ATATGTCTTGAAATACGTAATGGACATTTTGTTTACAGGCGCTTGA

>var311_padded
ATGGTCTGGATTACTTTAGAAGT ATA
CAGAGATGTGTTCATAAACCATATTAATGATTACGTATTAGAAGATGATATAAAAATTAT
AATTTCAAAATACTATAAACTATTATTTGATTATAACTGCTTAG GGGGTAAAAATAATA
GAGGAATTTTAGTTATATTAATTTATGAGTATGTAAAGAATAGAGATATTAATTGTAATG
AGTGGGAAAAAGTGGCTTGTATAG CATGGTGTATAGAAATTTTACAAGCATCTTTTTTA
GTAGCAGATGATATTATGGATAAGGGAGAAACACGCAGAAACAAACATTGTTG GTATTT
GTTAAAAGACGTTGAAATTAAGAATGCGGTGAATGATGTGTTTCTTCTGTATAACGCTAT
ATACAA ATTACTTGATGTATATTTGCGCAATGATAACTGTTACCTTGATTTAATTACAT
CCTTTAGAGAAGCCACTTTAAAAACTATAGTAGGACAACATTTAGATACAAATATATTTT
CAGATAAATACTCCCATATAGACAAAGATATAGATGTTAATAATATTAATATATCTCAAG
AGAATAAAATTAATATAAACATGTTAAATTTTAAGGTTTATCAAAACATTATTATTCATA
AAACTGCTTATTATTCATTCTTTTTACCTATTGTTTGTG GTATGCAAATGGGGGGTATA
TCATTGGACAATTTATTATACAAAAAGGTCGAAAATATAGCAATTCTTATGGGGGAATAT
TTTCAA GTCCATGATGATTATATAGATACCTTTGGAGATTCTAAAAAGACGGGAAAAGT
TGGCTCAGATATTCAAAATAATAAATTAACGTGGCCCTTGATAAAA GCATTTGAACTAT
GTTCACAACCTGAAAAAGAGGACATAATAAGAAATTATGGGAAAGATAATGTAACATGTA
TTAAGTTTATTAATGATATATATGAACATTATAATATCAGGGATCATTATGTGGAATATG
AAAAGAAGCAGAAGATGAAAATATTAGA AGCCATAAACCAATTGCATCATGAAG GTAT
AGAATATGTCTTGAAATACGTAATGGACATTTTGTTTACAGGCGCTTGA

>var025_padded
ATGGAGAACGAGCAGAATAACCAAGATTCAGAAAATGGTCTGGATTACTTTAGAAGT AT
GTACGATAGATACAGAGATGTGTTCATAAACCATATTAATGATTACGTATTAGAAGATGA
TATAAAAATTATAATTTCAAAATACTATAAACTATTATTTGATTATAACTGCTTAG GGG

GTAAAAATAATAGAGGAATTTTAGTTATATTAATTTATGAGTATGTAAAGAATAGAGATA
TTAATTGTAATGAGTGGGAAAAAGTGGCTTGTATAG CATGGTGTATAGAAATTTTACAA
GCATCTTTTTTAGTAGCAGATGATATTATGGATAAGGGAGAAACACGCAGAAACAAACAT
TGTTG GTATTTGTTAAAAGACGTTGAAATTAAGAATGCGGTGAATGATGTGTTTCTTCT
GTATAACGCTATATACAA ATTACTTGATGTATATTTGCGCAATGATAACTGTTACCTTG
ATTTAATTACATCCTTTAGAGAAGCCACTTTAAAAACTATAGTAGGACAACATTTAGATA
CAAATATATTTTCAGATAAATACTCCCATATAGACAAAGATATAGATGTTAATAATATTA
ATATATCTCAAGAGA TAAATTTTAAGGTTTATCAAAACATTATTATTCATAAAACTGCT
TATTATTCATTCTTTTTACCTATTGTTT GTATGCAAATGGGGGGTATATCATTGGACAA
TTTATTATACAAAAAGGTCGAAAATATAGCAATTCTTATGGGGGAATATTTTCAA GTCC
ATGATGATTATATAGATACCTTTGGAGATTCTAAAAAGACGGGAAAAGTTGGCTCAGATA
TTCAAAATAATAAATTAACGTGGCCCTTGATAAAA GCATTTGAACTATGTTCACAACCT
GAAAAAGAGGACATAATAAGAAATTATGGGAAAGATAATGTAACATGTATTAAGTTTATT
AATGATATATATGAACATTATAATATCAGGGATCATTATGTGGAATATGAAAAGAAGCAG
AAGATGAAAATATTAGA AGCCATAAACCAATTGCATCATGAAG GTATAGAATATGTCT
TGAAATACGTAATGGACATTTTGTTTACAGGCGCTTGA

>var267_padded
ATGGAGAACGAGCAGAATAACCAAGATTCAGAAAATGGTCTGGATTACTTTAGAAGT AT
GTACGATAGATACAGAGATGTGTTCATAAACCATATTAATGATTACGTATTAGAAGATGA
TATAAAAATTATAATTTCAAAATACTATAAACTATTATTTGATTATAACTGCTTAG GGG
GTAAAAATAATAGAGGAATTTTAGTTATATTAATTTATGAGTATGTAAAGAATAGAGATA
TTAATTGTAATGAGTGGGAAAAAGTGGCTTGTATAG CATGGTGTATAGAAATTTTACAA
GCATCTTTTTTAGTAGCAGATGATATTATGGATAAGGGAGAAACACGCAGAAACAAACAT
TGTTG GTATTTGTTAAAAGACGTTGAAATTAAGAATGCGGTGAATGATGTGTTTCTTCT
GTATAACGCTATATACAA ATTACTTGATGTATATTTGCGCAATGATAACTGTTACCTTG
ATTTAATTACATCCTTTAGAGAAGCCACTTTAAAAACTATAGTAGGACAACATTTAGATA
CAAATATATTTTCAGATAAATACTCCCATATAGACAAAGATATAGATGTTAATAATATTA
ATATATCTCAAGAGAATAAAATTAATATAAACATGTTAAATTTTAAGGTTTATCAAAACA
TTATTATTCATAAAACTGCT TATTTATTCATTTTAGGTATGCAAATGGGGGGTATATCA
TTGGACAATTTATTATACAAAAAGGTCGAAAATATAGCAATTCTTATGGGGGAATATTTT
CAA GTCCATGATGATTATATAGATACCTTTGGAGATTCTAAAAAGACGGGAAAAGTTGG
CTCAGATATTCAAAATAATAAATTAACGTGGCCCTTGATAAAA GCATTTGAACTATGTT
CACAACCTGAAAAAGAGGACATAATAAGAAATTATGGGAAAGATAATGTAACATGTATTA
AGTTTATTAATGATATATATGAACATTATAATATCAGGGATCATTATGTGGAATATGAAA
AGAAGCAGAAGATGAAAATATTAGA AGCCATAAACCAATTGCATCATGAAG GTATAGA
ATATGTCTTGAAATACGTAATGGACATTTTGTTTACAGGCGCTTGA

>var235_padded
ATGGAGAACGAGCAGAATAACCAAGATTCAGAAAATGGTCTGGATTACTTTAGAAGT AT
GTACGATAGATACAGAGATGTGTTCATAAACCATATTAATGATTACGTATTAGAAGATGA
TATAAAAATTATAATTTCAAAATACTATAAACTATTATTTGATTATAACTGCTTAG GGG
GTAAAAATAATAGAGGAATTTTAGTTATATTAATTTATGAGTATGTAAAGAATAGAGATA
TTAATTGTAATGAGTGGGAAAAAGTGGCTTGTATAG CATGGTGTATAGAAATTTTACAA
GCATCTTTTTTAGTAGCAGATGATATTATGGATAAGGGAGAAACACGCAGAAACAAACAT
TGTTG GTATTTGTTAAAAGACGTTGAAATTAAGAATGCGGTGAATGATGTGTTTCTTCT
GTATAACGCTATATACAA ATTACTTGATGTATATTTGCGCAATGATAACTGTTACCTTG
ATTTAATTACATCCTTTAGAGAAGCCACTTTAAAAACTATAGTAGGACAACATTTAGATA
CAAATATATTTTCAGATAAATACTCCCATATAGACAAAGATATAGATGTTAATAATATTA
ATATATCTCAAGAGAATAAAATTAATATAAACATGTTAAATTTTAAGGTTTATCAAAACA
TTATTATTCATAAAACTGCTTATTATTCATTCTTTTTACCTATTGTTTGTG GTATGCAA
ATGGGGGGTATATCATTGGACAATTTATTATACAAAAAGGTCGAAAATATAGCAATTCTT
ATGGGGGAATATTTTCAA GTCCATGATGATTATATAGATACCTTTGGAGATTCTAAAAA
GACGGGAAAAGTTGGCTCAGATATTCAAAATAATAAATTAACGTGGCCCTTGATAAAA T
AGGCATTTGAACTATGTTCACAACCTGAAAAAGAGGACATAATAAGAAATTATGGGAAAG
ATAATGTAACATGTATTAAGTTTATTAATGATATATATGAACATTATAATATCAGGGATC
ATTATGTGGAATATGAAAAGAAGCAGAAGATGAAAATATTAGA AGCCATAAACCAATTG
CATCATGAAG GTATAGAATATGTCTTGAAATACGTAATGGACATTTTGTTTACAGGCGC
TTGA

>var001_padded
ATG ATGTACGATAGATACAGAGATGT
GTTCATAAACCATATTAATGATTACGTATTAGAAGATGATATAAAAATTATAATTTCAAA
ATACTATAAACTATTATTTGATTATAACTGCTTAG GGGGTAAAAATAATAGAGGAATTT

TAGTTATATTAATTTATGAGTATGTAAAGAATAGAGATATTAATTGTAATGAGTGGGAAA
AAGTGGCTTGTATAG CATGGTGTATAGAAATTTTACAAGCATCTTTTTTAGTAGCAGAT
GATATTATGGATAAGGGAGAAACACGCAGAAACAAACATTGTTG GTATTTGTTAAAAGA
CGTTGAAATTAAGAATGCGGTGAATGATGTGTTTCTTCTGTATAACGCTATATACAA AT
TACTTGATGTATATTTGCGCAATGATAACTGTTACCTTGATTTAATTACATCCTTTAGAG
AAGCCACTTTAAAAACTATAGTAGGACAACATTTAGATACAAATATATTTTCAGATAAAT
ACTCCCATATAGACAAAGATATAGATGTTAATAATATTAATATATCTCAAGAGAATAAAA
TTAATATAAACATGTTAAATTTTAAGGTTTATCAAAACATTATTATTCATAAAACTGCTT
ATTATTCATTCTTTTTACCTATTGTTTGTG GTATGCAAATGGGGGGTATATCATTGGAC
AATTTATTATACAAAAAGGTCGAAAATATAGCAATTCTTATGGGGGAATATTTTCAA GT
CCATGATGATTATATAGATACCTTTGGAGATTCTAAAAAGACGGGAAAAGTTGGCTCAGA
TATTCAAAATAATAAATTAACGTGGCCCTTGATAAAA GCATTTGAACTATGTTCACAAC
CTGAAAAAGAGGACATAATAAGAAATTATGGGAAAGATAATGTAACATGTATTAAGTTTA
TTAATGATATATATGAACATTATAATATCAGGGATCATTATGTGGAATATGAAAAGAAGC
AGAAGATGAAAATATTAGA AGCCATAAACCAATTGCATCATGAAG GTATAGAATATGT
CTTGAAATACGTAATGGACATTTTGTTTACAGGCGCTTGA

>var024_padded
ATGGAGAACGAGCAGAATAACCAAGATTCAGAAAATGGTCTGGATTACTTTAGAAGT AT
GTACGATAGATACAGAGATGTGTTCATAAACCATATTAATGATTACGTATTAGAAGATGA
TATAAAAATTATAATTTCAAAATACTATAAACTATTATTTGATTATAACTGCTTAG GGG
GTAAAAATAATAGAGGAATTTTAGTTATATTAATTTATGAGTATGTAAAGAATAGAGATA
TTAATTGTAATGAGTGGGAAAAAGTGGCTTGTATAG CATGGTGTATAGAAATTTTACAA
GCATCTTTTTTAGTAGCAGATGATATTATGGATAAGGGAGAAACACGCAGAAACAAACAT
TGTTG GTATTTGTTAAAAGACGTTGAAATTAAGAATGCGGTGAATGATGTGTTTCTTCT
GTATAACGCTATATACAA ATTACTTGATGTATATTTGCGCAATGATAACTGTTACCTTG
ATTTAATTACATCCTTTAGAGAAGCCACTTTAAAAACTATAGTAGGACAACATTTAGATA
CAAATATATTTTCAGATAAATACTCCCATATAGACAAAGATATAGATGTTAATAATATTA
ATATATCTCAAGAGAATAAAATTAATATAAACATGTTAAATTTTAAGGTTTATCAAAACA
TTATTATTCATAAAACTGCTTATTATTCATTCTTTTTACCTATTGTTTGTG GTATGCAA
ATGGGGGGTATATCATTGGACAATTTATTATACAAAAAGGTCGAAAATATAGCAATTCTT
ATGGGGGAATATTTTCAA GTCCATGATGATTATATAGATACCTTTGGAGATTCTAAAAA
GACGGGAAAAGTTGGCTCAGATATTCAAAATAATAAATTAACGTGGCCCTTGATAAAA A
TTATTATTATTATTTTTTTTTTTTTTGTAGGCATTTGAACTATGTTCACAACCTGAAAAAG
AGGACATAATAAGAAATTATGGGAAAGATAATGTAACATGTATTAAGTTTATTAATGATA
TATATGAACATTATAATATCAGGGATCATTATGTGGAATATGAAAAGAAGCAGAAGATGA
AAATATTAGA AGCCATAAACCAATTGCATCATGAAG GTATAGAATATGTCTTGAAATA
CGTAATGGACATTTTGTTTACAGGCGCTTGA

>var230_padded
ATGGAGAACGAGCAGAATAACCAAGATTCAGAAAATGGTCTGGATTACTTTAGAAGT AT
GTACGATAGATACAGAGATGTGTTCATAAACCATATTAATGATTACGTATTAGAAGATGA
TATAAAAATTATAATTTCAAAATACTATAAACTATTATTTGATTATAACTGCTTAG GGG
GTAAAAATAATAGAGGAATTTTAGTTATATTAATTTATGAGTATGTAAAGAATAGAGATA
TTAATTGTAATGAGTGGGAAAAAGTGGCTTGTATAG CATGGTGTATAGAAATTTTACAA
GCATCTTTTTTAGTAGCAGATGATATTATGGATAAGGGAGAAACACGCAGAAACAAACAT
TGTTG GTATTTGTTAAAAGACGTTGAAATTAAGAATGCGGTGAATGATGTGTTTCTTCT
GTATAACGCTATATACAA ATTACTTGATGTATATTTGCGCAATGATAACTGTTACCTTG
ATTTAATTACATCCTTTAGAGAAGCCACTTTAAAAACTATAGTAGGACAACATTTAGATA
CAAATATATTTTCAGATAAATACTCCCATATAGACAAAGATATAGATGTTAATAATATTA
ATATATCTCAAGAGAATAAAATTAATATAAACATGTTAAATTTTAAGGTTTATCAAAACA
TTATTATTCATAAAACTGCTTATTATTCATTCTTTTTACCTATTGTTTGTG GTATGCAA
ATGGGGGGTATATCATTGGACAATTTATTATACAAAAAGGTCGAAAATATAGCAATTCTT
ATGGGGGAATATTTTCAA GTCCATGATGATTATATAGATACCTTTGGAGATTCTAAAAA
GACGGGAAAAGTTGGCTCAGATATTCAAAATAATAAATTAACGTGGCCCTTGATAAAA G
CATTTGAACTATGTTCACAACCTGAAAAAGAGGACATAATAAGAAATTATGGGAAAGATA
ATGTAACATGTATTAAGTTTATTAATGATATATATGAACATTATAATATCAGGGATCATT
ATGTGGAATATGAAAAGAAGCAGAAGATGAAAATATTAGA CCATAAACCAATTGCATCA
TGAAG GTATAGAATATGTCTTGAAATACGTAATGGACATTTTGTTTACAGGCGCTTGAG
AGATAACAACCTTAACAACTTTTGTTTAAATATGATCAAGTTTAAAAAAAAAAAAAAATA
A

>var339_padded
ATGGAGAACGAGCAGAATAACCAAGATTCAGAAAATGGTCTGGATTACTTTAGAAGT AT

GTACGATAGATACAGAGATGTGTTCATAAACCATATTAATGATTACGTATTAGAAGATGA
TATAAAAATTATAATTTCAAAATACTATAAACTATTATTTGATTATAACTGCTTAG GGG
GTAAAAATAATAGAGGAATTTTAGTTATATTAATTTATGAGTATGTAAAGAATAGAGATA
TTAATTGTAATGAGTGGGAAAAAGTGGCTTGTATAG CATGGTGTATAGAAATTTTACAA
GCATCTTTTTTAGTAGCAGATGATATTATGGATAAGGGAGAAACACGCAGAAACAAACAT
TGTTG GTATTTGTTAAAAGACGTTGAAATTAAGAATGCGGTGAATGATGTGTTTCTTCT
GTATAACGCTATATACAA ATTACTTGATGTATATTTGCGCAATGATAACTGTTACCTTG
ATTTAATTACATCCTTTAGAGAAGCCACTTTAAAAACTATAGTAGGACAACATTTAGATA
CAAATATATTTTCAGATAAATACTCCCATATAGACAAAGATATAGATGTTAATAATATTA
ATATATCTCAAGAGAATAAAATTAATATAAACATGTTAAATTTTAAGGTTTATCAAAACA
TTATTATTCATAAAACTGCTTATTATTCATTCTTTTTACCTATTGTTTGTG GTATGCAA
ATGGGGGGTATATCATTGGACAATTTATTATACAAAAAGGTCGAAAATATAGCAATTCTT
ATGGGGGAATATTTTCAA GTCCATGATGATTATATAGATACCTTTGGAGATTCTAAAAA
GACGGGAAAAGTTGGCTCAGATATTCAAAATAATAAATTAACGTGGCCCTTGATAAAA G
CATTTGAACTATGTTCACAACCTGAAAAAGAGGACATAATAAGAAATTATGGGAAAGATA
ATGTAACATGTATTAAGTTTATTAATGATATATATGAACATTATAATATCAGGGATCATT
ATGTGGAATATGAAAAGAAGCAGAAGATGAAAATATTAGAGTAA

>var357_padded
TATATTTTTATATATGAAG GGGGTAAAAATAATAGAGGAATTTTAGTTATATTAATTTA
TGAGTATGTAAAGAATAGAGATATTAATTGTAATGAGTGGGAAAAAGTGGCTTGTATAG
CATGGTGTATAGAAATTTTACAAGCATCTTTTTTAGTAGCAGATGATATTATGGATAAGG
GAGAAACACGCAGAAACAAACATTGTTG GTATTTGTTAAAAGACGTTGAAATTAAGAAT
GCGGTGAATGATGTGTTTCTTCTGTATAACGCTATATACAA ATTACTTGATGTATATTT
GCGCAATGATAACTGTTACCTTGATTTAATTACATCCTTTAGAGAAGCCACTTTAAAAAC
TATAGTAGGACAACATTTAGATACAAATATATTTTCAGATAAATACTCCCATATAGACAA
AGATATAGATGTTAATAATATTAATATATCTCAAGAGAATAAAATTAATATAAACATGTT
AAATTTTAAGGTTTATCAAAACATTATTATTCATAAAACTGCTTATTATTCATTCTTTTT
ACCTATTGTTTGTG GTATGCAAATGGGGGGTATATCATTGGACAATTTATTATACAAAA
AGGTCGAAAATATAGCAATTCTTATGGGGGAATATTTTCAA GTCCATGATGATTATATA
GATACCTTTGGAGATTCTAAAAAGACGGGAAAAGTTGGCTCAGATATTCAAAATAATAAA
TTAACGTGGCCCTTGATAAAA GCATTTGAACTATGTTCACAACCTGAAAAAGAGGACAT
AATAAGAAATTATGGGAAAGATAATGTAACATGTATTAAGTTTATTAATGATATATATGA
ACATTATAATATCAGGGATCATTATGTGGAATATGAAAAGAAGCAGAAGATGAAAATATT
AGA AGCCATAAACCAATTGCATCATGAAG GTATAGAATATGTCTTGAAATACGTAATG
GACATTTTGTTTACAGGCGCTTGA

>var071_padded
ATGGAGAACGAGCAGAATAACCAAGATTCAGAAAATGGTCTGGATTACTTTAGAA GGGG
TAAAAATAATAGAGGAATTTTAGTTATATTAATTTATGAGTATGTAAAGAATAGAGATAT
TAATTGTAATGAGTGGGAAAAAGTGGCTTGTATAG CATGGTGTATAGAAATTTTACAAG
CATCTTTTTTAGTAGCAGATGATATTATGGATAAGGGAGAAACACGCAGAAACAAACATT
GTTG GTATTTGTTAAAAGACGTTGAAATTAAGAATGCGGTGAATGATGTGTTTCTTCTG
TATAACGCTATATACAA ATTACTTGATGTATATTTGCGCAATGATAACTGTTACCTTGA
TTTAATTACATCCTTTAGAGAAGCCACTTTAAAAACTATAGTAGGACAACATTTAGATAC
AAATATATTTTCAGATAAATACTCCCATATAGACAAAGATATAGATGTTAATAATATTAA
TATATCTCAAGAGAATAAAATTAATATAAACATGTTAAATTTTAAGGTTTATCAAAACAT
TATTATTCATAAAACTGCTTATTATTCATTCTTTTTACCTATTGTTTGTG GTATGCAAA
TGGGGGGTATATCATTGGACAATTTATTATACAAAAAGGTCGAAAATATAGCAATTCTTA
TGGGGGAATATTTTCAA GTCCATGATGATTATATAGATACCTTTGGAGATTCTAAAAAG
ACGGGAAAAGTTGGCTCAGATATTCAAAATAATAAATTAACGTGGCCCTTGATAAAA GC
ATTTGAACTATGTTCACAACCTGAAAAAGAGGACATAATAAGAAATTATGGGAAAGATAA
TGTAACATGTATTAAGTTTATTAATGATATATATGAACATTATAATATCAGGGATCATTA
TGTGGAATATGAAAAGAAGCAGAAGATGAAAATATTAGA AGCCATAAACCAATTGCATC
ATGAAG GTATAGAATATGTCTTGAAATACGTAATGGACATTTTGTTTACAGGCGCTTGA

>var006_padded
ATGGAGAACGAGCAGAATAACCAAGATTCAGAAAATGGTCTGGATTACTTTAGAAGT AT
GTACGATAGATACAGAGATGTGTTCATAAACCATATTAATGATTACGTATTAGAAGATGA
TATAAAAATTATAATTTCAAAATACTATAAACTATTATTTGATTATAACTGCTTAG ATG
TCTTATTAGGGGGTAAAAATAATAGAGGAATTTTAGTTATATTAATTTATGAGTATGTAA
AGAATAGAGATATTAATTGTAATGAGTGGGAAAAAGTGGCTTGTATAG CATGGTGTATA
GAAATTTTACAAGCATCTTTTTTAGTAGCAGATGATATTATGGATAAGGGAGAAACACGC
AGAAACAAACATTGTTG GTATTTGTTAAAAGACGTTGAAATTAAGAATGCGGTGAATGA

TGTGTTTCTTCTGTATAACGCTATATACAA ATTACTTGATGTATATTTGCGCAATGATA
ACTGTTACCTTGATTTAATTACATCCTTTAGAGAAGCCACTTTAAAAACTATAGTAGGAC
AACATTTAGATACAAATATATTTTCAGATAAATACTCCCATATAGACAAAGATATAGATG
TTAATAATATTAATATATCTCAAGAGAATAAAATTAATATAAACATGTTAAATTTTAAGG
TTTATCAAAACATTATTATTCATAAAACTGCTTATTATTCATTCTTTTTACCTATTGTTT
GTG GTATGCAAATGGGGGGTATATCATTGGACAATTTATTATACAAAAAGGTCGAAAAT
ATAGCAATTCTTATGGGGGAATATTTTCAA GTCCATGATGATTATATAGATACCTTTGG
AGATTCTAAAAAGACGGGAAAAGTTGGCTCAGATATTCAAAATAATAAATTAACGTGGCC
CTTGATAAAA GCATTTGAACTATGTTCACAACCTGAAAAAGAGGACATAATAAGAAATT
ATGGGAAAGATAATGTAACATGTATTAAGTTTATTAATGATATATATGAACATTATAATA
TCAGGGATCATTATGTGGAATATGAAAAGAAGCAGAAGATGAAAATATTAGA AGCCATA
AACCAATTGCATCATGAAG GTATAGAATATGTCTTGAAATACGTAATGGACATTTTGTT
TACAGGCGCTTGA

>var021_padded
ATGGAGAACGAGCAGAATAACCAAGATTCAGAAAATGGTCTGGATTACTTTAGAAGT AT
GTACGATAGATACAGAGATGTGTTCATAAACCATATTAATGATTACGTATTAGAAGATGA
TATAAAAATTATAATTTCAAAATACTATAAACTATTATTTGATTATAACTGCTTAG GGG
 CATGGTGTATAGAAATTTTACAAGCATCTTTTTTAGTAGCAGATGATATTATGGATAAG
GGAGAAACACGCAGAAACAAACATTGTTG GTATTTGTTAAAAGACGTTGAAATTAAGAA
TGCGGTGAATGATGTGTTTCTTCTGTATAACGCTATATACAA ATTACTTGATGTATATT
TGCGCAATGATAACTGTTACCTTGATTTAATTACATCCTTTAGAGAAGCCACTTTAAAAA
CTATAGTAGGACAACATTTAGATACAAATATATTTTCAGATAAATACTCCCATATAGACA
AAGATATAGATGTTAATAATATTAATATATCTCAAGAGAATAAAATTAATATAAACATGT
TAAATTTTAAGGTTTATCAAAACATTATTATTCATAAAACTGCTTATTATTCATTCTTTT
TACCTATTGTTTGTG GTATGCAAATGGGGGGTATATCATTGGACAATTTATTATACAAA
AAGGTCGAAAATATAGCAATTCTTATGGGGGAATATTTTCAA GTCCATGATGATTATAT
AGATACCTTTGGAGATTCTAAAAAGACGGGAAAAGTTGGCTCAGATATTCAAAATAATAA
ATTAACGTGGCCCTTGATAAAA GCATTTGAACTATGTTCACAACCTGAAAAAGAGGACA
TAATAAGAAATTATGGGAAAGATAATGTAACATGTATTAAGTTTATTAATGATATATATG
AACATTATAATATCAGGGATCATTATGTGGAATATGAAAAGAAGCAGAAGATGAAAATAT
TAGA AGCCATAAACCAATTGCATCATGAAG GTATAGAATATGTCTTGAAATACGTAAT
GGACATTTTGTTTACAGGCGCTTGA

>var034_padded
ATGGAGAACGAGCAGAATAACCAAGATTCAGAAAATGGTCTGGATTACTTTAGAAGT AT
GTACGATAGATACAGAGATGTGTTCATAAACCATATTAATGATTACGTATTAGAAGATGA
TATAAAAATTATAATTTCAAAATACTATAAACTATTATTTGATTATAACTGCTTAG GGG
GTAAAAATAATAGAGGAATTTTAGTTATATTAATTTATGAGTATGTAAAGAATAGAGATA
TTAATTGTAATGAGTGGGAAAAAGTGGCTTGTATAG CATGGTGTATAGAAATTTTACAA
GCATCTTTTTTAGTAGCAGATGATATTATGGATAAGGGAGAAACACGCAGAAACAAACAT
TGTTG GTATTTGTTAAAAGACGTTGAAATTAAGAATGCGGTGAATGATGTGTTTCTTCT
GTATAACGCTATATACAA ATTACTTGATGTATATTTGCGCAATGATAACTGTTACCTTG
ATTTAATTACATCCTTTAGAGAAGCCACTTTAAAAACTATAGTAGGACAACATT TAGAT
ACCTTTGGAGATTCTAAAAAGACGGGAAAAGTTGGCTCAGATATTCAAAATAATAAATTA
ACGTGGCCCTTGATAAAA GCATTTGAACTATGTTCACAACCTGAAAAAGAGGACATAAT
AAGAAATTATGGGAAAGATAATGTAACATGTATTAAGTTTATTAATGATATATATGAACA
TTATAATATCAGGGATCATTATGTGGAATATGAAAAGAAGCAGAAGATGAAAATATTAGA
 AGCCATAAACCAATTGCATCATGAAG GTATAGAATATGTCTTGAAATACGTAATGGAC
ATTTTGTTTACAGGCGCTTGA

####
Translation of the annotated and all "padded" variant sequences to amino acid sequences

>Annot_1
MENEQNNQDSENGLDYFRSMYDRYRDVFINHINDYVLEDDIKIIISKYYKLLFDYNCLGG
KNNRGILVILIYEYVKNRDINCNEWEKVACIAWCIEILQASFLVADDIMDKGETRRNKHC
WYLLKDVEIKNAVNDVFLLYNAIYKLLDVYLRNDNCYLDLITSFREATLKTIVGQHLDTN
IFSDKYSHIDKDIDVNNINISQENKININMLNFKVYQNIIIHKTAYYSFFLPIVCGMQMG
GISLDNLLYKKVENIAILMGEYFQVHDDYIDTFGDSKKTGKVGSDIQNNKLTWPLIKAFE
LCSQPEKEDIIRNYGKDNVTCIKFINDIYEHYNIRDHYVEYEKKQKMKILEAINQLHHEG

IEYVLKYVMDILFTGA*

>var009_padded_1
MYDRYRDVFINHINDYVLEDDIKIIISKYYKLLFDYNCLGGKNNRGILVILIYEYVKNRD
INCNEWEKVACIAWCIEILQASFLVADDIMDKGETRRNKHCWYLLKDVEIKNAVNDVFLL
YNAIYKLLDVYLRNDNCYLDLITSFREATLKTIVGQHLDTNIFSDKYSHIDKDIDVNNIN
ISQENKININMLNFKVYQNIIIHKTAYYSFFLPIVCGMQMGGISLDNLLYKKVENIAILM
GEYFQVHDDYIDTFGDSKKTGKVGSDIQNNKLTWPLIKAFELCSQPEKEDIIRNYGKDNV
TCIKFINDIYEHYNIRDHYVEYEKKQKMKILEAINQLHHEGIEYVLKYVMDILFTGA*

>var045_padded_1
MENEQNNQDSENGLDYFRSMYDRYRDVFINHINDYVLEDDIKIIISKYYKLLFDYNCLGG
KNNRGILVILIYEYVKNRDINCNEWEKVACIAWCIEILQASFLVADDIMDKGETRRNKHC
WYLLKDVEIKNAVNDVFLLYNAIYKLLDVYLRNDNCYLDLITSFREATLKTIVGQHLDTN
IFSDKYSHIDKDIDVNNINISQENKININMLNFKVYQNIIIHKTAYYSFFLPIVCGMQMG
GISLDNLLYKKVHDDYIDTFGDSKKTGKVGSDIQNNKLTWPLIKAFELCSQPEKEDIIRN
YGKDNVTCIKFINDIYEHYNIRDHYVEYEKKQKMKILEAINQLHHEGIEYVLKYVMDILF
TGA*

>var169_padded_1
MENEQNNQDSENGLDYFRSMYDRYRDVFINHINDYVLEDDIKIIISKYYKLLFDYNCLGG
KNNRGILVILIYEYVKNRDINCNEWEKVACIAWCIEILQASFLVADDIMDKGETRRNKHC
WYLLKDVEIKNAVNDVFLLYNAIYKLLDVYLRNDNCYLDLITSFREATLKTIVGQHLDTN
IFSDKYSHIDKDIDVNNINISQENKININMLNFKVYQNIIIHKTAYYSFFLPIVCGMQMG
GISLDNLLYKKVENIAILMGEYFQVHDDYIDTFGDSKKTGKVGSDIQNNKLTWPLIKAFE
LCSQPEKEDIIRNYGKDNVTCIKFINDIYEHYNIRDHYVEYEKKQKMKILEAINQLHHEE
YVLKYVMDILFTGA*

>var148_padded_1
MENEQNNQDSENGLDYFRSMYDRYRDVFINHINDYVLEDDIKIIISKYYKLLFDYNCLGG
KNNRGILVILIYEYVKNRDINCNEWEKVACIAWCIEILQASFLVADDIMDKGETRRNKHC
WYLLKDVEIKNAVNDVFLLYNAIYKLLDVYLRNDNCYLDLITSFREATLKTIVGQHLDTN
IFSDKYSHIDKDIDVNNINISQENKININMLNFKVYQNIIIHKTAYYSFFLPIVCGMQMG
GISLDNLLYKKVENIAILMGEYFQVHDDYIDTFGDSKKTGKVGSDIQNNKLTWPLIKAFE
LCSQPEKEDIIRNYGKDNVTCIKFINDIYEHYNIRDHYVEYEKKQKMKILEYRICLEIRN
GHFVYRRLRDNNLNNFCLNMIKFKKKKK*

>var208_padded_1
MENEQNNQDSENGLDYFRSMYDRYRDVFINHINDYVLEDDIMDKGETRRNKHCWYLLKDV
EIKNAVNDVFLLYNAIYKLLDVYLRNDNCYLDLITSFREATLKTIVGQHLDTNIFSDKYS
HIDKDIDVNNINISQENKININMLNFKVYQNIIIHKTAYYSFFLPIVCGMQMGGISLDNL
LYKKVENIAILMGEYFQVHDDYIDTFGDSKKTGKVGSDIQNNKLTWPLIKAFELCSQPEK
EDIIRNYGKDNVTCIKFINDIYEHYNIRDHYVEYEKKQKMKILEAINQLHHEGIEYVLKY
VMDILFTGA*

>var078_padded_1
MENEQNNQDSENGLDYFRSMYDRYRDVFINHINDYVLEDDIKIIISKYYKLLWGKNNRGI
LVILIYEYVKNRDINCNEWEKVACIAWCIEILQASFLVADDIMDKGETRRNKHCWYLLKD
VEIKNAVNDVFLLYNAIYKLLDVYLRNDNCYLDLITSFREATLKTIVGQHLDTNIFSDKY
SHIDKDIDVNNINISQENKININMLNFKVYQNIIIHKTAYYSFFLPIVCGMQMGGISLDN
LLYKKVENIAILMGEYFQVHDDYIDTFGDSKKTGKVGSDIQNNKLTWPLIKAFELCSQPE
KEDIIRNYGKDNVTCIKFINDIYEHYNIRDHYVEYEKKQKMKILEAINQLHHEGIEYVLK
YVMDILFTGA*

>var036_padded_1
MENEQNNQDSENGLDYFRSMYDRYRDVFINHINDYVLEDDIKIIISKYYKLLFDYNCLAW
CIEILQASFLVADDIMDKGETRRNKHCWYLLKDVEIKNAVNDVFLLYNAIYKLLDVYLRN
DNCYLDLITSFREATLKTIVGQHLDTNIFSDKYSHIDKDIDVNNINISQENKININMLNF
KVYQNIIIHKTAYYSFFLPIVCGMQMGGISLDNLLYKKVENIAILMGEYFQVHDDYIDTF
GDSKKTGKVGSDIQNNKLTWPLIKAFELCSQPEKEDIIRNYGKDNVTCIKFINDIYEHYN
IRDHYVEYEKKQKMKILEAINQLHHEGIEYVLKYVMDILFTGA*

>var038_padded_1
MENEQNNQDSENGLDYFRSMYDRYRDVFINHINDYVLEDDIKIIISKYYKLLFDYNCLVA

DDIMDKGETRRNKHCWYLLKDVEIKNAVNDVFLLYNAIYKLLDVYLRNDNCYLDLITSFR
EATLKTIVGQHLDTNIFSDKYSHIDKDIDVNNINISQENKININMLNFKVYQNIIIHKTA
YYSFFLPIVCGMQMGGISLDNLLYKKVENIAILMGEYFQVHDDYIDTFGDSKKTGKVGSD
IQNNKLTWPLIKAFELCSQPEKEDIIRNYGKDNVTCIKFINDIYEHYNIRDHYVEYEKKQ
KMKILEAINQLHHEGIEYVLKYVMDILFTGA*

>var094_padded_1
MENEQNNQDSENGLDYFRSMYDRYRDVFINHINDYVLEDDIKIIISKYYKLLFDYNCLGG
KNNRGILVILIYEYLLKDVEIKNAVNDVFLLYNAIYKLLDVYLRNDNCYLDLITSFREAT
LKTIVGQHLDTNIFSDKYSHIDKDIDVNNINISQENKININMLNFKVYQNIIIHKTAYYS
FFLPIVCGMQMGGISLDNLLYKKVENIAILMGEYFQVHDDYIDTFGDSKKTGKVGSDIQN
NKLTWPLIKAFELCSQPEKEDIIRNYGKDNVTCIKFINDIYEHYNIRDHYVEYEKKQKMK
ILEAINQLHHEGIEYVLKYVMDILFTGA*

>var124_padded_1
MENEQNNQDSENGLDYFRSMYDRYRDVFINHINDYVLEDDIKIIISKYYKLLFDYNCLGG
KNNRGILVILIYEYVKNRDINCIAWCIEILQASFLVADDIMDKGETRRNKHCWYLLKDVE
IKNAVNDVFLLYNAIYKLLDVYLRNDNCYLDLITSFREATLKTIVGQHLDTNIFSDKYSH
IDKDIDVNNINISQENKININMLNFKVYQNIIIHKTAYYSFFLPIVCGMQMGGISLDNLL
YKKVENIAILMGEYFQVHDDYIDTFGDSKKTGKVGSDIQNNKLTWPLIKAFELCSQPEKE
DIIRNYGKDNVTCIKFINDIYEHYNIRDHYVEYEKKQKMKILEAINQLHHEGIEYVLKYV
MDILFTGA*

>var157_padded_1
MENEQNNQDSENGLDYFRSMYDRYRDVFINHINDYVLEDDIKIIISKYYKLLFDYNCLGG
KNNRGILVILIYEYVKNRDINCNEWEKVACIAWCIEILQASFLVADDIMDKGETRRNKHC
WYLLKDVEIKNAVNDVFLLYNAIYKLLDVYLRNDNCYLDLITSFREATLKTIVYQNIIIH
KTAYYSFFLPIVCMQMGGISLDNLLYKKVENIAILMGEYFQVHDDYIDTFGDSKKTGKVG
SDIQNNKLTWPLIKAFELCSQPEKEDIIRNYGKDNVTCIKFINDIYEHYNIRDHYVEYEK
KQKMKILEAINQLHHEGIEYVLKYVMDILFTGA*

>var107_padded_1
MENEQNNQDSENGLDYFRSMYDRYRDVFINHINDYVLEDDIKIIISKYYKLLFDYNCLGG
KNNRGILVILIYEYVKNRDINCNEWEKVACIAWCIEILQASFLVADDIMDKGETRRNKHC
WYLLKDVEIKNAVNDVFLLYNAIYKLLDVYLRNDNCYLDLITSFREATLKTIVYQNIIIH
KTAYYSFFLPIVCGMQMGGISLDNLLYKKVENIAILMGEYFQVHDDYIDTFGDSKKTGKV
GSDIQNNKLTWPLIKAFELCSQPEKEDIIRNYGKDNVTCIKFINDIYEHYNIRDHYVEYE
KKQKMKILEAINQLHHEGIEYVLKYVMDILFTGA*

>var060_padded_1
MENEQNNQDSENGLDYFRSMYDRYRDVFINHINDYVLEDDIKIIISKYYKLLFDYNCLGG
KNNRGILVILIYEYVKNRDINCNEWEKVACIAWCIEILQASFLVADDIMDKGETRRNKHC
WYLLKDVEIKNAVNDVFLLYNAIYKLLDVYLRNDNCYLDLITSFREATLKTIVGQHLDTN
IFSDKYSHIDKDIDVNNINISQENKININMLNFKVYQNIIIHKTAYYSFFLPIVCMQMGG
ISLDNLLYKKVENIAILMGEYFQVHDDYIDTFGDSKKTGKVGSDIQNNKLTWPLIKAFEL
CSQPEKEDIIRNYGKDNVTCIKFINDIYEHYNIRDHYVEYEKKQKMKILEAINQLHHEGI
EYVLKYVMDILFTGA*

>var067_padded_1
MENEQNNQDSENGLDYFRSMYDRYRDVFINHINDYVLEDDIKIIISKYYKLLFDYNCLGG
KNNRGILVILIYEYVKNRDINCNEWEKVACIAWCIEILQASFLVADDIMDKGETRRNKHC
WYLLKDVEIKNAVNDVFLLYNAIYKLLDVYLRNDNCYLDLITSFREATLKTIVGQHLDTN
IFSDKYSHIDKDIDVNNINISQENKININMLNFKVYQNIIIHKTAYYSFFLPIVCVCKWG
VYHWTIYYTKRSKI*

>var156_padded_1
MENEQNNQDSENGLDYFRSMYDRYRDVFINHINDYVLEDDIKIIISKYYKLLFDYNCLGG
KNNRGILVILIYEYVKNRDINCNEWEKVACIAWCIEILQASFLVADDIMDKGETRRNKHC
WYLLKDVEIKNAVNDVFLLYNAIYKITSFREATLKTIVGQHLDTNIFSDKYSHIDKDIDV
NNINISQENKININMLNFKVYQNIIIHKTAYYSFFLPIVCGMQMGGISLDNLLYKKVENI
AILMGEYFQVHDDYIDTFGDSKKTGKVGSDIQNNKLTWPLIKAFELCSQPEKEDIIRNYG
KDNVTCIKFINDIYEHYNIRDHYVEYEKKQKMKILEAINQLHHEGIEYVLKYVMDILFTG
A*

>var151_padded_1
MENEQNNQDSENGLDYFRSMYDRYRDVFINHINDYVLEDDIKIIISKYYKLLFDYNCLGG
KNNRGILVILIYEYVKNRDINCNEWEKVACIAWCIEILQASFLVADDIMDKGETRRNKHC
WYLLKDVEIKNAVNDVFLLYNAIYKEATLKTIVGQHLDTNIFSDKYSHIDKDIDVNNINI
SQENKININMLNFKVYQNIIIHKTAYYSFFLPIVCGMQMGGISLDNLLYKKVENIAILMG
EYFQVHDDYIDTFGDSKKTGKVGSDIQNNKLTWPLIKAFELCSQPEKEDIIRNYGKDNVT
CIKFINDIYEHYNIRDHYVEYEKKQKMKILEAINQLHHEGIEYVLKYVMDILFTGA*

>var128_padded_1
MENEQNNQDSENGLDYFRSMYDRYRDVFINHINDYVLEDDIKIIISKYYKLLFDYNCLGG
KNNRGILVILIYEYVKNRDINCNEWEKVACIAWCIEILQASFLVADDIMDKGETRRNKHC
*LLDVYLRNDNCYLDLITSFREATLKTIVGQHLDTNIFSDKYSHIDKDIDVNNINISQEN
KININMLNFKVYQNIIIHKTAYYSFFLPIVCGMQMGGISLDNLLYKKVENIAILMGEYFQ
VHDDYIDTFGDSKKTGKVGSDIQNNKLTWPLIKAFELCSQPEKEDIIRNYGKDNVTCIKF
INDIYEHYNIRDHYVEYEKKQKMKILEAINQLHHEGIEYVLKYVMDILFTGA*

>var075_padded_1
MENEQNNQDSENGLDYFRSMYDRYRDVFINHINDYVLEDDIKIIISKYYKLLFDYNCLGG
KNNRGILVILIYEYVKNRDINCNEWEKVACIAWCIEILQASFLVADDIMDKGETRRNKHC
*EATLKTIVGQHLDTNIFSDKYSHIDKDIDVNNINISQENKININMLNFKVYQNIIIHKT
AYYSFFLPIVCGMQMGGISLDNLLYKKVENIAILMGEYFQVHDDYIDTFGDSKKTGKVGS
DIQNNKLTWPLIKAFELCSQPEKEDIIRNYGKDNVTCIKFINDIYEHYNIRDHYVEYEKK
QKMKILEAINQLHHEGIEYVLKYVMDILFTGA*

>var147_padded_1
MENEQNNQDSENGLDYFRSMYDRYRDVFINHINDYVLEDDIKIIISKYYKLLFDYNCLGG
KNNRGILVILIYEYVKNRDINCNEWEKVACIEILQASFLVADDIMDKGETRRNKHCWYLL
KDVEIKNAVNDVFLLYNAIYKLLDVYLRNDNCYLDLITSFREATLKTIVGQHLDTNIFSD
KYSHIDKDIDVNNINISQENKININMLNFKVYQNIIIHKTAYYSFFLPIVCGMQMGGISL
DNLLYKKVENIAILMGEYFQVHDDYIDTFGDSKKTGKVGSDIQNNKLTWPLIKAFELCSQ
PEKEDIIRNYGKDNVTCIKFINDIYEHYNIRDHYVEYEKKQKMKILEAINQLHHEGIEYV
LKYVMDILFTGA*

>var188_padded_1
MENEQNNQDSENGLDYFRSMYDRYRDVFINHINDYVLEDDIKIIISKYYKLLFDYNCLGG
KNNRGILVILIYEYVKNRDINCNEWEKVACIVADDIMDKGETRRNKHCWYLLKDVEIKNA
VNDVFLLYNAIYKLLDVYLRNDNCYLDLITSFREATLKTIVGQHLDTNIFSDKYSHIDKD
IDVNNINISQENKININMLNFKVYQNIIIHKTAYYSFFLPIVCMQMGGISLDNLLYKKVE
NIAILMGEYFQVHDDYIDTFGDSKKTGKVGSDIQNNKLTWPLIKAFELCSQPEKEDIIRN
YGKDNVTCIKFINDIYEHYNIRDHYVEYEKKQKMKILEAINQLHHEGIEYVLKYVMDILF
TGA*

>var017_padded_1
MENEQNNQDSENGLDYFRSMYDRYRDVFINHINDYVLEDDIKIIISKYYKLLFDYNCLGG
KNNRGILVILIYEYVKNRDINCNEWEKVACIVADDIMDKGETRRNKHCWYLLKDVEIKNA
VNDVFLLYNAIYKLLDVYLRNDNCYLDLITSFREATLKTIVGQHLDTNIFSDKYSHIDKD
IDVNNINISQENKININMLNFKVYQNIIIHKTAYYSFFLPIVCGMQMGGISLDNLLYKKV
ENIAILMGEYFQVHDDYIDTFGDSKKTGKVGSDIQNNKLTWPLIKAFELCSQPEKEDIIR
NYGKDNVTCIKFINDIYEHYNIRDHYVEYEKKQKMKILEAINQLHHEGIEYVLKYVMDIL
FTGA*

>var189_padded_1
MENEQNNQDSENGLDYFRSMYDRYRDVFINHINDYVLEDDIKIIISKYYKLLFDYNCLGG
KNNRGILVILIYEYVKNRDINCNEWEKVACIADDIMDKGETRRNKHCWYLLKDVEIKNAV
NDVFLLYNAIYKLLDVYLRNDNCYLDLITSFREATLKTIVGQHLDTNIFSDKYSHIDKDI
DVNNINISQENKININMLNFKVYQNIIIHKTAYYSFFLPIVCGMQMGGISLDNLLYKKVE
NIAILMGEYFQVHDDYIDTFGDSKKTGKVGSDIQNNKLTWPLIKAFELCSQPEKEDIIRN
YGKDNVTCIKFINDIYEHYNIRDHYVEYEKKQKMKILEAINQLHHEGIEYVLKYVMDILF
TGA*

>var116_padded_1
MENEQNNQDSENGLDYFRSMYDRYRDVFINHINDYVLEDDIKIIISKYYKLLFDYNCLVI
LIYEYVKNRDINCNEWEKVACIAWCIEILQASFLVADDIMDKGETRRNKHCWYLLKDVEI
KNAVNDVFLLYNAIYKLLDVYLRNDNCYLDLITSFREATLKTIVGQHLDTNIFSDKYSHI

DKDIDVNNINISQENKININMLNFKVYQNIIIHKTAYYSFFLPIVCGMQMGGISLDNLLY
KKVENIAILMGEYFQVHDDYIDTFGDSKKTGKVGSDIQNNKLTWPLIKAFELCSQPEKED
IIRNYGKDNVTCIKFINDIYEHYNIRDHYVEYEKKQKMKILEAINQLHHEGIEYVLKYVM
DILFTGA*

>var218_padded_1
MENEQNNQDSENGLDYFRSMYDRYRDVFINHINDYVLEDDIKIIISKYYKLLFDYNCLGK
IRGKNNRGILVILIYEYVKNRDINCNEWEKVACIAWCIEILQASFLVADDIMDKGETRRN
KHCWYLLKDVEIKNAVNDVFLLYNAIYKLLDVYLRNDNCYLDLITSFREATLKTIVGQHL
DTNIFSDKYSHIDKDIDVNNINISQENKININMLNFKVYQNIIIHKTAYYSFFLPIVCGM
QMGGISLDNLLYKKVENIAILMGEYFQVHDDYIDTFGDSKKTGKVGSDIQNNKLTWPLIK
AFELCSQPEKEDIIRNYGKDNVTCIKFINDIYEHYNIRDHYVEYEKKQKMKILEAINQLH
HEGIEYVLKYVMDILFTGA*

>var115_padded_1
MENEQNNQDSENGLDYFRSMYDRYRDVFINHINDYVLEDDIKIIISKYYKLLFDYNCLGG
KNNRGILVILIYEYVKNRDINCNEWEKVACIAWCIEILQASFLVADDIMDKGETRRNKHC
WYLLKDVEIKNAVNDVFLLLLDVYLRNDNCYLDLITSFREATLKTIVGQHLDTNIFSDKY
SHIDKDIDVNNINISQENKININMLNFKVYQNIIIHKTAYYSFFLPIVCGMQMGGISLDN
LLYKKVENIAILMGEYFQVHDDYIDTFGDSKKTGKVGSDIQNNKLTWPLIKAFELCSQPE
KEDIIRNYGKDNVTCIKFINDIYEHYNIRDHYVEYEKKQKMKILEAINQLHHEGIEYVLK
YVMDILFTGA*

>var062_padded_1
MENEQNNQDSENGLDYFRSMYDRYRDVFINHINDYVLEDDIKIIISKYYKLLFDYNCLGG
KNNRGILVILIYEYVKNRDINCNEWEKVACIAWCIEILQASFLVADDIMDKGETRRNKHC
WYLLKDVEIKNAVNDVFLLYNAIYKLLDVYLRNDNCYLDLITSFREATLKTIVGQHLDTN
IFSDKYSHIDKDIDVNNINISQENKININMLNFKVYQNIIIHKTAYYSFFLPIVCGMQMG
GISLDNLLYKKVENIAILMGEYFQVHDDYIDTFGDSKKTGKAFELCSQPEKEDIIRNYGK
DNVTCIKFINDIYEHYNIRDHYVEYEKKQKMKILEAINQLHHEGIEYVLKYVMDILFTGA
*

>var310_padded_1
MVWITLEVYRDVFINHINDYVLEDDIKIIISKYYKLLFDYNCLGGKNNRGILVILIYEYV
KNRDINCNEWEKVACIAWCIEILQASFLVADDIMDKGETRRNKHCWYLLKDVEIKNAVND
VFLLYNAIYKLLDVYLRNDNCYLDLITSFREATLKTIVGQHLDTNIFSDKYSHIDKDIDV
NNINISQENKININMLNFKVYQNIIIHKTAYYSFFLPIVCMQMGGISLDNLLYKKVENIA
ILMGEYFQVHDDYIDTFGDSKKTGKVGSDIQNNKLTWPLIKAFELCSQPEKEDIIRNYGK
DNVTCIKFINDIYEHYNIRDHYVEYEKKQKMKILEAINQLHHEGIEYVLKYVMDILFTGA
*

>var311_padded_1
MVWITLEVYRDVFINHINDYVLEDDIKIIISKYYKLLFDYNCLGGKNNRGILVILIYEYV
KNRDINCNEWEKVACIAWCIEILQASFLVADDIMDKGETRRNKHCWYLLKDVEIKNAVND
VFLLYNAIYKLLDVYLRNDNCYLDLITSFREATLKTIVGQHLDTNIFSDKYSHIDKDIDV
NNINISQENKININMLNFKVYQNIIIHKTAYYSFFLPIVCGMQMGGISLDNLLYKKVENI
AILMGEYFQVHDDYIDTFGDSKKTGKVGSDIQNNKLTWPLIKAFELCSQPEKEDIIRNYG
KDNVTCIKFINDIYEHYNIRDHYVEYEKKQKMKILEAINQLHHEGIEYVLKYVMDILFTG
A*

>var025_padded_1
MENEQNNQDSENGLDYFRSMYDRYRDVFINHINDYVLEDDIKIIISKYYKLLFDYNCLGG
KNNRGILVILIYEYVKNRDINCNEWEKVACIAWCIEILQASFLVADDIMDKGETRRNKHC
WYLLKDVEIKNAVNDVFLLYNAIYKLLDVYLRNDNCYLDLITSFREATLKTIVGQHLDTN
IFSDKYSHIDKDIDVNNINISQEINFKVYQNIIIHKTAYYSFFLPIVCMQMGGISLDNLL
YKKVENIAILMGEYFQVHDDYIDTFGDSKKTGKVGSDIQNNKLTWPLIKAFELCSQPEKE
DIIRNYGKDNVTCIKFINDIYEHYNIRDHYVEYEKKQKMKILEAINQLHHEGIEYVLKYV
MDILFTGA*

>var267_padded_1
MENEQNNQDSENGLDYFRSMYDRYRDVFINHINDYVLEDDIKIIISKYYKLLFDYNCLGG
KNNRGILVILIYEYVKNRDINCNEWEKVACIAWCIEILQASFLVADDIMDKGETRRNKHC
WYLLKDVEIKNAVNDVFLLYNAIYKLLDVYLRNDNCYLDLITSFREATLKTIVGQHLDTN
IFSDKYSHIDKDIDVNNINISQENKININMLNFKVYQNIIIHKTAYLFILGMQMGGISLD

NLLYKKVENIAILMGEYFQVHDDYIDTFGDSKKTGKVGSDIQNNKLTWPLIKAFELCSQP
EKEDIIRNYGKDNVTCIKFINDIYEHYNIRDHYVEYEKKQKMKILEAINQLHHEGIEYVL
KYVMDILFTGA*

>var235_padded_1
MENEQNNQDSENGLDYFRSMYDRYRDVFINHINDYVLEDDIKIIISKYYKLLFDYNCLGG
KNNRGILVILIYEYVKNRDINCNEWEKVACIAWCIEILQASFLVADDIMDKGETRRNKHC
WYLLKDVEIKNAVNDVFLLYNAIYKLLDVYLRNDNCYLDLITSFREATLKTIVGQHLDTN
IFSDKYSHIDKDIDVNNINISQENKININMLNFKVYQNIIIHKTAYYSFFLPIVCGMQMG
GISLDNLLYKKVENIAILMGEYFQVHDDYIDTFGDSKKTGKVGSDIQNNKLTWPLIK*AF
ELCSQPEKEDIIRNYGKDNVTCIKFINDIYEHYNIRDHYVEYEKKQKMKILEAINQLHHE
GIEYVLKYVMDILFTGA*

>var001_padded_1
MMYDRYRDVFINHINDYVLEDDIKIIISKYYKLLFDYNCLGGKNNRGILVILIYEYVKNR
DINCNEWEKVACIAWCIEILQASFLVADDIMDKGETRRNKHCWYLLKDVEIKNAVNDVFL
LYNAIYKLLDVYLRNDNCYLDLITSFREATLKTIVGQHLDTNIFSDKYSHIDKDIDVNNI
NISQENKININMLNFKVYQNIIIHKTAYYSFFLPIVCGMQMGGISLDNLLYKKVENIAIL
MGEYFQVHDDYIDTFGDSKKTGKVGSDIQNNKLTWPLIKAFELCSQPEKEDIIRNYGKDN
VTCIKFINDIYEHYNIRDHYVEYEKKQKMKILEAINQLHHEGIEYVLKYVMDILFTGA*

>var024_padded_1
MENEQNNQDSENGLDYFRSMYDRYRDVFINHINDYVLEDDIKIIISKYYKLLFDYNCLGG
KNNRGILVILIYEYVKNRDINCNEWEKVACIAWCIEILQASFLVADDIMDKGETRRNKHC
WYLLKDVEIKNAVNDVFLLYNAIYKLLDVYLRNDNCYLDLITSFREATLKTIVGQHLDTN
IFSDKYSHIDKDIDVNNINISQENKININMLNFKVYQNIIIHKTAYYSFFLPIVCGMQMG
GISLDNLLYKKVENIAILMGEYFQVHDDYIDTFGDSKKTGKVGSDIQNNKLTWPLIKIII
IIFFFL*AFELCSQPEKEDIIRNYGKDNVTCIKFINDIYEHYNIRDHYVEYEKKQKMKIL
EAINQLHHEGIEYVLKYVMDILFTGA*

>var230_padded_1
MENEQNNQDSENGLDYFRSMYDRYRDVFINHINDYVLEDDIKIIISKYYKLLFDYNCLGG
KNNRGILVILIYEYVKNRDINCNEWEKVACIAWCIEILQASFLVADDIMDKGETRRNKHC
WYLLKDVEIKNAVNDVFLLYNAIYKLLDVYLRNDNCYLDLITSFREATLKTIVGQHLDTN
IFSDKYSHIDKDIDVNNINISQENKININMLNFKVYQNIIIHKTAYYSFFLPIVCGMQMG
GISLDNLLYKKVENIAILMGEYFQVHDDYIDTFGDSKKTGKVGSDIQNNKLTWPLIKAFE
LCSQPEKEDIIRNYGKDNVTCIKFINDIYEHYNIRDHYVEYEKKQKMKILDHKPIAS*RY
RICLEIRNGHFVYRRLRDNNLNNFCLNMIKFKKKKK*

>var339_padded_1
MENEQNNQDSENGLDYFRSMYDRYRDVFINHINDYVLEDDIKIIISKYYKLLFDYNCLGG
KNNRGILVILIYEYVKNRDINCNEWEKVACIAWCIEILQASFLVADDIMDKGETRRNKHC
WYLLKDVEIKNAVNDVFLLYNAIYKLLDVYLRNDNCYLDLITSFREATLKTIVGQHLDTN
IFSDKYSHIDKDIDVNNINISQENKININMLNFKVYQNIIIHKTAYYSFFLPIVCGMQMG
GISLDNLLYKKVENIAILMGEYFQVHDDYIDTFGDSKKTGKVGSDIQNNKLTWPLIKAFE
LCSQPEKEDIIRNYGKDNVTCIKFINDIYEHYNIRDHYVEYEKKQKMKILE*

>var357_padded_1
YIFIYEGGKNNRGILVILIYEYVKNRDINCNEWEKVACIAWCIEILQASFLVADDIMDKG
ETRRNKHCWYLLKDVEIKNAVNDVFLLYNAIYKLLDVYLRNDNCYLDLITSFREATLKTI
VGQHLDTNIFSDKYSHIDKDIDVNNINISQENKININMLNFKVYQNIIIHKTAYYSFFLP
IVCGMQMGGISLDNLLYKKVENIAILMGEYFQVHDDYIDTFGDSKKTGKVGSDIQNNKLT
WPLIKAFELCSQPEKEDIIRNYGKDNVTCIKFINDIYEHYNIRDHYVEYEKKQKMKILEA
INQLHHEGIEYVLKYVMDILFTGA*

>var071_padded_1
MENEQNNQDSENGLDYFRRGKNNRGILVILIYEYVKNRDINCNEWEKVACIAWCIEILQA
SFLVADDIMDKGETRRNKHCWYLLKDVEIKNAVNDVFLLYNAIYKLLDVYLRNDNCYLDL
ITSFREATLKTIVGQHLDTNIFSDKYSHIDKDIDVNNINISQENKININMLNFKVYQNII
IHKTAYYSFFLPIVCGMQMGGISLDNLLYKKVENIAILMGEYFQVHDDYIDTFGDSKKTG
KVGSDIQNNKLTWPLIKAFELCSQPEKEDIIRNYGKDNVTCIKFINDIYEHYNIRDHYVE
YEKKQKMKILEAINQLHHEGIEYVLKYVMDILFTGA*

>var006_padded_1

MENEQNNQDSENGLDYFRSMYDRYRDVFINHINDYVLEDDIKIIISKYYKLLFDYNCLDV
LLGGKNNRGILVILIYEYVKNRDINCNEWEKVACIAWCIEILQASFLVADDIMDKGETRR
NKHCWYLLKDVEIKNAVNDVFLLYNAIYKLLDVYLRNDNCYLDLITSFREATLKTIVGQH
LDTNIFSDKYSHIDKDIDVNNINISQENKININMLNFKVYQNIIIHKTAYYSFFLPIVCG
MQMGGISLDNLLYKKVENIAILMGEYFQVHDDYIDTFGDSKKTGKVGSDIQNNKLTWPLI
KAFELCSQPEKEDIIRNYGKDNVTCIKFINDIYEHYNIRDHYVEYEKKQKMKILEAINQL
HHEGIEYVLKYVMDILFTGA*

>var021_padded_1
MENEQNNQDSENGLDYFRSMYDRYRDVFINHINDYVLEDDIKIIISKYYKLLFDYNCLGA
WCIEILQASFLVADDIMDKGETRRNKHCWYLLKDVEIKNAVNDVFLLYNAIYKLLDVYLR
NDNCYLDLITSFREATLKTIVGQHLDTNIFSDKYSHIDKDIDVNNINISQENKININMLN
FKVYQNIIIHKTAYYSFFLPIVCGMQMGGISLDNLLYKKVENIAILMGEYFQVHDDYIDT
FGDSKKTGKVGSDIQNNKLTWPLIKAFELCSQPEKEDIIRNYGKDNVTCIKFINDIYEHY
NIRDHYVEYEKKQKMKILEAINQLHHEGIEYVLKYVMDILFTGA*

>var034_padded_1
MENEQNNQDSENGLDYFRSMYDRYRDVFINHINDYVLEDDIKIIISKYYKLLFDYNCLGG
KNNRGILVILIYEYVKNRDINCNEWEKVACIAWCIEILQASFLVADDIMDKGETRRNKHC
WYLLKDVEIKNAVNDVFLLYNAIYKLLDVYLRNDNCYLDLITSFREATLKTIVGQHLDTF
GDSKKTGKVGSDIQNNKLTWPLIKAFELCSQPEKEDIIRNYGKDNVTCIKFINDIYEHYN
IRDHYVEYEKKQKMKILEAINQLHHEGIEYVLKYVMDILFTGA*

####
Intron retention events observed in the FPPS/GGPPS transcript

>annot+i1 annotated FPPS/GGPPS, with intron 1 retained (length: 108; GC: 16.67%); one exon per line, with the intron added after one blank space in front of its first exon
ATGGAGAACGAGCAGAATAACCAAGATTCAGAAAATGGTCTGGATTACTTTAGAAGT
GTAAGTATAACATGTAGTTATAATAGTATATTTTTATATATGAAGGTATAATATTATATATACAATATATATTGTTTTAT
TATTTTTATTTATTATGTTCCCCTTCAG
ATGTACGATAGATACAGAGATGTGTTCATAAACCATATTAATGATTACGTATTAGAAGATGATATAAAAATTATAATTTC
AAAATACTATAAACTATTATTTGATTATAACTGCTTAG
GGGGTAAAAATAATAGAGGAATTTTAGTTATATTAATTTATGAGTATGTAAAGAATAGAGATATTAATTGTAATGAGTGG
GAAAAAGTGGCTTGTATAG
CATGGTGTATAGAAATTTTACAAGCATCTTTTTTAGTAGCAGATGATATTATGGATAAGGGAGAAACACGCAGAAACAAA
CATTGTTG
GTATTTGTTAAAAGACGTTGAAATTAAGAATGCGGTGAATGATGTGTTTCTTCTGTATAACGCTATATACAA
ATTACTTGATGTATATTTGCGCAATGATAACTGTTACCTTGATTTAATTACATCCTTTAGAGAAGCCACTTTAAAAACTA
TAGTAGGACAACATTTAGATACAAATATATTTTCAGATAAATACTCCCATATAGACAAAGATATAGATGTTAATAATATT
AATATATCTCAAGAGAATAAAATTAATATAAACATGTTAAATTTTAAGGTTTATCAAAACATTATTATTCATAAAACTGC
TTATTATTCATTCTTTTTACCTATTGTTTGTG
GTATGCAAATGGGGGGTATATCATTGGACAATTTATTATACAAAAAGGTCGAAAATATAGCAATTCTTATGGGGGAATAT
TTTCAA
GTCCATGATGATTATATAGATACCTTTGGAGATTCTAAAAAGACGGGAAAAGTTGGCTCAGATATTCAAAATAATAAATT
AACGTGGCCCTTGATAAAA
GCATTTGAACTATGTTCACAACCTGAAAAAGAGGACATAATAAGAAATTATGGGAAAGATAATGTAACATGTATTAAGTT
TATTAATGATATATATGAACATTATAATATCAGGGATCATTATGTGGAATATGAAAAGAAGCAGAAGATGAAAATATTAG
A
AGCCATAAACCAATTGCATCATGAAG
GTATAGAATATGTCTTGAAATACGTAATGGACATTTTGTTTACAGGCGCTTGA

>annot+i2 annotated FPPS/GGPPS, with intron 2 retained (length: 118; GC: 15.25%); one exon per line, with the intron added after one blank space in front of its first exon
ATGGAGAACGAGCAGAATAACCAAGATTCAGAAAATGGTCTGGATTACTTTAGAAGT
ATGTACGATAGATACAGAGATGTGTTCATAAACCATATTAATGATTACGTATTAGAAGATGATATAAAAATTATAATTTC
AAAATACTATAAACTATTATTTGATTATAACTGCTTAG
GTAAGATAAGTATAGATTGAGAAAAGAAGTTTTTAAAAGTATAGCTACACATATGTATAAATAAATAAATAAATAAATAT
ATATATATATATATATATATATGTATATGTCTTATTAG
GGGGTAAAAATAATAGAGGAATTTTAGTTATATTAATTTATGAGTATGTAAAGAATAGAGATATTAATTGTAATGAGTGG

GAAAAAGTGGCTTGTATAG
CATGGTGTATAGAAATTTTACAAGCATCTTTTTTAGTAGCAGATGATATTATGGATAAGGGAGAAACACGCAGAAACAAA
CATTGTTG
GTATTTGTTAAAAGACGTTGAAATTAAGAATGCGGTGAATGATGTGTTTCTTCTGTATAACGCTATATACAA
ATTACTTGATGTATATTTGCGCAATGATAACTGTTACCTTGATTTAATTACATCCTTTAGAGAAGCCACTTTAAAAACTA
TAGTAGGACAACATTTAGATACAAATATATTTTCAGATAAATACTCCCATATAGACAAAGATATAGATGTTAATAATATT
AATATATCTCAAGAGAATAAAATTAATATAAACATGTTAAATTTTAAGGTTTATCAAAACATTATTATTCATAAAACTGC
TTATTATTCATTCTTTTTACCTATTGTTTGTG
GTATGCAAATGGGGGGTATATCATTGGACAATTTATTATACAAAAAGGTCGAAAATATAGCAATTCTTATGGGGGAATAT
TTTCAA
GTCCATGATGATTATATAGATACCTTTGGAGATTCTAAAAAGACGGGAAAAGTTGGCTCAGATATTCAAAATAATAAATT
AACGTGGCCCTTGATAAAA
GCATTTGAACTATGTTCACAACCTGAAAAAGAGGACATAATAAGAAATTATGGGAAAGATAATGTAACATGTATTAAGTT
TATTAATGATATATATGAACATTATAATATCAGGGATCATTATGTGGAATATGAAAAGAAGCAGAAGATGAAAATATTAG
A
AGCCATAAACCAATTGCATCATGAAG
GTATAGAATATGTCTTGAAATACGTAATGGACATTTTGTTTACAGGCGCTTGA

>annot+i4 annotated FPPS/GGPPS, with intron 4 retained (length: 72; GC: 15.28%); one exon per line, with the intron added after one blank space in front of its first exon
ATGGAGAACGAGCAGAATAACCAAGATTCAGAAAATGGTCTGGATTACTTTAGAAGT
ATGTACGATAGATACAGAGATGTGTTCATAAACCATATTAATGATTACGTATTAGAAGATGATATAAAAATTATAATTTC
AAAATACTATAAACTATTATTTGATTATAACTGCTTAG
GGGGTAAAAATAATAGAGGAATTTTAGTTATATTAATTTATGAGTATGTAAAGAATAGAGATATTAATTGTAATGAGTGG
GAAAAAGTGGCTTGTATAG
CATGGTGTATAGAAATTTTACAAGCATCTTTTTTAGTAGCAGATGATATTATGGATAAGGGAGAAACACGCAGAAACAAA
CATTGTTG
GTATGTTTTATGAATGAACACATAAATATATATGTAAATACATATATATATTTTTAATTTTTACCTTTTTAG
GTATTTGTTAAAAGACGTTGAAATTAAGAATGCGGTGAATGATGTGTTTCTTCTGTATAACGCTATATACAA
ATTACTTGATGTATATTTGCGCAATGATAACTGTTACCTTGATTTAATTACATCCTTTAGAGAAGCCACTTTAAAAACTA
TAGTAGGACAACATTTAGATACAAATATATTTTCAGATAAATACTCCCATATAGACAAAGATATAGATGTTAATAATATT
AATATATCTCAAGAGAATAAAATTAATATAAACATGTTAAATTTTAAGGTTTATCAAAACATTATTATTCATAAAACTGC
TTATTATTCATTCTTTTTACCTATTGTTTGTG
GTATGCAAATGGGGGGTATATCATTGGACAATTTATTATACAAAAAGGTCGAAAATATAGCAATTCTTATGGGGGAATAT
TTTCAA
GTCCATGATGATTATATAGATACCTTTGGAGATTCTAAAAAGACGGGAAAAGTTGGCTCAGATATTCAAAATAATAAATT
AACGTGGCCCTTGATAAAA
GCATTTGAACTATGTTCACAACCTGAAAAAGAGGACATAATAAGAAATTATGGGAAAGATAATGTAACATGTATTAAGTT
TATTAATGATATATATGAACATTATAATATCAGGGATCATTATGTGGAATATGAAAAGAAGCAGAAGATGAAAATATTAG
A
AGCCATAAACCAATTGCATCATGAAG
GTATAGAATATGTCTTGAAATACGTAATGGACATTTTGTTTACAGGCGCTTGA

>annot+i7 annotated FPPS/GGPPS, with intron 7 retained (length: 116; GC: 5.17%); one exon per line, with the intron added after one blank space in front of its first exon
ATGGAGAACGAGCAGAATAACCAAGATTCAGAAAATGGTCTGGATTACTTTAGAAGT
ATGTACGATAGATACAGAGATGTGTTCATAAACCATATTAATGATTACGTATTAGAAGATGATATAAAAATTATAATTTC
AAAATACTATAAACTATTATTTGATTATAACTGCTTAG
GGGGTAAAAATAATAGAGGAATTTTAGTTATATTAATTTATGAGTATGTAAAGAATAGAGATATTAATTGTAATGAGTGG
GAAAAAGTGGCTTGTATAG
CATGGTGTATAGAAATTTTACAAGCATCTTTTTTAGTAGCAGATGATATTATGGATAAGGGAGAAACACGCAGAAACAAA
CATTGTTG
GTATTTGTTAAAAGACGTTGAAATTAAGAATGCGGTGAATGATGTGTTTCTTCTGTATAACGCTATATACAA
ATTACTTGATGTATATTTGCGCAATGATAACTGTTACCTTGATTTAATTACATCCTTTAGAGAAGCCACTTTAAAAACTA
TAGTAGGACAACATTTAGATACAAATATATTTTCAGATAAATACTCCCATATAGACAAAGATATAGATGTTAATAATATT
AATATATCTCAAGAGAATAAAATTAATATAAACATGTTAAATTTTAAGGTTTATCAAAACATTATTATTCATAAAACTGC
TTATTATTCATTCTTTTTACCTATTGTTTGTG
GTATGCAAATGGGGGGTATATCATTGGACAATTTATTATACAAAAAGGTCGAAAATATAGCAATTCTTATGGGGGAATAT
TTTCAA
GTTATTATTTAAAAAATATATATATATTTTTTTTTATTTTATTTATTTATTTATTTCATTTTTATATTATCTTTATGAAT
AAATATATACATATAATATAATTTTATTTTTATTAG
GTCCATGATGATTATATAGATACCTTTGGAGATTCTAAAAAGACGGGAAAAGTTGGCTCAGATATTCAAAATAATAAATT

AACGTGGCCCTTGATAAAA
GCATTTGAACTATGTTCACAACCTGAAAAAGAGGACATAATAAGAAATTATGGGAAAGATAATGTAACATGTATTAAGTT
TATTAATGATATATATGAACATTATAATATCAGGGATCATTATGTGGAATATGAAAAGAAGCAGAAGATGAAAATATTAG
A
AGCCATAAACCAATTGCATCATGAAG
GTATAGAATATGTCTTGAAATACGTAATGGACATTTTGTTTACAGGCGCTTGA

>annot+i8 annotated FPPS/GGPPS, with intron 8 retained (length: 112; GC: 9.82%); one exon per line, with the intron added after one blank space in front of its first exon
ATGGAGAACGAGCAGAATAACCAAGATTCAGAAAATGGTCTGGATTACTTTAGAAGT
ATGTACGATAGATACAGAGATGTGTTCATAAACCATATTAATGATTACGTATTAGAAGATGATATAAAAATTATAATTTC
AAAATACTATAAACTATTATTTGATTATAACTGCTTAG
GGGGTAAAAATAATAGAGGAATTTTAGTTATATTAATTTATGAGTATGTAAAGAATAGAGATATTAATTGTAATGAGTGG
GAAAAAGTGGCTTGTATAG
CATGGTGTATAGAAATTTTACAAGCATCTTTTTTAGTAGCAGATGATATTATGGATAAGGGAGAAACACGCAGAAACAAA
CATTGTTG
GTATTTGTTAAAAGACGTTGAAATTAAGAATGCGGTGAATGATGTGTTTCTTCTGTATAACGCTATATACAA
ATTACTTGATGTATATTTGCGCAATGATAACTGTTACCTTGATTTAATTACATCCTTTAGAGAAGCCACTTTAAAAACTA
TAGTAGGACAACATTTAGATACAAATATATTTTCAGATAAATACTCCCATATAGACAAAGATATAGATGTTAATAATATT
AATATATCTCAAGAGAATAAAATTAATATAAACATGTTAAATTTTAAGGTTTATCAAAACATTATTATTCATAAAACTGC
TTATTATTCATTCTTTTTACCTATTGTTTGTG
GTATGCAAATGGGGGGTATATCATTGGACAATTTATTATACAAAAAGGTCGAAAATATAGCAATTCTTATGGGGGAATAT
TTTCAA
GTCCATGATGATTATATAGATACCTTTGGAGATTCTAAAAAGACGGGAAAAGTTGGCTCAGATATTCAAAATAATAAATT
AACGTGGCCCTTGATAAAA
GTATCTTTACAAGAAATATTACATAGACATATATATATTTATATGTATTCATTTTATTTTATATTATATTATATTATATT
TTATTATTATTATTATTTTTTTTTTTTTTGTAG
GCATTTGAACTATGTTCACAACCTGAAAAAGAGGACATAATAAGAAATTATGGGAAAGATAATGTAACATGTATTAAGTT
TATTAATGATATATATGAACATTATAATATCAGGGATCATTATGTGGAATATGAAAAGAAGCAGAAGATGAAAATATTAG
A
AGCCATAAACCAATTGCATCATGAAG
GTATAGAATATGTCTTGAAATACGTAATGGACATTTTGTTTACAGGCGCTTGA

>annot+i10 annotated FPPS/GGPPS, with intron 10 retained (length: 124; GC: 9.68%); one exon per line, with the intron added after one blank space in front of its first exon
ATGGAGAACGAGCAGAATAACCAAGATTCAGAAAATGGTCTGGATTACTTTAGAAGT
ATGTACGATAGATACAGAGATGTGTTCATAAACCATATTAATGATTACGTATTAGAAGATGATATAAAAATTATAATTTC
AAAATACTATAAACTATTATTTGATTATAACTGCTTAG
GGGGTAAAAATAATAGAGGAATTTTAGTTATATTAATTTATGAGTATGTAAAGAATAGAGATATTAATTGTAATGAGTGG
GAAAAAGTGGCTTGTATAG
CATGGTGTATAGAAATTTTACAAGCATCTTTTTTAGTAGCAGATGATATTATGGATAAGGGAGAAACACGCAGAAACAAA
CATTGTTG
GTATTTGTTAAAAGACGTTGAAATTAAGAATGCGGTGAATGATGTGTTTCTTCTGTATAACGCTATATACAA
ATTACTTGATGTATATTTGCGCAATGATAACTGTTACCTTGATTTAATTACATCCTTTAGAGAAGCCACTTTAAAAACTA
TAGTAGGACAACATTTAGATACAAATATATTTTCAGATAAATACTCCCATATAGACAAAGATATAGATGTTAATAATATT
AATATATCTCAAGAGAATAAAATTAATATAAACATGTTAAATTTTAAGGTTTATCAAAACATTATTATTCATAAAACTGC
TTATTATTCATTCTTTTTACCTATTGTTTGTG
GTATGCAAATGGGGGGTATATCATTGGACAATTTATTATACAAAAAGGTCGAAAATATAGCAATTCTTATGGGGGAATAT
TTTCAA
GTCCATGATGATTATATAGATACCTTTGGAGATTCTAAAAAGACGGGAAAAGTTGGCTCAGATATTCAAAATAATAAATT
AACGTGGCCCTTGATAAAA
GCATTTGAACTATGTTCACAACCTGAAAAAGAGGACATAATAAGAAATTATGGGAAAGATAATGTAACATGTATTAAGTT
TATTAATGATATATATGAACATTATAATATCAGGGATCATTATGTGGAATATGAAAAGAAGCAGAAGATGAAAATATTAG
A
AGCCATAAACCAATTGCATCATGAAG
GTAACATATAAATAATTTAATTTTTTTTTTTAATTATTTATTTATACATACATATATATATTATATATATATCAAAAAAT
TTTATGTATTAATATATTTATATCTTCGTTTTTTTTTTTCGTAG
GTATAGAATATGTCTTGAAATACGTAATGGACATTTTGTTTACAGGCGCTTGA

Other introns' data
Intron name   length   GC%
intron_3 149   10.07

```
# intron_5  143   7.69
# intron_9  113   7.08
# intron_6  104   14.42

>annot+i1_1 translation of annotated FPPS/GGPPS, with intron 1 retained
MENEQNNQDSENGLDYFRSVSITCSYNSIFLYMKV*YYIYNIYCFIIFIYYVPLQMYDRY
RDVFINHINDYVLEDDIKIIISKYYKLLFDYNCLGGKNNRGILVILIYEYVKNRDINCNE
WEKVACIAWCIEILQASFLVADDIMDKGETRRNKHCWYLLKDVEIKNAVNDVFLLYNAIY
KLLDVYLRNDNCYLDLITSFREATLKTIVGQHLDTNIFSDKYSHIDKDIDVNNINISQEN
KININMLNFKVYQNIIIHKTAYYSFFLPIVCGMQMGGISLDNLLYKKVENIAILMGEYFQ
VHDDYIDTFGDSKKTGKVGSDIQNNKLTWPLIKAFELCSQPEKEDIIRNYGKDNVTCIKF
INDIYEHYNIRDHYVEYEKKQKMKILEAINQLHHEGIEYVLKYVMDILFTGA*

>annot+i2_1 translation of annotated FPPS/GGPPS, with intron 2 retained
MENEQNNQDSENGLDYFRSMYDRYRDVFINHINDYVLEDDIKIIISKYYKLLFDYNCLGK
ISID*EKKFLKV*LHICINK*INKYIYIYIYICICLIRG*K**RNFSYINL*VCKE*RY*
L**VGKSGLYSMVYRNFTSIFFSSR*YYG*GRNTQKQTLLVFVKRR*N*ECGE*CVSSV*
RYIQIT*CIFAQ**LLP*FNYIL*RSHFKNYSRTTFRYKYIFR*ILPYRQRYRC**Y*YI
SRE*N*YKHVKF*GLSKHYYS*NCLLFILFTYCLWYANGGYIIGQFIIQKGRKYSNSYGG
IFSSP**LYRYLWRF*KDGKSWLRYSK**INVALDKSI*TMFTT*KRGHNKKLWER*CNM
Y*VY**YI*TL*YQGSLCGI*KEAEDENIRSHKPIAS*RYRICLEIRNGHFVYRRLX

>annot+i4_1 translation of annotated FPPS/GGPPS, with intron 4 retained
MENEQNNQDSENGLDYFRSMYDRYRDVFINHINDYVLEDDIKIIISKYYKLLFDYNCLGG
KNNRGILVILIYEYVKNRDINCNEWEKVACIAWCIEILQASFLVADDIMDKGETRRNKHC
WYVL*MNT*IYM*IHIYIFNFYLFRYLLKDVEIKNAVNDVFLLYNAIYKLLDVYLRNDNC
YLDLITSFREATLKTIVGQHLDTNIFSDKYSHIDKDIDVNNINISQENKININMLNFKVY
QNIIIHKTAYYSFFLPIVCGMQMGGISLDNLLYKKVENIAILMGEYFQVHDDYIDTFGDS
KKTGKVGSDIQNNKLTWPLIKAFELCSQPEKEDIIRNYGKDNVTCIKFINDIYEHYNIRD
HYVEYEKKQKMKILEAINQLHHEGIEYVLKYVMDILFTGA*

>annot+i7_1 translation of annotated FPPS/GGPPS, with intron 7 retained
MENEQNNQDSENGLDYFRSMYDRYRDVFINHINDYVLEDDIKIIISKYYKLLFDYNCLGG
KNNRGILVILIYEYVKNRDINCNEWEKVACIAWCIEILQASFLVADDIMDKGETRRNKHC
WYLLKDVEIKNAVNDVFLLYNAIYKLLDVYLRNDNCYLDLITSFREATLKTIVGQHLDTN
IFSDKYSHIDKDIDVNNINISQENKININMLNFKVYQNIIIHKTAYYSFFLPIVCGMQMG
GISLDNLLYKKVENIAILMGEYFQVII*KIYIYFFLFYLFIYFIFILSL*INIYI*YNFI
FIRSMMII*IPLEILKRREKLAQIFKIIN*RGP**KHLNYVHNLKKRT**EIMGKIM*HV
LSLLMIYMNIIISGIIMWNMKRSRR*KY*KP*TNCIMKV*NMS*NT*WTFCLQALX

>annot+i8_1 translation of annotated FPPS/GGPPS, with intron 8 retained
MENEQNNQDSENGLDYFRSMYDRYRDVFINHINDYVLEDDIKIIISKYYKLLFDYNCLGG
KNNRGILVILIYEYVKNRDINCNEWEKVACIAWCIEILQASFLVADDIMDKGETRRNKHC
WYLLKDVEIKNAVNDVFLLYNAIYKLLDVYLRNDNCYLDLITSFREATLKTIVGQHLDTN
IFSDKYSHIDKDIDVNNINISQENKININMLNFKVYQNIIIHKTAYYSFFLPIVCGMQMG
GISLDNLLYKKVENIAILMGEYFQVHDDYIDTFGDSKKTGKVGSDIQNNKLTWPLIKVSL
QEILHRHIYIYMYSFYFILYYIIFYYYYYFFFFVGI*TMFTT*KRGHNKKLWER*CNMY*
VY**YI*TL*YQGSLCGI*KEAEDENIRSHKPIAS*RYRICLEIRNGHFVYRRLX

>annot+i10_1 translation of annotated FPPS/GGPPS, with intron 10 retained
MENEQNNQDSENGLDYFRSMYDRYRDVFINHINDYVLEDDIKIIISKYYKLLFDYNCLGG
KNNRGILVILIYEYVKNRDINCNEWEKVACIAWCIEILQASFLVADDIMDKGETRRNKHC
WYLLKDVEIKNAVNDVFLLYNAIYKLLDVYLRNDNCYLDLITSFREATLKTIVGQHLDTN
IFSDKYSHIDKDIDVNNINISQENKININMLNFKVYQNIIIHKTAYYSFFLPIVCGMQMG
GISLDNLLYKKVENIAILMGEYFQVHDDYIDTFGDSKKTGKVGSDIQNNKLTWPLIKAFE
LCSQPEKEDIIRNYGKDNVTCIKFINDIYEHYNIRDHYVEYEKKQKMKILEAINQLHHEG
NI*II*FFFLIIYLYIHIYIIYISKNFMY*YIYIFVFFFRRYRICLEIRNGHFVYRRLX
```

Additional file 6

Title: Oligonucleotides utilized.

Description: Oligonucleotides used for RNA-seq experiments, amplification of the C-terminal gene region used for plasmid construction, and validation experiments by PCR and RT-PCR.

**Primer name**	**Sequence (5' - 3')**	**Annealing temperature**
F-FPPS/GGPPS-BglII	AGATCTGGTATGCAAATGGGGGGTATA	59°C
R-FPPS/GGPPS-PstI	CTGCAGCAGCGCCTGTAAACAAAATGTC	59°C
F-FPPS/GGPPS-BamHI	CCGGATCCATGGAGAACGAGCAGAATAAC	57°C
F-iso5	GTAGCAGATGATATTATGGATAAGG	55°C
R-iso5	AAAGTGGCTTCTCTAAAGGATG	57°C
F-iso7	CATCCTTTAGAGAAGCCACTTTAA	57°C
R-iso7	AATCATCATGGACCACAAACAATA	58°C
F-iso10	CACAACCTGAAAAAGAGGACAT	58°C
R-iso10	CAAGACATATTCTATACTCTAATATTTTCATC	56°C
F-iso	TTCATTCTTTTTACCTATTGTTTG	53°C
R-iso	CCAGGGCCACGTTAATTT	57°C
